# Two decades of lymphatic filariasis elimination efforts in Nigeria: History, progress, challenges, and prospects for achieving the 2030 targets

**DOI:** 10.1371/journal.pntd.0014860

**Published:** 2026-11-10

**Authors:** Oladele B. Akogun, Hammed Oladeji Mogaji, Babatunde Adewale, Dung D. Pam, Joseph S. Terhemen, Hanafi Oladele, Fatai Oyediran, Chinwe Okoye, Emeka Uzoma, Olumide Falodun, Ununumah Egbelu, Kehinde G. Busari, Sunday Isiyaku, Joy Shuaibu, Anita Gwom, Francisca Olamiju, Emmanuel Miri, Adamu Sallau, Samuel Omoi, Innocent Emereuwa, Rita Oguntoyinbo, Mohammed Aliyu, Esther Ekong, Adamani William, Ijeoma Achu, Osagie Omorogie, Ishaya Rinpan, Cephas Ityonzughul

**Affiliations:** 1 The Health Programme Common Heritage Foundation, Abuja, Nigeria; 2 Public Health Program, Department of Applied and Behavioral Sciences, College of Arts and Sciences, Marian University, Indianapolis, Indiana, United States of America; 3 Department of Public Health and Epidemiology, Nigerian Institute of Medical Research, Yaba, Lagos, Nigeria; 4 Department of Zoology, University of Jos, Jos, Nigeria; 5 Sightsavers, Abuja, Nigeria; 6 Neglected Tropical Diseases Programme, Federal Ministry of Health and Social Welfare, Abuja, Nigeria; 7 Reaching the Last Mile for Onchocerciasis and Lymphatic Filariasis in Africa, Abuja, Nigeria; 8 Mission To Save The Helpless, Ikeja, Lagos, Nigeria; 9 The Carter Center, Jos, Nigeria; 10 Christoffel Blinden Mission International, Abuja, Nigeria; 11 Health and Development Support Programme, Abuja, Nigeria; 12 Amen Health Care and Empowerment Foundation, Abuja, Nigeria; 13 Helen Keller International, Abuja, Nigeria; University of California, Davis, UNITED STATES OF AMERICA

## Abstract

**Introduction:**

Lymphatic filariasis (LF) is one of the priority neglected tropical diseases targeted for elimination by 2030 in 58 endemic countries, including Nigeria, through a dual strategy of interrupting transmission and providing morbidity management and disability prevention (MMDP) services. Only two African countries (Togo and Malawi) have eliminated the disease. The extent to which other African countries have progressed towards elimination is uncertain. It will be helpful to understand the progress endemic countries are making towards elimination. Here, a comprehensive review of the history, progress and prospects for achieving 2030 elimination targets in Nigeria, the most endemic country for LF in Africa was provided. This review will be useful in evaluating the extent of investment that is required before elimination can be achieved in Nigeria.

**Methods:**

Programmatic data was extracted from the National Lymphatic Filariasis Elimination Programme (NLFEP) database for endemicity, treatment history, mass administration of medicines (MAM) coverage, epidemiological assessments, and morbidity management across 774 Local Government Areas (LGAs) in Nigeria. Status of endemic LGAs were identified, and those that had completed a minimum of five effective MAM rounds were assessed on their progress of elimination using the recommended Transmission Assessment Survey (TAS) and Morbidity Management and Disability Prevention (MMDP) guidelines. The number of LGAs expected to achieve LF elimination by 2030 was forecasted. All statistical analyses and visualizations were descriptive and performed in R software (v4.5.2).

**Findings:**

Nigeria completed LF mapping in all 774 LGAs between 2000 and 2011, revealing 583 endemic LGAs. All the endemic LGAs (herein referred to as implementation units (IUs)) received 3–15 rounds of MAM between 2000 and 2015, and 529 (90.7%) completed the required five effective MAM rounds. As of 2024, 445 (76.3%) passed Epidemiological Monitoring Survey (EMS), 346 (59.3%) passed TAS-1 and stopped MAM, while 40 (6.9%) passed TAS-2 and 30 (5.1%) passed TAS-3 (achieve elimination). By 2030, we project that MAM would have stopped in 497 Evaluation Units (EUs) i.e., 85.2% of all IUs, and 309 (62.2%) of those would have passed TAS-3 (achieve elimination). However, under a conservative assumption that 3–5% of planned surveys can fail, these figures could decline to 454 (77.9%) EUs stopping MAM and 284 achieving elimination. For MMDP, Lymphoedema was reported in 450 IUs, totaling 7,824 cases, with 5,828 (74.5%) actively managed. Hydrocele was reported across 503 IUs, with 22,195 documented cases, 72.4% of which received management.

**Conclusion:**

Nigeria has made substantial progress toward the elimination of lymphatic filariasis. The overall trajectory suggests that Nigeria remains on course to stop MAM and achieve elimination in 85% and 53% of all IUs, respectively.

## Author summary

Lymphatic filariasis (LF) is a mosquito-borne disease that can cause severe and lifelong disability, including limb swelling and scrotal enlargement. Nigeria bears the largest LF burden in Africa, making progress in the country critical to achieving global elimination. We reviewed 24 years (2000–2024) of national programme data to assess Nigeria’s progress toward the 2030 elimination target. Our analysis shows that Nigeria has achieved sustained treatment coverage, made substantial progress in interrupting transmission, and expanded access to quality care for people already affected by LF. Under conservative assumptions, our projections indicate that most endemic areas could reach elimination if planned activities continue. However, realizing this potential will require sustained political commitment, partnership and financial support to conduct timely surveys, strengthen surveillance, and scale up morbidity management and disability prevention services.

## Introduction

Lymphatic filariasis (LF) is one of the most dreadful neglected tropical diseases (NTDs). The disease is caused by parasitic filarial nematodes; *Wuchereria bancrofti*, *Brugia Malayi*, or *Brugia timori*, and are transmitted by various mosquito genera, including Anopheles, Culex, and Aedes [1,2]. Infection may be acquired during childhood and deteriorate over the years leading to a chronic damage to the lymphatic system [1]. The disease may result in tissue swelling (lymphoedema), skin thickening (elephantiasis), or scrotal enlargement (hydrocoele) which are painful and profoundly disfiguring [1–3]. Lymphatic filariasis morbidity have been implicated to worsen individuals’ quality of life and economic productivity due to the interference with the physical and psychological health [1–3]. With over 1 billion people at risk of infections across 80 countries and territories, WHO (1997) classified LF as a public health priority, and established the Global Programme to Eliminate Lymphatic Filariasis (GPELF) in 2000 to steer intervention activities towards eliminating the disease [4].

The GPELF has a dual mandate, to interrupt transmission in areas where endemicity is greater than or equal to 1% and to manage morbidity and prevent disability among those affected [5,6]. Transmission interruption relies primarily on preventive chemotherapy, which involves large-scale, annual administration of a single dose of albendazole, combined with either diethylcarbamazine (or ivermectin is used where LF is co-endemic with onchocerciasis) [1,2,5,6]. This strategy is widely referred to as mass administration of medicines (MAM) [1]. Disease control programs are expected to implement a minimum of five rounds of effective MAM annually [7], which is defined as reaching the expected geographic and therapeutic coverage. Geographic coverage is the number of endemic areas by MAM served, while therapeutic coverage is the number of at-risk persons in a defined area that swallowed the distributed medicines [7]. Geographic coverage of 100% and a therapeutic coverage of 65% are necessary to achieve an estimated 99% reduction in prevalence for parasite transmission interruption [8,9]. For morbidity management and disability prevention (MMDP), the GPELF encourages identification of cases, and promotion of hygiene services and training on daily washing, exercise and skin care for individuals with lymphedema and surgical interventions for those with hydrocoele to enhance quality of life and mitigate the long- term impact of the disease [10–12].

The GPELF program was adopted globally and implemented through successive roadmaps [2], with the latest outlining ambitious targets that all 72 countries with a history of LF should have stopped MAM by 2030, with 80% of endemic countries, i.e., 58 countries should have be certified to have eliminated LF as a public health problem by 2030 [2]. More than 10 billion cumulative treatments have been delivered during MAM campaigns to 943.4 million people around the world since 2000 [13], and the number of people requiring MAM has significantly declined by half (485.4 million now in need of MAM) [13]. As at 2024, of all 72 endemic countries, 1 (Gabon) was yet to commence MAM, four other countries had started MAM but not at scale to all endemic areas, 30 (Nigeria inclusive) had started MAM across all endemic areas, and 37 had met the target and no longer require MAM [13]. Sixteen of the latter group are under post treatment surveillance (PTS) but had not been validated and 21 countries have met the criteria for elimination of LF as a public health problem and are in the post-validation surveillance (PVS) phase [13].

Despite the progress made globally, there are still significant gaps as 35 of 72 (~49%) countries are yet to stop MAM, and 37 (~64%) are yet to eliminate LF transmission [13]. Most of the endemic countries (34 of 72 countries) are in the African region, and only 15 of those have stopped MAM, and just 2 of them (Togo and Malawi) have successfully eliminated LF transmission [13]. Therefore, achieving the global elimination targets will require an additional 19 (~54% of global target) and 25 (~68% of global target) African countries to discontinue MAM, and eliminate LF transmission by 2030, respectively. It is thus important that African countries intensify programmatic efforts as the mandate of achieving these targets would heavily be shaped by results from the region. Nigeria bears the largest burden of LF in Africa and is the third most endemic country globally [14–17]. Of the estimated 50 million people affected in sub-Saharan Africa, more than 29 million reside in Nigeria [15,17,18]. Therefore, a critical appraisal of national programmatic activities is essential for understanding progress toward LF elimination and assessing the feasibility of achieving the 2030 elimination targets. Accordingly, this study synthesizes and reviews the implementation of LF elimination efforts in Nigeria, documents progress made toward the 2030 goals and highlights persistent operational and programmatic challenges that require urgent attention to sustain and accelerate elimination efforts.

## Methods

### Study design

This study was a retrospective secondary analysis of national programmatic data, supplemented by secondary analysis of population-based survey data. The analysis was undertaken as part of a technical advisory role to the Nigerian National Lymphatic Filariasis Elimination Programme (NLFEP) Expert Committee. Routinely collected programmatic data generated by the NLFEP between 2000 and 2025 were analyzed to characterize Nigeria’s progress along the lymphatic filariasis elimination pathway, including epidemiological mapping, implementation of preventive chemotherapy, and programmatic assessments used to inform decisions on stopping mass drug administration and post-treatment surveillance. These data were complemented by secondary analysis of population-based data from the 2025 Nigeria Demographic and Health Survey (NDHS), which provided contextual information on insecticide-treated net (ITN) coverage, ownership and use.

### Extraction of study indicators

The programmatic data extracted from the NLFEP database included endemicity classification, treatment history, MAM implementation and coverage, MMDP activities, epidemiological assessment findings, and planned assessment activities for all IUs in Nigeria (see S1 and S2 Data).

For MMDP, both quantitative datasets and qualitative programmatic reports relating to health-system strengthening activities, including capacity development and health-facility readiness assessments, were reviewed and summarized. Additionally, available data on lymphoedema and hydrocoele cases identified and managed between 2021 and 2025 were extracted from national program reports.

Although vector control activities are not currently included within the core implementation framework of the NLFEP, relevant indicators on ITN ownership, access, and utilization were extracted from the most recent Nigeria Demographic and Health Survey (NDHS) 2023–24 report [19]. Variables of interest included the proportion of households owning at least one ITN, the average number of ITNs per household, the proportion of households with at least one ITN for every two household members, and the proportion of children under five years of age and pregnant women who slept under an ITN the night preceding the survey. These indicators were used to provide contextual information on malaria vector-control coverage and its potential relevance to lymphatic filariasis transmission dynamics.

### Study population and sample sizes

This study utilized routinely collected programmatic records encompassing different populations according to the specific LF programme activity. Baseline epidemiological mapping was conducted among adult populations, while MAM targeted eligible populations aged ≥5 years in endemic implementation units (IUs). MMDP activities focused on individuals affected by LF-related morbidity. Epidemiological monitoring assessments conducted before TAS involved populations aged ≥5 years under the applicable pre-TAS protocol, while the current EMS protocol targets adults aged ≥20 years. TAS primarily assesses children aged 6–7 years. No separate sample-size calculation was performed for the present retrospective analysis because all eligible programme records available during the study period were included. Sample sizes for the individual epidemiological assessments, including EMS/pre-TAS and TAS, were determined at the time of their implementation in accordance with the applicable WHO-recommended survey procedures [11].

### Data management and analysis

For MAM data, all LF-endemic LGAs in each of the six Nigeria’s geopolitical zones were identified from program’s inception and their treatment progress was assessed by quantifying the number of effective MAM rounds that have been conducted. The IUs were classified as either having received fewer than five effective rounds or having completed five or more effective rounds, which is the minimum threshold required before conducting EMS. Following that, the transmission status of each IUs was assessed using results from the four epidemiological assessments (EMS and TAS 1–3), and IUs were classified as having passed any of these surveys.

Furthermore, the list of all past and planned epidemiological assessments were extracted to estimate historical failure rate and forecast the feasibility of achieving LF elimination targets by the 2030 endline. Projections were conducted at the geopolitical zone level and were based on LGA-specific starting assessment stage and calendar year. In line with WHO guidance, a two-year interval between successive assessment stages was assumed. The LGAs passing EMS were projected to undergo TAS-1 after two years; those passing TAS-1 were projected to undergo TAS-2 after a further two years; and those passing TAS-2 were projected to undergo TAS-3 after an additional two years. The TAS-3 was treated as the terminal elimination milestone, with no further progression beyond this stage. Projections were extended through 2032 to identify LGAs whose progression would spill beyond the 2030 elimination endline. Trajectory plots were constructed to visualize projected progression across assessment stages over time, with diagonal paths representing biennial advancement and point sizes proportional to the number of IUs.

Finally, the feasibility of meeting elimination targets was assessed under the conditions that all planned assessments were successful (i.e., not limited by funding, or security challenges, and all LGAs passed the survey), or unsuccessful (i.e., about 3–5% of the LGAs fail the survey, or assessment were impacted by insecurity and funding). Results were summarized using stacked bar charts by geopolitical zone for each failure-rate scenario.

For MMDP and NDHS data, indicators were summarized using descriptive statistics such as frequencies and percentage across the states and geopolitical zones, and where applicable visualizations were done using maps and line graphs. All data management, analyses, projections, and visualizations were conducted using R software (version 4.5.2) and ArcGIS software (version 10.8).

### Data presentation

The results are organized by key programmatic phases of the NLFEP. For each phase, relevant contextual and implementation information is first presented to provide background on the intervention, its objectives, implementation requirements, and operational considerations. This is subsequently followed by the presentation of quantitative findings derived from the program data. This approach was adopted to facilitate interpretation of the results within the broader context of programme implementation and progress toward lymphatic filariasis elimination in Nigeria.

## Results

### Overview of Nigeria’s landscape and Lymphatic Filariasis Intervention Strategy

Nigeria is arguably one of the largest and most populous country in Africa, with approximately 923,770 km^2^ land mass and over 230 million people [20].. Given its large landmass and population, Nigeria is administratively organized into six geopolitical zones comprising 36 states and the Federal Capital Territory [21]. These are further subdivided into 774 LGAs, which serve as the primary IUs for LF control [21]. The strategy employed in Nigeria follows the World Health Organization–recommended comprehensive elimination strategy, which comprises both interruption of transmission and MMDP [1,11], and is implemented through a constituted NLFEP.

The NLFEP was established in 1997 and operated through a multi-level governance structure that promoted synergies across the workforce at national and state levels, supported by a national steering committee responsible for technical oversight. Implementation of MAM and MMDP services leveraged the existing NTDs workforce at both state and implementation-unit levels, operating under national directives and technical guidance provided by NFLEP. Resource mobilization and strategic direction followed a top–down model, while operational implementation—including localization of strategies and adaptation to contextual realities—was decentralized to state and IUs. Program activities (meetings, trainings, coverage surveys, MAM, MMDP, and epidemiological assessments such as baseline mapping, EMS and TAS 1–3) were largely supported through external funding secured by international and indigenous NGDOs. Implementation of MAM and MMDP activities was decentralized to the state level, whereas monitoring and evaluation activities, including EMS and TAS were centrally coordinated by the national programme team. This centralized evaluation approach ensures methodological consistency and minimizes potential bias associated with local program ownership. Consequently, evaluation processes functioned as external assessments of program performance at subnational levels. Throughout the program cycle, the NLFEP Expert Committee provided continuous technical advisory support, ensuring alignment with national elimination goals and global LF control strategies.

### Baseline mapping of LF infections in Nigeria

Mapping is the first programmatic step in LF elimination and is used to assess the disease situation in a country and to identify areas in which MAM is required by determining where active transmission was occurring. Mapping of LF in Nigeria was completed between 2000 and 2011 (Table 1). Briefly, it involves a rapid assessment of 50–100 adults (aged 15 years and older) across two high-risk villages selected based on the presence of chronic morbidity, high mosquito density, or other ecological indicators. Blood samples, usually collected by capillary action, were tested for microfilaremia or antigenemia, and decisions to implement MAM were made when prevalence was up ≥1% [22–24]. Although these guidelines were widely used in the early stages of GPELF, concerns have emerged regarding their sensitivity [25], and this has led to the development of a more robust confirmatory mapping method that prioritizes the inclusion of more sampling sites (30 sites against previous two sites), participants (480 children aged 9–14 years against previous 100 adults) to improve the reliability of mapping and decision-making [25]. The NFLEP has conducted confirmatory mapping in some areas classified as non-endemic [26], with findings in consonance with previous mapping results.

**Table 1:** Baseline endemicity profile of lymphatic filariasis in Nigeria as of 2024.

Zone	State	Population	No of LGA	No. of LGAs Mapped (%)	Non-Endemic LGAs (%)	Endemic LGAs (%)	Date of Mapping
**South-east**	Abia	4312266	17	17 (100.0)	0 (0.0)	17 (100.0)	2008
	Anambra	6363454	21	21 (100.0)	0 (0.0)	21 (100.0)	2007-2010
	Ebonyi	3307238	13	13 (100.0)	4 (30.8)	9 (69.2)	2005-2008
	Enugu	4956364	17	17 (100.0)	3 (17.6)	14 (82.4)	2005-2011
	Imo	5987413	27	27 (100.0)	0 (0.0)	27 (100.0)	2007-2010
	Total	24926735	95	95 (100.0)	7 (7.4)	88 (92.6)	2005-2011
**South-west**	Ekiti	3626956	16	16 (100.0)	0 (0.0)	16 (100.0)	2003-2011
	Lagos	13715153	20	20 (100.0)	14 (70.0)	6 (30.0)	2003-2011
	Ogun	5672743	20	20 (100.0)	6 (30.0)	14 (70.0)	2003-2011
	Ondo	5235923	18	18 (100.0)	1 (5.6)	17 (94.4)	2003-2009
	Osun	5209315	30	30 (100.0)	3 (10.0)	27 (90.0)	2003-2013
	Oyo	8508262	33	33 (100.0)	23 (69.7)	10 (30.3)	2003-2013
	Total	41968352	137	137 (100.0)	47 (34.3)	90 (65.7)	2003-2013
**South-South**	Akwa Ibom	5965062	31	31 (100.0)	20 (64.5)	11 (35.5)	2009-2010
	Bayelsa	2591862	8	8 (100.0)	1 (12.5)	7 (87.5)	2009-2010
	Cross River	4395906	18	18 (100.0)	8 (44.4)	10 (55.6)	2009-2010
	Delta	6236189	25	25 (100.0)	9 (36.0)	16 (64.0)	2003-2011
	Edo	4897071	18	18 (100.0)	11 (61.1)	7 (38.9)	2008-2011
	Rivers	7890201	23	23 (100.0)	10 (43.5)	13 (56.5)	2009-2011
	Total	31976291	123	123 (100.0)	59 (48.0)	64 (52.0)	2003-2011
**North-east**	Adamawa	4820641	21	21 (100.0)	3 (14.3)	18 (85.7)	2008-2010
	Bauchi	7115669	20	20 (100.0)	9 (45.0)	11 (55.0)	2008-2013
	Borno	6316532	27	27 (100.0)	13 (48.1)	14 (51.9)	2008-2018
	Gombe	3581705	11	11 (100.0)	1 (9.1)	10 (90.9)	2008-2009
	Taraba	3470026	16	16 (100.0)	3 (18.8)	13 (81.3)	2008-2010
	Yobe	3532576	17	17 (100.0)	6 (35.3)	11 (64.7)	2008-2010
	Total	28837149	112	112 (100.0)	35 (31.3)	77 (68.8)	2008-2018
**North-west**	Jigawa	6616983	27	27 (100.0)	0 (0.0)	27 (100.0)	2008-2010
	Kaduna	9230991	23	23 (100.0)	5 (21.7)	18 (78.3)	2008-2011
	Kano	14278381	44	44 (100.0)	0 (0.0)	44 (100.0)	2008-2010
	Katsina	8814090	34	34 (100.0)	4 (11.8)	30 (88.2)	2008-2013
	Kebbi	4927956	21	21 (100.0)	1 (4.8)	20 (95.2)	2008-2010
	Sokoto	5625422	23	23 (100.0)	3 (13.0)	20 (87.0)	2008-2011
	Zamfara	4960241	14	14 (100.0)	0 (0.0)	14 (100.0)	2008-2010
	Total	54454064	186	186(100.0)	13 (7.0)	173 (93.0)	2008-2013
**North-central**	Benue	6420080	23	23 (100.0)	7 (30.4)	16 (69.6)	2004-2013
	FCT, Abuja	2138179	6	6 (100.0)	2 (33.3)	4 (66.7)	2008-2010
	Kogi	4988605	21	21 (100.0)	10 (47.6)	11 (52.4)	2008-2010
	Kwara	3607893	16	16 (100.0)	5 (31.3)	11 (68.8)	2003-2010
	Nasarawa	2835193	13	13 (100.0)	0 (0.0)	13 (100.0)	2000
	Niger	6010772	25	25 (100.0)	6 (24.0)	19 (76.0)	2008-2011
	Plateau	4836786	17	17 (100.0)	0 (0.0)	17 (100.0)	2000
	Total	30837508	121	121(100.0)	30 (24.8)	91 (75.2)	2000-2013
	**Total**	**213000099**	**774**	**774 (100.0)**	**191 (24.7)**	**583 (75.3)**	**2000-2011**

LGA: Local Government Areas.

Overall, Nigeria achieved complete mapping coverage of all 774 LGAs, representing the entire national population of over 213 million people. Of these, 583 (75.3%) were endemic for LF (Fig 1). Endemicity varied across the six geopolitical zones in the country (Table 1), with the heaviest burden (>90% of all IUs) in the North-west and South-east zones, while the South-south had the lowest prevalence at 52%. In the South-east zone, 95 LGAs across the five states were mapped between 2005 and 2011, with an overall endemicity of 92.6%. Three states in the zone (Abia, Anambra, and Imo) had 100% endemicity. In the South-west, 137 LGAs were mapped across six states between 2003–2013, and 65.7% were endemic. Endemicity ranged between 30–94%, with the lowest in Lagos and Oyo. In the South-south, mapping was completed between 2003 and 2011 for 123 LGAs, with overall endemicity of 52.0%. Bayelsa recorded the highest endemicity with 87.5% of LGAs affected, while Akwa Ibom and Edo showed relatively low levels, at 35.5% and 38.9%, respectively. In the North-east, 112 LGAs across six states were mapped between 2008 and 2018, with an average endemicity of 68.8%. Gombe had the highest burden (90.9%), followed by Adamawa (85.7%) and Taraba (81.3%). In the North-west, 186 LGAs were mapped across seven states between 2008 and 2013, with high endemicity of 93.0%. All the LGAs in Kano and Jigawa were endemic. Katsina, Kebbi, Sokoto, Kaduna and Zamfara also had high endemicity exceeding 75%. In the North-central zone, 121 LGAs were mapped between 2000 and 2013, with 75.2% found to be endemic. All the LGAs in Nasarawa and Plateau were endemic. Benue and Niger had two-third of their LGAs endemic, while Kogi, FCT Abuja and Kwara had over half of their LGAs endemic (Table 1).

**Fig 1:**
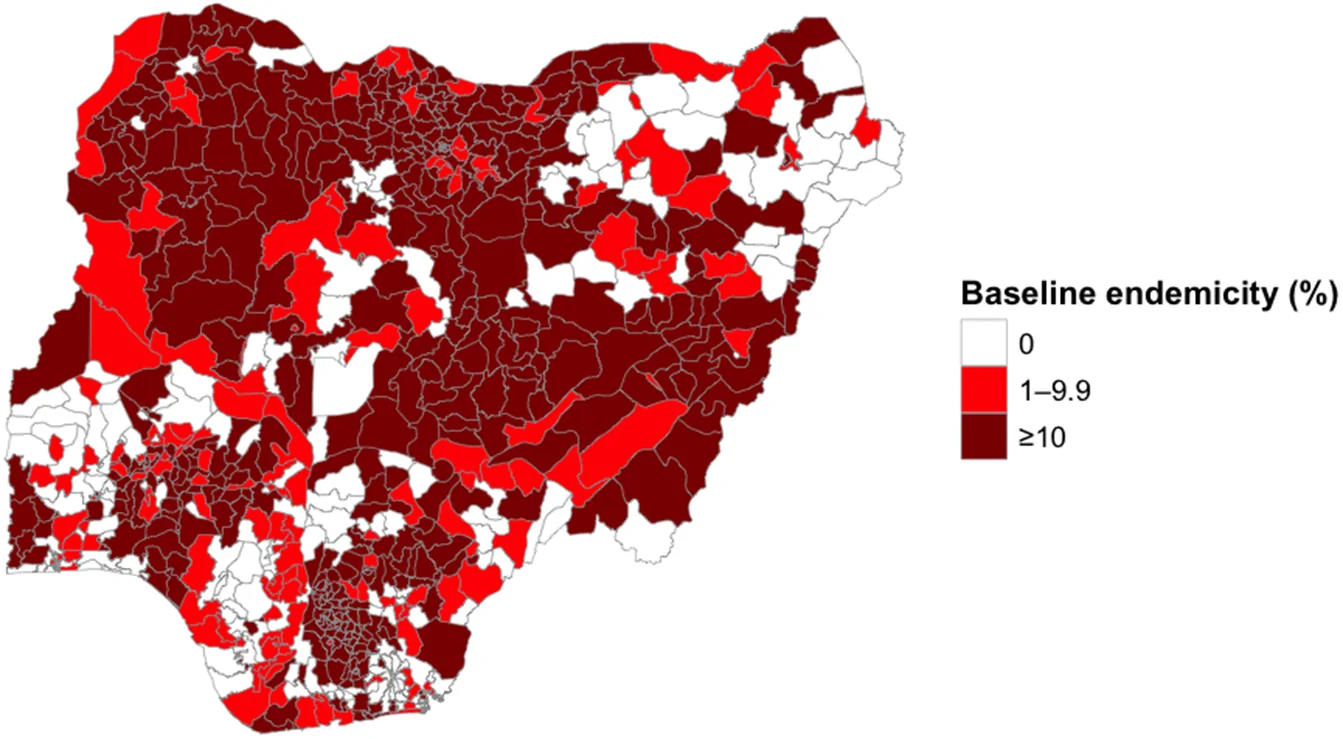
Map of Nigeria showing the Baseline endemicity of Lymphatic Filariasis prior to implementation of MAM. This map was created using programme primary data (See S1 Data) and developed in R software (v4.3.3). Shapefiles for Nigeria, including boundary polygons and water bodies across all administrative levels, were sourced from publicly accessible databases on the Humanitarian Data Exchange and are licensed under CC BY 4.0 (https://data.humdata.org/dataset/cod-ab-nga). No changes were made to the original shapefiles. The authors grant permission for the reuse of this map without restriction, provided appropriate credit is given to the source.

### Mass administration of medicines campaigns for LF control in Nigeria

Following the delineation of endemic LGAs, the next and most resource-intensive phase is mass administration of medicines (MAM), which involves the periodic delivery of antifilarial medicines (ivermectin and albendazole) to at-risk populations within endemic LGAs, now referred to as IUs [1,2,11]. Each MAM is implemented at the community level and targets all eligible individuals aged ≥5 years, using community-directed distributors (CDDs) who are unpaid volunteers selected by their communities. A typical MAM “round” represents one complete treatment cycle, conducted once yearly [11]. It begins with the training of CDDs, followed by advocacy visits and community sensitization to ensure awareness and acceptance. These preliminary steps are essential for achieving high treatment coverage and maintaining program fidelity. During drug distribution, CDDs record treatments in village registers, which are then collated and submitted to LGA-level NTD coordinators. National and global guidelines recommend achieving absolute geographic coverage, i.e., reaching all households and attaining a minimum epidemiological and programmatic coverage thresholds, generally at least 65% of the total population or 80% of the eligible population to sufficiently reduce microfilaria transmission [11]. Sustaining these levels for a minimum of five MAM rounds is expected to achieve a ∼99% reduction in prevalence required to interrupt transmission, and only LGAs that meet these requirements are considered eligible for epidemiological assessments to verify whether transmission has been interrupted. At the national level, all 583 IUs across the country have been reached with MAM since initiation between 2000 and 2015 [13]. By the time of this publication, the number of rounds administered ranged from 3 to 15, and 529 IUs (90.7%) have achieved the WHO-recommended ≥5 effective rounds. The South-east and North-west zones demonstrate exemplary performance with about 9 effective MAM rounds, while gaps remain in the North-east and parts of the South-south and South-west (Fig 2).

**Fig 2:**
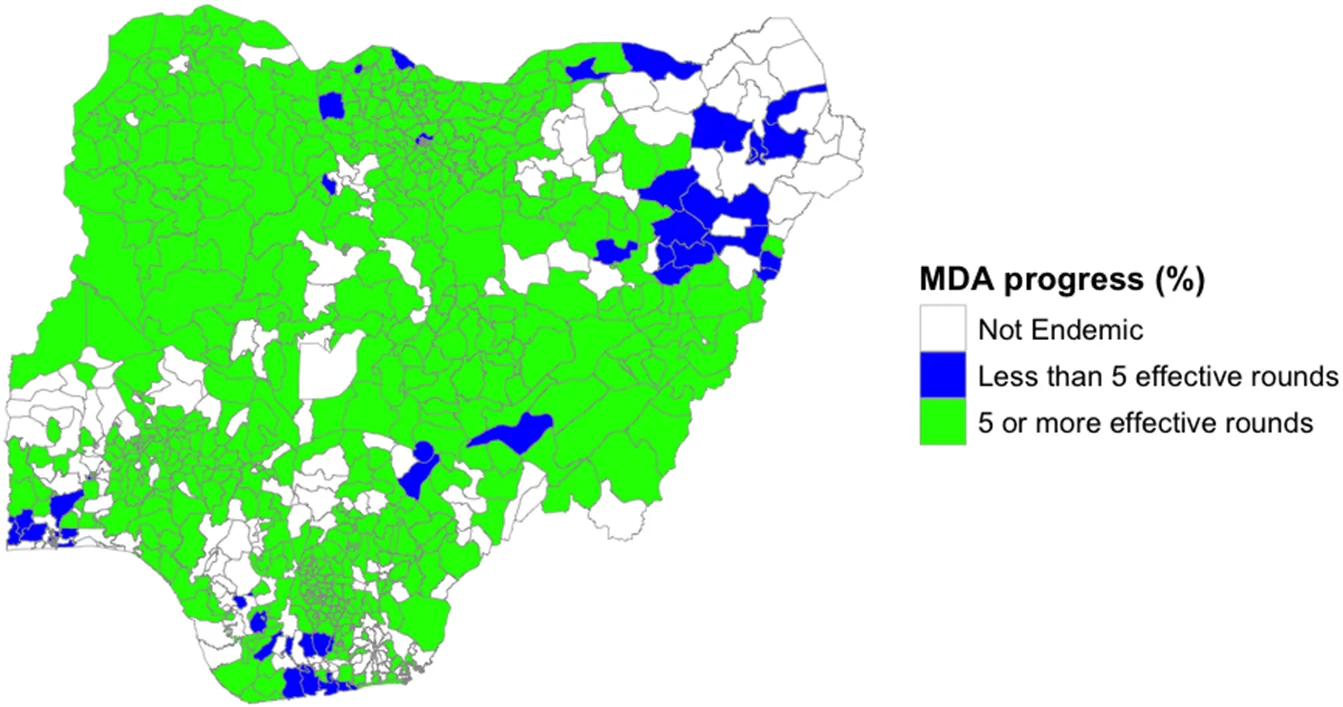
Map of Nigeria showing the progress of MAM for Lymphatic Filariasis Control as of 2024. This map was created using programme primary data (See S1 Data) and developed in R software v4.3.3 software. Shapefiles for Nigeria, including boundary polygons and water bodies across all administrative levels, were sourced from publicly accessible databases available at Humanitarian Data Exchange, and are licensed under CC BY 4.0 (https://data.humdata.org/dataset/cod-ab-nga). No changes were made to the original shapefiles. The authors grant permission for the reuse of this map without restriction, provided appropriate credit is given to the source.

The progress of MAM varied across the six-geopolitical regions in the country. In the South-east, all 88 IUs have already achieved at least five effective MAM rounds. This marks the South-east as one of the strongest performing zones nationally. In the South-west, the number of effective MAM rounds varied from as few as 3 rounds in Ogun to as many as 15 rounds in Osun. About 87% of IUs have had ≥ 5 effective MAM, mostly from Ekiti, Ondo, Osun, and Oyo. However, challenges remain in Ogun and Lagos. In the South-south, all 64 IUs were reached with MAM between 2010 and 2015. While most states have all IUs meeting the effective threshold, Rivers State stand out as a concern, with 84.6% of their IUs still below 5 effective rounds. In the North-east, MAM was initiated between 2009 and 2019 across all IUs (Table 2).

**Table 2:** Lymphatic Filariasis MAM progress in Nigeria as of 2024.

Zone	State	Number of endemic LGAs	LGAs experiencing MAM (i.e., IUs)	Year of Initiation	MAM rounds completed	IUs with Effective MAM rounds < 5 (%)	IUs with Effective MAM rounds ≥5 (%)
**South-east**	Abia	17	17 (100.0)	2014	7-8	0 (0.0)	17 (100.0)
	Anambra	21	21 (100.0)	2014	6-8	0 (0.0)	21 (100.0)
	Ebonyi	9	9 (100.0)	2014	6-9	0 (0.0)	9 (100.0)
	Enugu	14	14 (100.0)	2014	7-9	0 (0.0)	14 (100.0)
	Imo	27	27 (100.0)	2014	6-8	0 (0.0)	27 (100.0)
	Total	88	88 (100.0)	2014	6-9	0 (0.0)	88 (100.0)
**South-west**	Ekiti	16	16 (100.0)	2008	5-13	0 (0.0)	16 (100.0)
	Lagos	6	6 (100.0)	2020	11-14	6 (100.0)	0 (0.0)
	Ogun	14	14 (100.0)	2014	3	4 (28.6)	10 (71.4)
	Ondo	17	17 (100.0)	2008	6-9	0 (0.0)	17 (100.0)
	Osun	27	27 (100.0)	2008	10-15	0 (0.0)	27 (100.0)
	Oyo	10	10 (100.0)	2015	5-13	2 (20.0)	8 (80.0)
	Total	90	90 (100.0)	2008-2015	3-15	12 (13.3)	78 (86.7)
**South-south**	Akwa Ibom	11	11 (100.0)	2014	7-8	0 (0.0)	11 (100.0)
	Bayelsa	7	7 (100.0)	2011	4-7	1 (14.3)	6 (85.7)
	Cross River	10	10 (100.0)	2010	8-12	0 (0.0)	10 (100.0)
	Delta	16	16 (100.0)	2014	7-9	2 (12.5)	14 (87.5)
	Edo	7	7 (100.0)	2014	7-10	0 (0.0)	7 (100.0)
	Rivers	13	13 (100.0)	2015	4-7	11 (84.6)	2 (15.4)
	Total	64	64 (100.0)	2010-2015	4-12	14 (21.9)	50 (78.1)
**North-east**	Adamawa	18	18 (100.0)	2012	4-9	2 (11.1)	16 (88.9)
	Bauchi	11	11 (100.0)	2012	7-11	0 (0.0)	11 (100.0)
	Borno	14	14 (100.0)	2019	1-4	14 (100.0)	0 (0.0)
	Gombe	10	10 (100.0)	2009	7-9	1 (10.0)	9 (90.0)
	Taraba	13	13 (100.0)	2009	8-13	1 (7.7)	12 (92.3)
	Yobe	11	11 (100.0)	2011	10-11	3 (27.3)	8 (72.7)
	Total	77	77 (100.0)	2009-2012	1-13	21 (27.3)	56 (72.7)
**North-west**	Jigawa	27	27 (100.0)	2011	8-11	0 (0.0)	27 (100.0)
	Kaduna	18	18 (100.0)	2010	6-12	0 (0.0)	18 (100.0)
	Kano	44	44 (100.0)	2010	7-11	1 (2.3)	43 (97.7)
	Katsina	30	30 (100.0)	2015	7-11	4 (13.3)	26 (86.7)
	Kebbi	20	20 (100.0)	2010	7-8	0 (0.0)	20 (100.0)
	Sokoto	20	20 (100.0)	2011	6-14	0 (0.0)	20 (100.0)
	Zamfara	14	14 (100.0)	2010	6-13	0 (0.0)	14 (100.0)
	Total	173	173 (100.0)	2010-2015	6-14	5 (2.9)	168 (97.1)
**North-central**	Benue	16	16 (100.0)	2012	6-9	2 (12.5)	14 (87.5)
	FCT, Abuja	4	4 (100.0)	2010	9-11	0 (0.0)	4 (100.0)
	Kogi	11	11 (100.0)	2010	5-11	0 (0.0)	11 (100.0)
	Kwara	11	11 (100.0)	2010	5-11	0 (0.0)	11 (100.0)
	Nasarawa	13	13 (100.0)	2000	8-13	0 (0.0)	13 (100.0)
	Niger	19	19 (100.0)	2000	8-13	0 (0.0)	19 (100.0)
	Plateau	17	17 (100.0)	2011	7-13	0 (0.0)	17 (100.0)
	Total	91	91 (100.0)	2000-2012	5-13	2 (2.2)	89 (97.8)
**Total**		583	583 (100.0)	2000-2015	3-15	54 (9.3)	529 (90.7)

LGA: Local Government Areas; IUs: Implementation Units.

The implementation history reveals considerable variation, with states like Yobe and Taraba implementing between 10 and 13 rounds in most LGAs, Borno only started MAM in 2019, with none of its IUs meeting the required ≥5 effective rounds of MAM. Hence only 72.7% of IUs in the zone have achieved ≥5 effective rounds. In the North-west zone, nearly all IUs (97.1%) have achieved at least five effective rounds, except in Kano and Katsina. This zone stands out as one of the most successful in terms of scale and effectiveness of MAM implementation. In North-central, all 91 IUs across seven states were reached with MAM beginning as early as 2000 in Nasarawa and Niger, and later in others like Benue and Plateau. The number of treatment rounds ranged from 5 to 13. Overall, 95.6% of IUs have completed ≥5 effective rounds. The Federal Capital Territory (FCT) was somewhat unique, with half of its IUs still under 5 rounds despite an early start in 2010. Otherwise, most states in this zone have shown consistent progress, placing North-central among the stronger performing zones (Table 2).

### Epidemiological assessments to ascertain LF transmission status in Nigeria

Following mapping and the effective implementation of at least five rounds of MAM with ivermectin and albendazole in endemic areas, IUs are required to demonstrate that infection levels have been reduced to the point where transmission is unlikely, i.e., < 2% for antigenemia or <1% for microfilariae (Fig 3). These assessments are coordinated by the NLFEP and involve four sequential stages (EMS, TAS-1, TAS-2, TAS-3) conducted within evaluation units (EUs). An EU is a programmatically defined geographic area comprising one or more contiguous IUs, typically with similar epidemiological characteristics and treatment histories, and a population size within the WHO-recommended threshold (generally not exceeding 2 million) [11]. The EUs are formed to ensure feasibility of assessment and the resulting outcomes are applied to, and inherited by, all IUs within the EU for programmatic decision-making [11].

**Fig 3:**
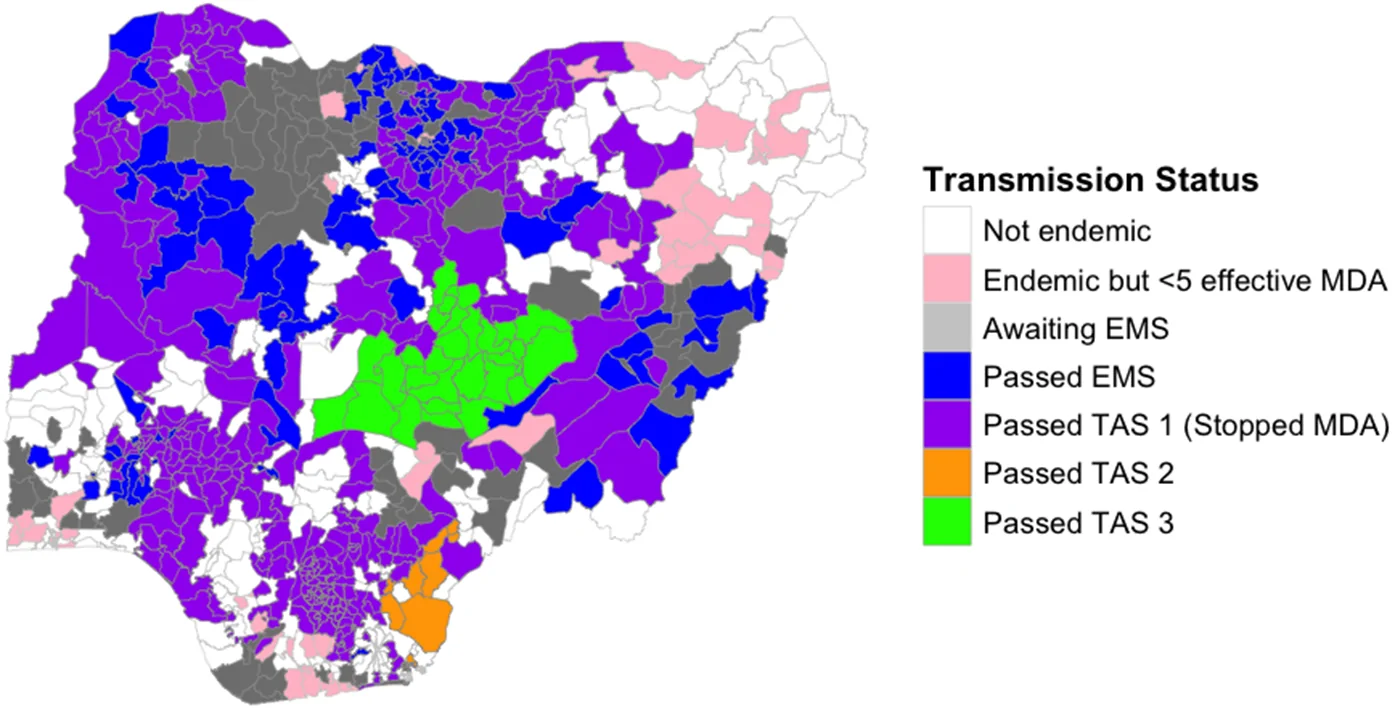
Map of Nigeria showing the transmission status of Lymphatic Filariasis as of 2024. This map was created using primary data collected by the authors (S1 Data) and developed in R software v4.3.3 software. Shapefiles for Nigeria, including boundary polygons and water bodies across all administrative levels, were sourced from publicly accessible databases available at Humanitarian Data Exchange, and are licensed under CC BY 4.0 (https://data.humdata.org/dataset/cod-ab-nga). No changes were made to the original shapefiles. The authors grant permission for the reuse of this map without restriction, provided appropriate credit is given to the source.

The first assessment is the pre-transmission assessment survey (pre-TAS), also referred to as the EMS, which is conducted to determine whether infection levels have declined sufficiently for an EU to proceed to TAS-1 [11].. During the EMS, parasitological and serological assessments are typically conducted among children aged 5 years and above at two sites: one sentinel site established and surveyed before the initiation of MAM and one additional spot-check site [27]. However, the revised WHO guidelines now stipulate that eligibility should be assessed among individuals aged 19 years and older [28]. An EU qualifies for first TAS (i.e., TAS-1) if seroprevalence is below 1% for microfilaria or below 2% for antigenemia. If these thresholds are not met, the EU is advised to implement two additional rounds of effective MAM before repeating the EMS [28].

In the second step, EUs that pass EMS advance to the TAS-1, which is conducted among children aged 6–7 years and determines eligibility to stop MAM. Under previous guidance, EUs were judged to be successful if seroprevalence was < 2% for antigenemia or <1% for microfilariae; however, the revised guidance requires <1% for both. In this report, TAS-1 was conducted before the updated criteria and evaluated according to the earlier thresholds. The EUs that fail TAS-1 are required to undergo two additional rounds of effective MAM before repeating the evaluation, while those that pass are allowed to stop MAM and enter the post-treatment surveillance (PTS) phase [22]. The PTS phase comprises of two subsequent surveys (TAS-2 and TAS-3), conducted at intervals of 2–3 years after stopping MAM, and used to assess whether interruption of LF transmission has been achieved (TAS-2) and sustained over time (TAS-3) [11]. Successful passage through these stages allows the country to progress to the validation phase, where all EUs must have passed TAS-3 to be considered eligible for validation of LF transmission interruption.

Of the 583 IUs, 529 (90.7%) have completed at least five effective rounds of MAM, representing substantial progress toward eligibility for EMS (Fig 3). Overall, 445 IUs (76.3%) have passed EMS and 84 (14.4%) are awaiting EMS. Furthermore, 346 (59.3%) have successfully passed TAS-1 and stopped MAM. Progression beyond TAS-1 remains limited nationally, with only 40 IUs (6.9%) having passed TAS-2 and 30 IUs (5.1%) having passed TAS-3 (Table 3).

**Table 3:** Transmission Status of Lymphatic Filariasis in Nigeria as of 2024.

Zone	State	No. of IUs	No. of IUs with at least 5 effective MAM (%)	No. of IUs that has passed EMS (%)	No. of IUs that has passed TAS-1 (%)	No. of IUs that has passed TAS-2 (%)	No. of IUs that has passed TAS-3 (%)
**South-east**	Abia	17	17 (100.0)	17 (100.0)	17 (100.0)	0 (0.0)	0 (0.0)
	Anambra	21	21 (100.0)	21 (100.0)	21 (100.0)	0 (0.0)	0 (0.0)
	Ebonyi	9	9 (100.0)	9 (100.0)	9 (100.0)	0 (0.0)	0 (0.0)
	Enugu	14	14 (100.0)	14 (100.0)	14 (100.0)	0 (0.0)	0 (0.0)
	Imo	27	27 (100.0)	27 (100.0)	23 (85.2)	0 (0.0)	0 (0.0)
	Total	88	88 (100.0)	88 (100.0)	84 (95.5)	0 (0.0)	0 (0.0)
**South-west**	Ekiti	16	16 (100.0)	16 (100.0)	16 (100.0)	0 (0.0)	0 (0.0)
	Lagos	6	0 (0.0)	0 (0.0)	0 (0.0)	0 (0.0)	0 (0.0)
	Ogun	14	10 (71.4)	0 (0.0)	0 (0.0)	0 (0.0)	0 (0.0)
	Ondo	17	17 (100.0)	17 (100.0)	17 (100.0)	0 (0.0)	0 (0.0)
	Osun	27	27 (100.0)	27 (100.0)	15 (55.6)	0 (0.0)	0 (0.0)
	Oyo	10	8 (80.0)	6 (60.0)	3 (30.0)	0 (0.0)	0 (0.0)
	Total	90	78 (86.7)	66 (73.3)	51 (56.7)	0 (0.0)	0 (0.0)
**South-south**	Akwa Ibom	11	11 (100.0)	7 (63.6)	6 (54.5)	0 (0.0)	0 (0.0)
	Bayelsa	7	6 (85.7)	1 (14.3)	1 (14.3)	0 (0.0)	0 (0.0)
	Cross River	10	10 (100.0)	10 (100.0)	10 (100.0)	8 (80.0)	0 (0.0)
	Delta	16	14 (87.5)	14 (87.5)	14 (87.5)	0 (0.0)	0 (0.0)
	Edo	7	7 (100.0)	7 (100.0)	7 (100.0)	0 (0.0)	0 (0.0)
	Rivers	13	2 (15.4)	0 (0.0)	0 (0.0)	0 (0.0)	0 (0.0)
	Total	64	50 (78.1)	39 (60.9)	38 (59.4)	8 (12.5)	0 (0.0)
**North-east**	Adamawa	18	16 (88.9)	7 (38.9)	0 (0.0)	0 (0.0)	0 (0.0)
	Bauchi	11	11 (100.0)	9 (81.8)	7 (63.6)	0 (0.0)	0 (0.0)
	Borno	14	0 (0.0)	0 (0.0)	0 (0.0)	0 (0.0)	0 (0.0)
	Gombe	10	9 (90.0)	9 (90.0)	8 (80.0)	0 (0.0)	0 (0.0)
	Taraba	13	12 (92.3)	10 (76.9)	5 (38.5)	0 (0.0)	0 (0.0)
	Yobe	11	8 (72.7)	8 (72.7)	8 (72.7)	0 (0.0)	0 (0.0)
	Total	77	56 (72.7)	43 (55.8)	28 (36.4)	0 (0.0)	0 (0.0)
**North-west**	Jigawa	27	27 (100.0)	24 (88.9)	21 (77.8)	0 (0.0)	0 (0.0)
	Kaduna	18	18 (100.0)	15 (83.3)	9 (50.0)	0 (0.0)	0 (0.0)
	Kano	44	43 (97.7)	40 (90.9)	17 (38.6)	0 (0.0)	0 (0.0)
	Katsina	30	26 (86.7)	14 (46.7)	0 (0.0)	0 (0.0)	0 (0.0)
	Kebbi	20	20 (100.0)	20 (100.0)	14 (70.0)	0 (0.0)	0 (0.0)
	Sokoto	20	20 (100.0)	18 (90.0)	16 (80.0)	0 (0.0)	0 (0.0)
	Zamfara	14	14 (100.0)	0 (0.0)	0 (0.0)	0 (0.0)	0 (0.0)
	Total	173	168 (97.1)	131 (75.7)	77 (44.5)	0 (0.0)	0 (0.0)
**North-central**	Benue	16	14 (87.5)	3 (18.8)	3 (18.8)	0 (0.0)	0 (0.0)
	FCT, Abuja	4	4 (100.0)	4 (100.0)	2 (50.0)	2 (50.0)	0 (0.0)
	Kogi	11	11 (100.0)	11 (100.0)	9 (81.8)	0 (0.0)	0 (0.0)
	Kwara	11	11 (100.0)	11 (100.0)	10 (90.9)	0 (0.0)	0 (0.0)
	Nasarawa	13	13 (100.0)	13 (100.0)	13 (100.0)	13 (100.0)	13 (100.0)
	Niger	19	19 (100.0)	19 (100.0)	10 (52.6)	0 (0.0)	0 (0.0)
	Plateau	17	17 (100.0)	17 (100.0)	17 (100.0)	17 (100.0)	17 (100.0)
	Total	91	89 (97.8)	78 (85.7)	64 (70.3)	32 (35.2)	30 (33.0)
							
**Total**		583	529 (90.7)	443 (76.3)	346 (59.3)	40 (6.9)	30 (5.1)

IUs: Implementation Units; EMS: Epidemiological Monitoring Survey; MAM: Mass Administration of Medicines; TAS: Transmission Assessment Surveys; Percentages are calculated using the number of IUs as the denominator; Eligibility-based activities such as EMS and TAS are conducted only in IUs that meet programmatic criteria (e.g., ≥ 5 effective MAM rounds) but are presented here to summarize overall progress among endemic EUs.

Marked geographical variation in transmission status was observed across the six geopolitical zones. In the South-east zone, all 88 IUs completed at least five effective MAM rounds and passed EMS (100%), while 84 (95.5%) passed TAS-1 and stopped MAM, indicating strong programmatic performance in the zone. These IUs are currently awaiting progression to TAS-2. In the South-west zone, 78 of 90 IUs (86.7%) completed at least five effective MAM rounds, 66 IUs (73.3%) passed EMS, and 51 IUs (56.7%) passed TAS-1 and stopped MAM. However, substantial gaps remain, particularly in Lagos State, where none of the IUs had progressed to EMS, and in Ogun State, where 10 IUs (71.4%) had completed effective MAM but were yet to undergo EMS.

In the South-south zone, 50 of 64 IUs (78.1%) completed at least five effective MAM rounds, 39 (60.9%) passed EMS, and 38 (59.4%) passed TAS-1, while 8 (12.5%) progressed to TAS-2. Performance varied widely within the zone, with Rivers and Bayelsa States exhibiting substantial challenges related to low MAM coverage and limited advancement through assessment surveys. In the North-east zone, 56 of 77 IUs (72.7%) completed at least five effective MAM rounds, 43 (55.8%) passed EMS, and 28 (36.4%) passed TAS-1, with none yet progressing to TAS-2. Similarly, in the North-west zone, although 168 of 173 IUs (97.1%) completed at least five effective MAM rounds, only 131 IUs (75.7%) passed EMS and 77 IUs (44.5%) passed TAS-1, highlighting slower progression toward transmission interruption despite high MAM coverage.

The North-central zone demonstrated the most advanced progress nationally. Of the 91 endemic IUs, 89 (97.8%) completed at least five effective MAM rounds, 78 IUs (85.7%) passed EMS, and 64 IUs (70.3%) passed TAS-1. Notably, 32 IUs (35.2%) passed TAS-2 and 30 IUs (33.0%) passed TAS-3, confirming sustained interruption of transmission in a substantial proportion of IUs in the zone. States such as Nasarawa and Plateau achieved complete progression through TAS-3, reflecting exceptional programmatic performance and serving as potential models for other endemic areas (Table 3).

### Profile of states with limited progress in the LF elimination program in each zone

The least-performing states within each geopolitical zone were identified and profiled. Overall, progress toward LF elimination remained limited in six states: Katsina, Zamfara, Borno, Rivers, Ogun, and Lagos. (Table 4). Katsina had relatively strong programmatic performance, with over 85% of endemic IUs completing effective MAM and approximately 47% progressing to and passing EMS. Zamfara achieved full completion of effective MAM across endemic IUs; however, none had progressed to EMS (Table 4). None of the IUs in Borno and Lagos have had 5 effective MAM rounds. Ogun and Rivers State showed moderate MAM completion but had not advanced to EMS (Table 4). Programme records indicated that progress in the northern states was affected by persistent insecurity, while implementation challenges in parts of the southern states were associated with funding constraints, limited partner support, and reduced donor engagement (Table 4).

**Table 4:** Profile of States with limited progress in the LF Elimination program.

State	Number of IUs	Number of IUs with effective MAM completed (%)	Number of IUs that have passed EMS (%)	Challenges
Katsina	30	26(86.7)	14(47)	Insecurity
Zamfara	14	14 (100)	0(0)	Insecurity
Borno	14	0(0)	0(0)	Insecurity
Rivers	13	2(15.4)	0(0)	Insecurity
Ogun	14	10(71.4)	0(0)	Funding, lack of partners and donors
Lagos	6	0(0)	0(0)	Funding

IUs: Implementation Units; MAM: Mass Administration of Medicines; EMS: Epidemiological Monitoring Survey; Insecurity refers to security challenges associated with terrorism, banditry, and communal clashes.

### Planned transmission assessment surveys in Nigeria (2024–2027)

At the national level, a total of 499 IUs are expected to undergo different assessment between 2024 and 2027 as part of the elimination strategy. In 2024, 135 IUs (27.2%) were expected to undergo either EMS or TAS, increasing to 173 IUs (34.8%) in 2025, 123 IUs (24.6%) in 2026 and 68 IUs (13.6%) in 2027 (Table 5).

**Table 5:** Planned assessment in Nigeria (2024–2027).

		Number of IUs				
Zone	Assessment Stage	Overall Total (%)	No. in Year 2024 (%)	No. in Year 2025 (%)	No. in Year 2026 (%)	No. in Year 2027 (%)
**South-east**	EMS	0(0)	0(0)	0(0)	0(0)	0(0)
	TAS-1	4 (4.5)	0 (0.0)	0 (0.0)	4 (100.0)	0 (0.0)
	TAS-2	84 (95.5)	15 (17.9)	45 (53.6)	13 (15.5)	11 (13.1)
	TAS-3	0(0)	0(0)	0(0)	0(0)	0(0)
	Total	88 (100.0)	15 (17.0)	45 (51.1)	17 (19.3)	11 (12.5)
**South-west**	EMS	12 (15.4)	12 (100.0)	0 (0.0)	0 (0.0)	0 (0.0)
	TAS-1	17 (21.8)	2 (11.8)	15 (88.2)	0 (0.0)	0 (0.0)
	TAS-2	49 (62.8)	0 (0.0)	10 (20.4)	22 (44.9)	17 (34.7)
	TAS-3	0(0)	0(0)	0(0)	0(0)	0(0)
	Total	78 (100.0)	14 (18.0)	25 (32.1)	22 (28.2)	17 (21.8)
**South-south**	EMS	11 (22.0)	7 (63.6)	4 (36.4)	0 (0.0)	0 (0.0)
	TAS-1	1 (2.0)	1 (100.0)	0 (0.0)	0 (0.0)	0 (0.0)
	TAS-2	30 (60.0)	1 (3.3)	1 (3.3)	15 (50.0)	13 (43.3)
	TAS-3	8 (16.0)	0 (0.0)	5 (62.5)	3 (37.5)	0 (0.0)
	Total	50(100.0)	9 (18.0)	10 (20.0)	18 (36.0)	13 (26.0)
**North-east**	EMS	14 (25.0)	1 (7.1)	13 (92.9)	0 (0.0)	0 (0.0)
	TAS-1	15 (26.8)	11 (73.3)	3 (20.0)	0 (0.0)	1 (6.7)
	TAS-2	27 (48.2)	0 (0.0)	14 (51.9)	7 (25.9)	6 (22.2)
	TAS-3	0 (0)	0 (0)	0 (0)	0 (0)	0 (0)
	Total	56 (100.0)	12 (21.4)	30 (53.6)	7 (12.5)	7 (12.5)
**North-west**	EMS	39 (23.2)	28 (75.7)	11 (24.3)	0 (0.0)	0 (0.0)
	TAS-1	52 (30.9)	36 (69.2)	16 (30.8)	0 (0.0)	0 (0.0)
	TAS-2	77 (45.8)	12 (15.6)	10 (13.0)	45 (58.4)	10 (13.0)
	TAS-3	0 (0)	0 (0)	0 (0)	0 (0)	0 (0)
	Total	168	76 (45.3)	37 (22.0)	45 (26.8)	10 (6.0)
**North-central**	EMS	12 (20.3)	3 (10.0)	9 (90.0)	0 (0.0)	0 (0.0)
	TAS-1	11 (18.6)	6 (55.5)	5 (45.5)	0 (0.0)	0 (0.0)
	TAS-2	34 (57.6)	0 (0.0)	12 (35.3)	12 (35.3)	10 (29.4)
	TAS-3	2 (3.4)	0 (0.0)	0 (0.0)	2 (100.0)	0 (0.0)
	Total	59 (100)	9 (15.8)	26 (45.6)	12 (21.1)	10 (17.5)
**Total**		499 (100)	135 (27.2)	173 (34.8)	123 (24.6)	68 (13.6)

IUs: Implementation Units; EMS: Epidemiological Monitoring Survey; MAM: Mass Administration of Medicines; TAS: Transmission Assessment Surveys.

In the South-east Zone, 88 IUs are planned for epidemiological assessments, with 84 (95.5%) scheduled for TAS-2 and 4 (4.5%) for TAS-1. The TAS-2 assessments are distributed across 15 IUs in 2024, 45 in 2025, 13 in 2026, and 11 in 2027. All four TAS-1 assessments are scheduled for 2026. (Table 5). Similarly, in the South-west zone, 78 IUs are scheduled for assessments between 2024 and 2027 including 12 (15.4%) of the IUs planning for EMS, 17 (21.8%) for TAS-1, and 49 (62.8%) for TAS-2. All EMS are scheduled for 2024, most TAS-1 assessments are planned for 2025, and TAS-2 assessments are distributed between 2025 and 2027 (Table 5). In the South-south zone, 50 IUs are scheduled for evaluation surveys between 2024 and 2027, including 11 (22.0%) IUs planned for EMS, 1(2.0%) for TAS-1, 30 (60.0%) for TAS-2, and 8 (16.0%) for TAS-3. EMS activities are scheduled for 2024 and 2025, TAS-1 for 2024, TAS-2 across all four years, and TAS-3 for 2025 and 2026 (Table 5).

In the North-east zone, 56 IUs are scheduled for evaluation surveys between 2024 and 2027, including 14 (25.0%) for EMS, 15 (26.8%) for TAS-1, and 27 (48.2%) for TAS-2. Most EMS assessments (92.9%) are planned for 2025, while TAS-1 assessments (93.3%) are scheduled for 2024 and 2025. TAS-2 assessments are distributed between 2025 and 2027 (Table 5).

In the North-west zone, 168 IUs are scheduled for evaluation surveys between 2024 and 2027, including 39 (23.2%) for EMS, 52 (30.9%) for TAS-1, and 77 (45.8%) for TAS-2. Most EMS activities (75.7%) are planned for 2024, while TAS-1 assessments are scheduled for 2024 (69.2%) and 2025 (30.8%). TAS-2 assessments are distributed across 2024–2027 (Table 5). Similarly, in the North-central zone, 57 IUs are scheduled for evaluation surveys between 2024 and 2027, including 12 (21.1%) for EMS, 11 (19.3%) for TAS-1, 32 (56.1%) for TAS-2, and 2 (3.6%) for TAS-3. EMS activities are primarily planned for 2025, TAS-1 assessments for 2024 (55.5%) and 2025, TAS-2 assessments for 2025–2027, and TAS-3 assessments for 2026 (Table 5).

### Historical failure rate for EMS across the country

Across the six geopolitical zones, only 18 (3.4%) of the 529 assessed IUs have ever failed EMS. Evidence of programme failure under these scenarios was limited geographically to only 11 of the 37 states. In the South-east zone, failures were observed in Anambra, Enugu, and Imo States (one LGAs each), and Ebonyi State (three LGAs). In the South-west, Ekiti and Osun States each had two affected IUs. In the South-south zone, Cross River State had one affected IU. In the North-west, Kebbi and Sokoto States each had two affected IUs, while in the North-central and North-east zones, Niger and Taraba States, respectively, each had two affected IUs (Fig 4).

**Fig 4:**
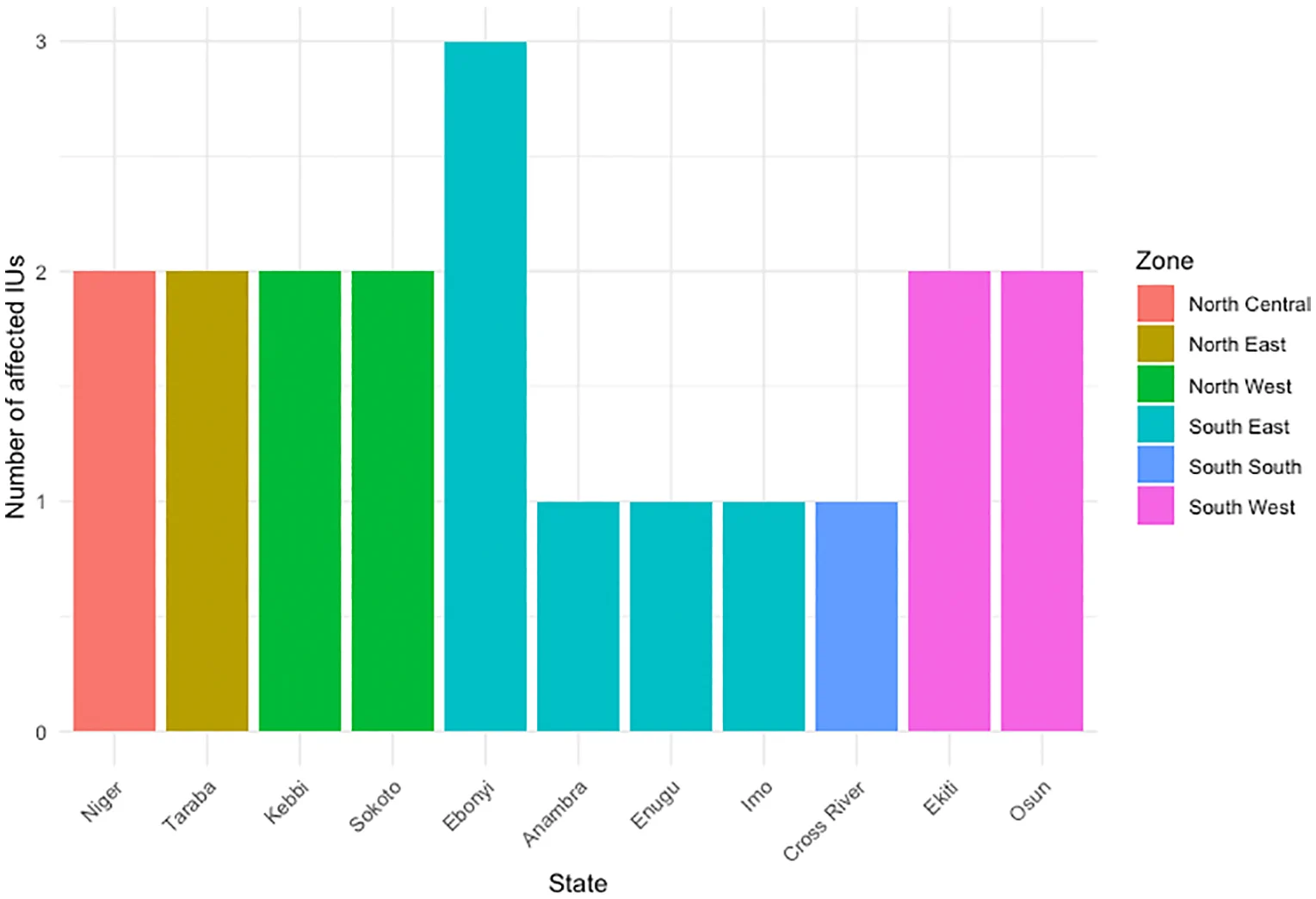
Number of Implementation Units (IUs) with at least one failed Epidemiological Monitoring Survey (EMS) by state from 2000–2025.

### Forecast for achieving 2030 Elimination Targets

Fig 5 illustrates the projected progression of IUs across assessment stages. Across all geopolitical zones, most IUs are projected to reach TAS-1 and subsequently progress to TAS-3 by or before the 2030 elimination endline, indicating that both cessation of MAM and achievement of elimination thresholds are programmatically feasible within the target timeline. Only a small subset of IUs in the Northern and south-south zone extends beyond 2030, which are attributable to later starting points rather than programmatic failure.

**Fig 5:**
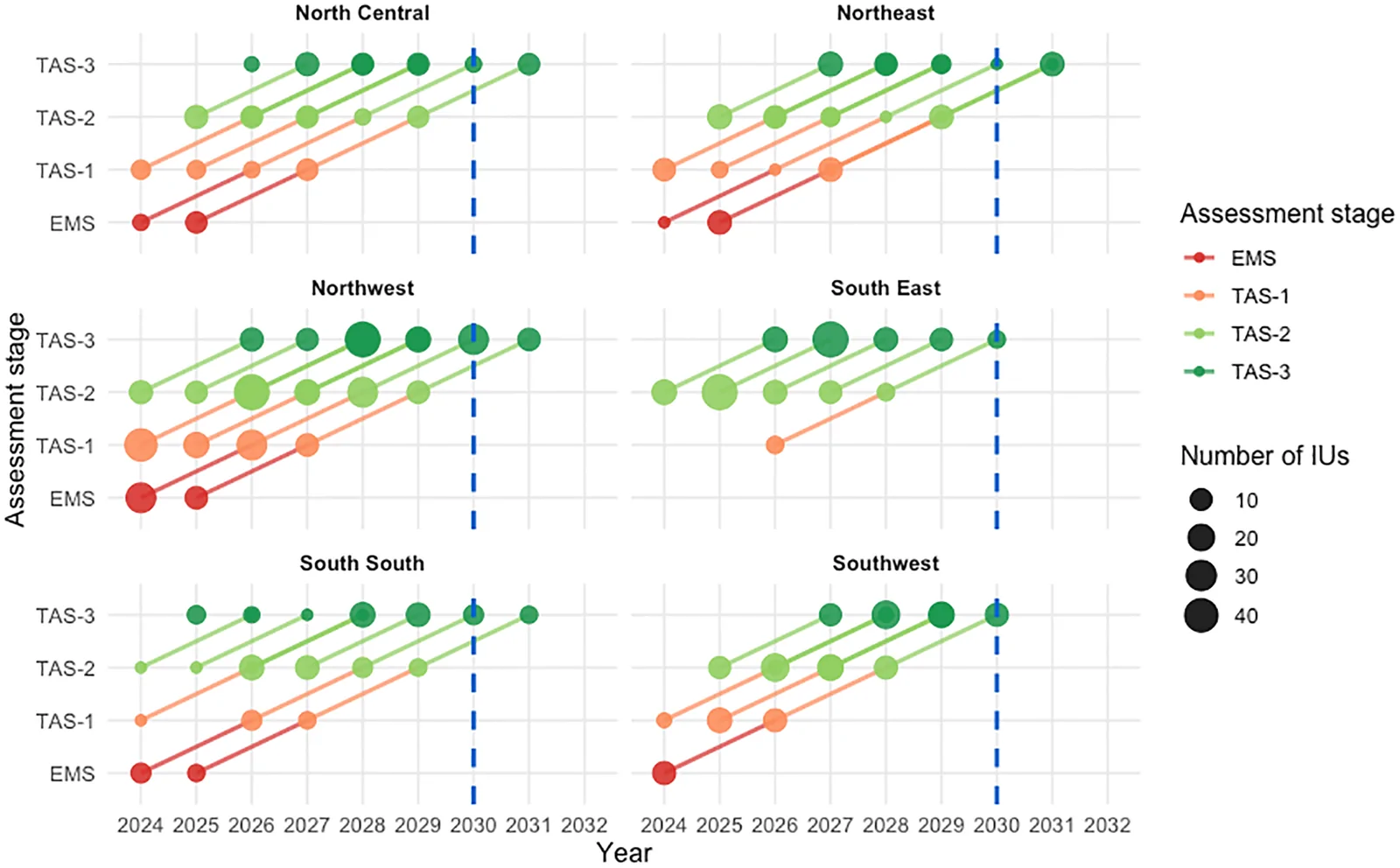
Projected progression of IUs across assessment stages across Nigeria.

By 2030, we project that MAM would have stopped in at least 497 IUs i.e., 85.2% of all IUs, and 309 (62.2%) of those would have passed TAS-3 (achieve elimination). However, under a conservative assumption that 3–5% of planned surveys can fail, these figures decline to 454 (77.9%) IUs stopping MAM and 284 achieving elimination (Fig 6). Considering the historical failure rate of 3.4% at the EMS stage, we applied hypothetical failure scenarios of 3% and 5% to represent potential failure or delayed progression across evaluation units. As illustrated in Fig 6, even under these more conservative assumptions, the projected number of evaluation units achieving cessation of MAM (TAS-1) and elimination (TAS-3) remains largely stable across all geopolitical zones, with only modest reductions (difference of 1–4 LGAs) observed as failure rates increase, indicating that programme targets remain attainable despite plausible operational setbacks.

**Fig 6:**
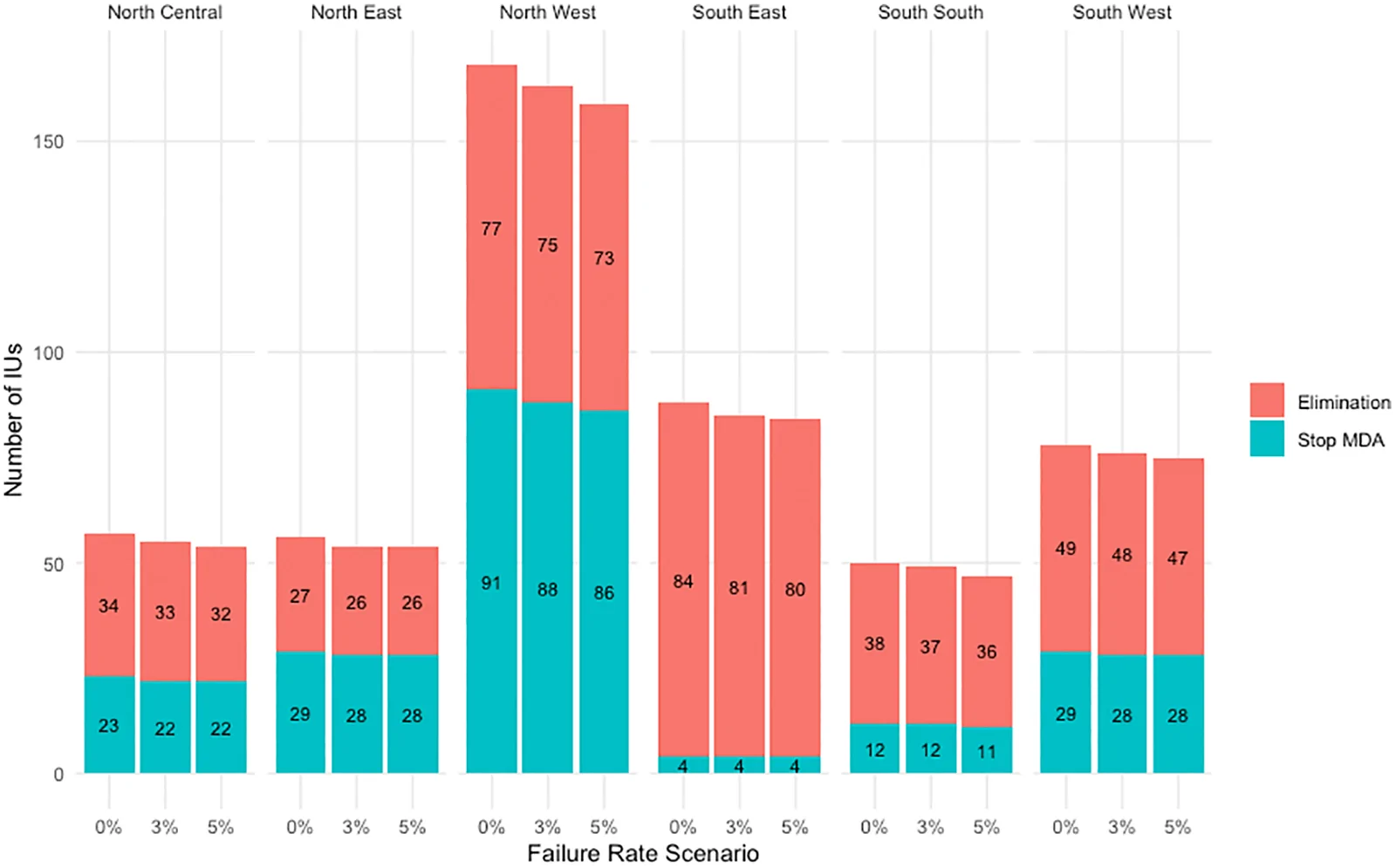
Forecast for achieving 2030 Targets (Stopping MAM and Passing TAS-3); Baseline projections for ‘Stop MDA’ and ‘Elimination’ were derived from the national planned assessment schedule, assuming all planned surveys are completed successfully with no failure.

### Morbidity management and disability prevention (MMDP) program

The GPELF recommends that all individuals affected by the chronic manifestations of LF have access to a comprehensive package of care. In parallel with MAM, the NLFEP implements MMDP services for individuals already affected by LF-related morbidity, particularly lymphoedema and hydrocele [1,11]. MMDP activities include identifying and documenting cases, training health workers on essential care packages for lymphoedema management, establishing routine reporting systems, and ensuring access to basic supplies for hygiene and acute attack management [5,10–12]. For hydrocele, the programme is expected to estimate surgical need, facilitate access to quality-assured hydrocelectomy services, and report on surgical outcomes [5,10–12]. These interventions not only alleviate symptoms but also prevent disease progression and reduce the frequency of debilitating episodes. To ensure sustainability, the package is expected to be integrated into primary healthcare (PHC) services, alongside measures that address stigma, promote mental health, and foster social inclusion for those affected [5].

### Health facility readiness assessment for MMDP services

As part of best practices before implementing MMDP activities, the WHO recommends conducting health facility assessments to evaluate the readiness of facilities to provide services in a safe and high-quality setting [29]. The MMDP service in Nigeria commenced with a situational analysis to understand the burden of LF related morbidities and the readiness of health facilities to provide minimum or basic essential care to identified patients in the country. These assessments were conducted conducted using the Hydrocele Facility Assessment (HFAT) and Lymphoedema Facility Assessment (LFAT) tools [30]. The inspection protocol was deployed on the Comcare application and administered by trained health workers. The tool has ten key components of care including background information of the facilities: surgical procedures, staff training, emergency patient transfer plans, infrastructure, essential medicines availability, disposable and non-disposable equipment, current hydrocele surgery practices, infection prevention and laboratory capacity to analyze samples during pre- and post-surgical investigations [30].

For HFAT, 154 (30.4%) of 508 facilities was assessed across 24 states, and 58 (37.7%) had a test score of 95% and above and were deemed qualified to conduct surgery (S2 Data, Fig 7). For LFAT, 716 facility was assessed across 212 LGAs, and 284 (39.7%) had a test score of 75% and above and were deemed ready to provide essential care for lymphoedema patients (S2 Data, Fig 8).

**Fig 7:**
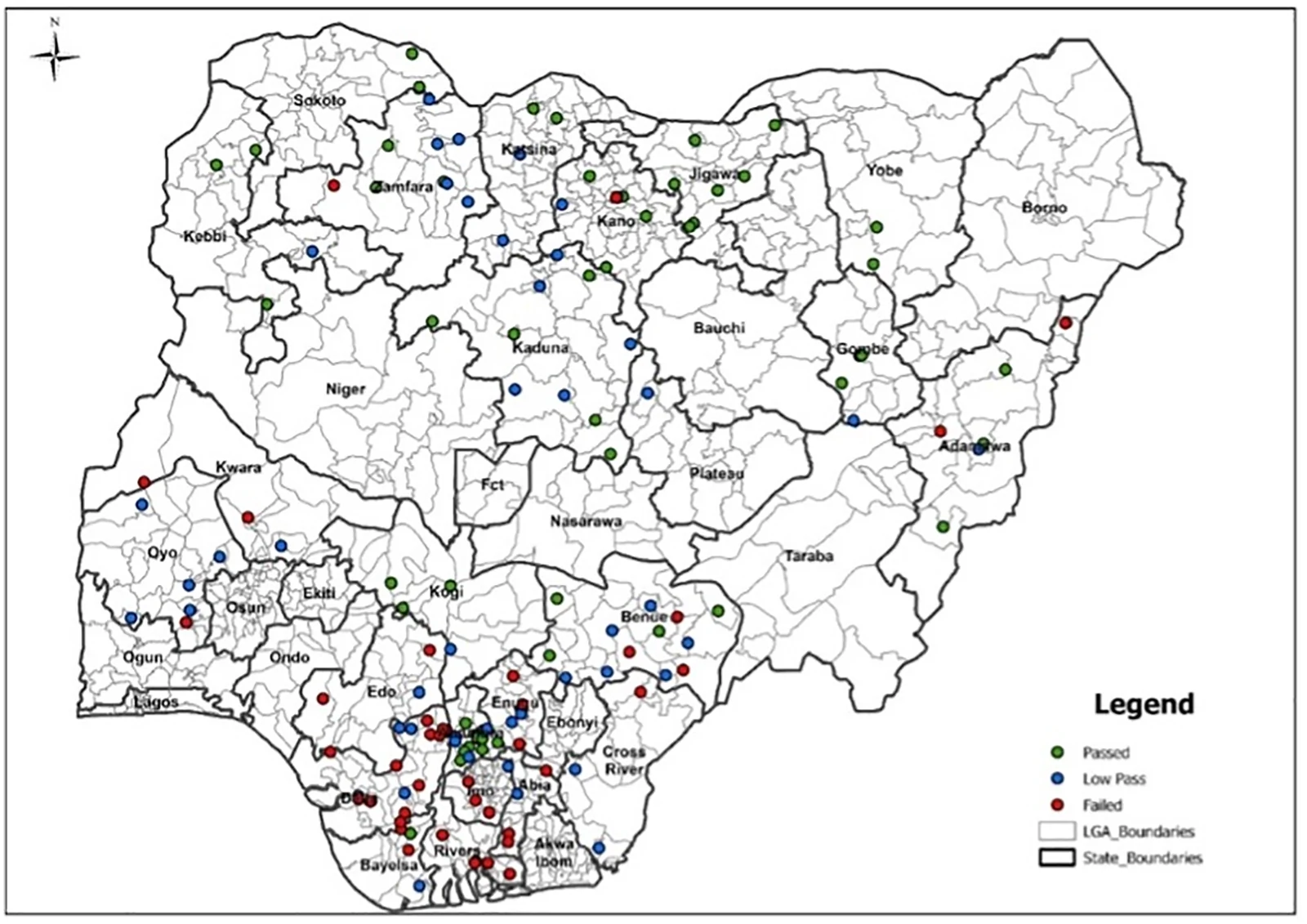
Map of Nigeria showing the distribution of health facilities assessed for readiness for providing surgical care for hydrocoele patients. This map was created using primary data collected by the authors (S2 Data) and developed in ArcGIS v10.8 software. Shapefiles for Nigeria, including boundary polygons across all administrative levels, were sourced from publicly accessible databases available at Humanitarian Data Exchange, and are licensed under CC BY 4.0 (https://data.humdata.org/dataset/cod-ab-nga). No changes were made to the original shapefiles. The authors grant permission for the reuse of this map without restriction, provided appropriate credit is given to the source.

**Fig 8:**
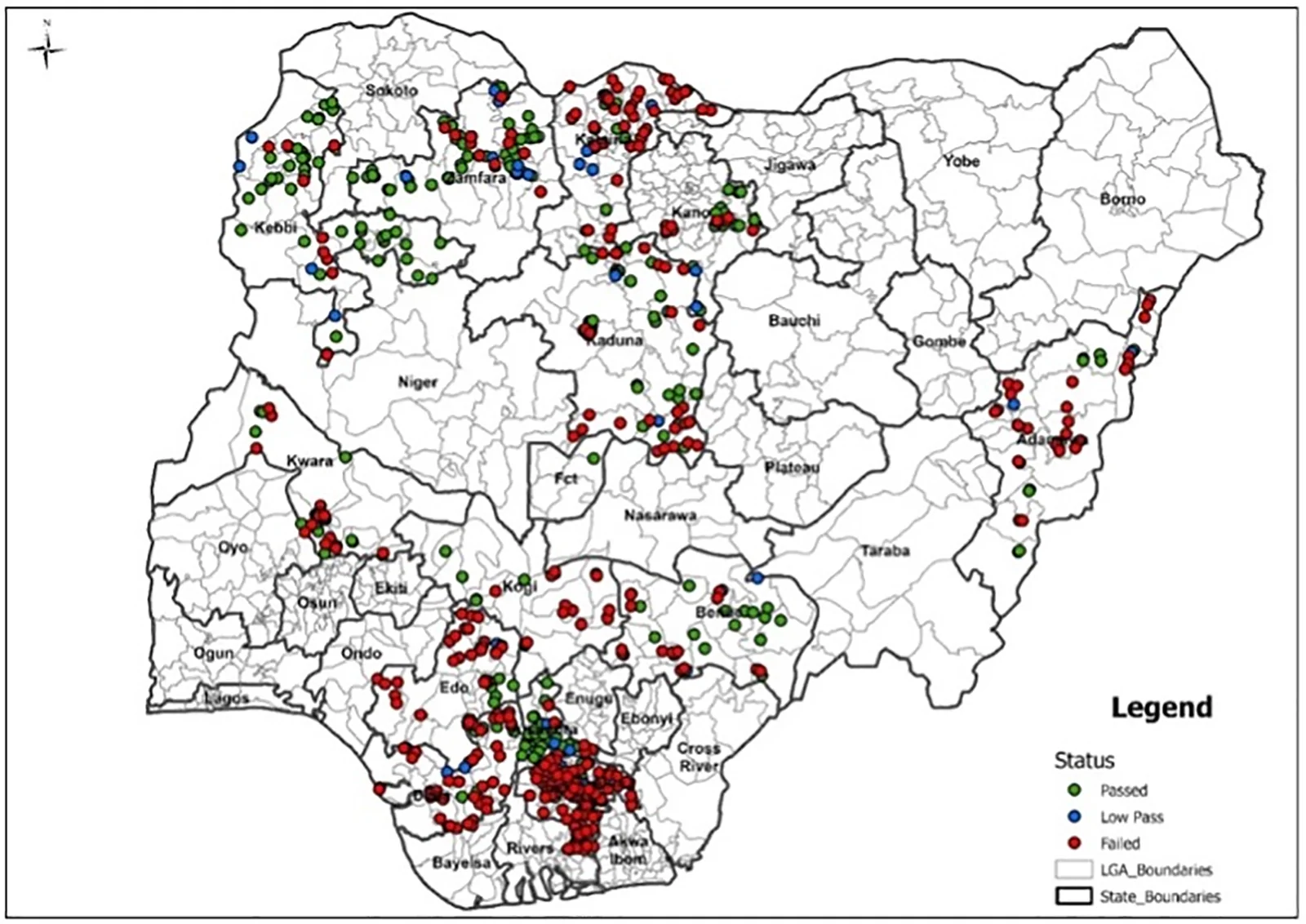
Map of Nigeria showing the distribution of health facilities assessed for readiness for providing care for lymphoedema patients. This map was created using primary data collected by the authors (S2 Data) and developed in ArcGIS v10.8 software. Shapefiles for Nigeria, including boundary polygons across all administrative levels, were sourced from publicly accessible databases available at Humanitarian Data Exchange, and are licensed under CC BY 4.0 (https://data.humdata.org/dataset/cod-ab-nga). No changes were made to the original shapefiles. The authors grant permission for the reuse of this map without restriction, provided appropriate credit is given to the source.

### Capacity building for MMDP care

The NLFEP conducted training at different levels to empower the health workforce with the skill set needed to provide quality care and management to all identified cases that came into the health system. A WHO team of trainers and consultants conducted training-of-trainers sessions using the Filariacele Anatomical Surgical Task Trainer (FASST) module, supplemented by demonstrations on mannequins and live surgical procedures [31]. Thereafter, a structured cascading plan was rolled out in clusters and selected surgeons from all six geopolitical zones were trained on the FASST procedure to improve surgical outcomes. Additionally, training of trainers for lymphoedema management was also done to ensure harmonization in the provision of care by health workers from all the primary care centers.

### Tracking cases for morbidity management and disability prevention services

Tracking of cases is useful for estimating the burden of morbidity, guiding surgical planning, monitoring access to care, and documenting progress toward MMDP targets. The hydrocele tracker, a standardized programmatic data collection and reporting tool, is used by the programme to systematically record, monitor, and report the identification, referral, surgical management, and follow-up of hydrocele cases [32]. The MMDP arm of the NLFEP leveraged MAM campaigns in endemic LGAs to facilitate the identification of suspected morbidity cases, specifically hydrocele and lymphoedema. CDDs in collaboration with frontline health workers, compiled waiting lists of suspected cases during MAM activities. These community-level data served as the foundation for morbidity case estimation, enabling the program to assess the disease burden across implementation areas. Training of trainers on the hydrocele tracker and cascading of the training equipped programme staff with the requisite skills to track patients across the continuum of pre-operative, intra-operative, and post-surgical care.

Table 6 presents the number of lymphoedema, and hydrocele cases reported and managed across accredited health facilities in Nigeria between 2021 and 2025, disaggregated by geopolitical zones.

**Table 6:** Number of hydrocoele and lymphoedema cases reported and managed across accredited health facilities between 2021 and 2025.

	Zones						
	North-central	North-east	North-west	South-east	South-south	South-west	
	N (%)	N (%)	N (%)	N (%)	N (%)	N (%)	Total (%)
**Indicators**							
No. of States in zone	7	6	7	5	6	6	37
No. of States reporting cases (%)	7 (100)	6 (100)	7 (100)	5 (100)	6 (100)	6 (100)	37 (100)
No. of LGAs in zone	121	112	186	95	123	137	774
No. of LGAs reporting cases (%)	114 (94.3)	65 (58.1)	126 (67.8)	74 (77.9)	80 (65.1)	110 (80.3)	569 (73.6)
**Lymphoedema**							
No. of LGAs reported (%)	104 (86.0)	65 (58.1)	78 (42.0)	55 (57.9)	52 (42.3)	96 (70.1)	450 (58.2)
No. of cases reported	2490	2135	1383	712	367	737	7824
No. of cases managed (%)	2047 (82.3)	2004 (93.9)	1002 (72.5)	127 (17.9)	56 (15.3)	592 (80.4)	5828 (74.5)
**Hydrocele**							
No. of LGAs reported (%)	98 (81.0)	76 (67.9)	110 (59.2)	73 (76.9)	70 (57.0)	76 (55.5)	503 (65.0)
No. of cases reported	2523	7159	7610	1221	2875	807	22195
No. of cases managed (%)	1603 (63.6)	6957 (97.2)	5705 (75.0)	322 (26.4)	1261 (43.9)	202 (25.1)	16050 (72.4)

N or No.: Number of cases; LGAs: Local Government Areas.

The proportion LGAs reporting cases varied across zones, with the North-central zone reporting from 114 of 121 LGAs (94.3%), the North-east from 65 of 112 LGAs (58.1%), the North-west from 126 of 186 LGAs (67.8%), the South-east from 74 of 95 LGAs (77.9%), the South-south from 80 of 123 LGAs (65.1%), and the South-west from 110 of 137 LGAs (80.3%).

For hydrocele, a total of 503 LGAs reported cases, totaling 22,195 cases, with 72.4% of these cases being managed across the country (Table 6). By zones, the Northeast and North-west reported the highest number of cases, with 7,159 and 7,610 cases respectively, and 97.2% and 75% of these being managed. In contrast, the South-east and South-west zones reported fewer cases, 1,221 and 807 respectively, with lower proportions managed (26.4% and 25.1%). The North-central zone reported 2,523 hydrocele cases, of which 1,603 (63.6%) were managed, while the South-south reported 2,875 cases with 1,261 (43.9%) managed (Table 5).

For lymphoedema, 450 LGAs across the country reported cases, accounting for 7,824 cases, of which 5,828 (74.5%) were actively managed. The North-central zone had the highest number of LGAs reporting lymphoedema cases (104, 86%) with 2,490 cases reported and 2,047 (82.3%) managed. The North-east had 65 LGAs reporting 2,135 cases, of which 2,004 (93.9%) were managed, while the North-west reported 78 LGAs with 1,383 cases, 1,002 (72.5%) of which were managed. The South-east and South-south zones had relatively lower numbers of lymphoedema cases, with 712 and 367 cases reported respectively, and only 17.9% and 15.3% managed. The South-west reported 737 cases from 96 LGAs, with 592 (80.4%) being managed (Table 6). Overall, the reporting trend for both hydrocele and lymphoedema was low at inception of the program, however sustained stakeholder’s engagement and collaboration led to increased reporting in the subsequent years. The morbidity estimates generated from this process were instrumental in informing strategic planning, resource allocation, and prioritization of MMDP interventions at the state level (Fig 9).

**Fig 9:**
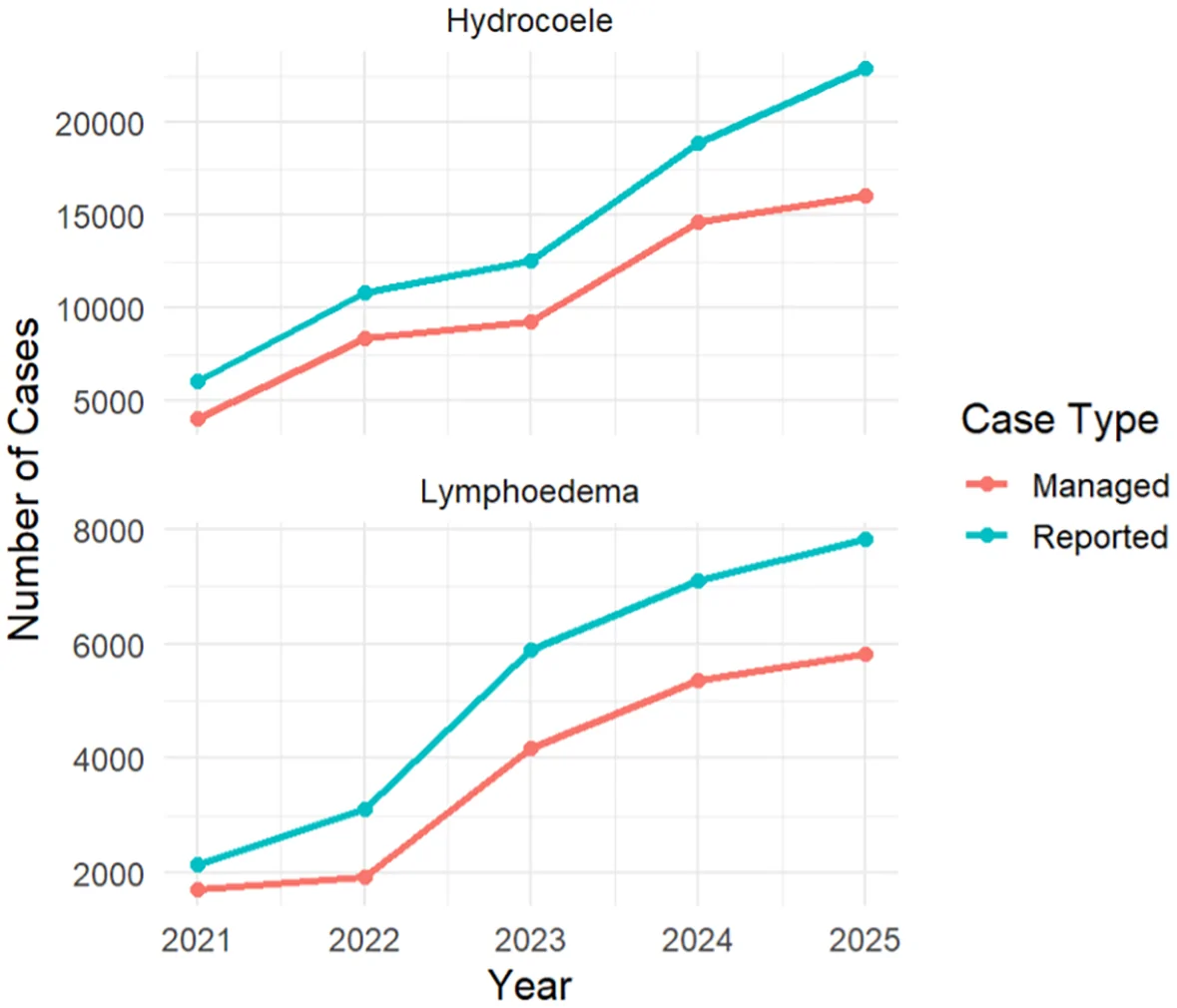
Line graph showing the trend of cases reported and managed for lymphoedema and hydrocoele in Nigeria between 2021 and 2025.

### Post elimination surveillance strategies

Following WHO guidelines [11], continued PTS remains important until all IUs in Nigeria achieve interruption of transmission. The NLFEP has recommended continued surveillance in Nasarawa and Plateau States, especially in hard-to-reach areas and endemic border LGAs, where the risk of re-introduction may be higher. However, PTS activities have largely been limited to operational research studies involving both children and adults in selected LGAs, with broader implementation constrained by financial limitations [33].

### Integrated vector control initiatives

Vector control interventions, such as the distribution of ITNs, are recommended for LF control but are not currently implemented as part of NFLEP activities. However, ITN distribution is routinely conducted through the national malaria elimination programme [19,26]. As of 2024, 59.2% of households owned at least one ITN, with an average of 1.3 ITNs per household. However, only 31.5% of households had adequate ITN access, defined as at least one ITN for every two household members. Nationally, 42.5% of children under five years and 45.5% of pregnant women reported sleeping under an ITN the night preceding the survey (Table 7).

**Table 7:** Ownership and utilization rate of treated bed-nets across the country as of 2024.

Zone	State	% of HH with at least one ITNs	Average number of ITNs per HH	% of HH with at least one ITN for every two persons	% of children under age 5 who slept under an ITNs	% of pregnant women who slept under an ITNs
**South-east**	Abia	25.4	0.4	13.3	20.0	1.1
	Anambra	56.8	1.3	35.2	37.0	38.3
	Ebonyi	74.4	1.7	32.9	31.6	36.5
	Enugu	17.0	0.2	6.5	13.3	7.1
	Imo	32.8	0.6	17.5	11.3	8.2
	Total	42.6	0.9	22.0	24.2	23.4
**South-west**	Ekiti	25.4	0.4	14.8	16.1	19.3
	Lagos	31.9	0.5	18.1	19.2	18.8
	Ogun	33.7	0.5	18.3	10.7	1.9
	Ondo	58.4	1.1	41.8	31.5	40.1
	Osun	59.0	0.9	37.4	39.8	25.1
	Oyo	39.1	0.6	19.4	27.5	15.5
	Total	40.1	0.6	23.6	22.6	20.1
**South-south**	Akwa Ibom	70.9	1.9	59.9	25.4	29.2
	Bayelsa	38.9	0.6	15.0	34.6	20.6
	Cross River	80.2	1.8	64.6	51.4	30.4
	Delta	58.0	1.3	40.5	39.5	31.0
	Edo	21.0	0.4	9.0	15.4	11.2
	Rivers	29.1	0.5	14.2	23.6	18.1
	Total	48.6	1.1	33.6	30.6	23.6
**North-east**	Adamawa	78.6	2.0	48.9	47.9	42.6
	Bauchi	76.4	2.1	44.8	50.5	53.5
	Borno	57.3	0.9	20.6	39.6	36.1
	Gombe	79.2	1.9	33.7	52.2	59.1
	Taraba	70.9	1.7	40.6	42.6	56.2
	Yobe	73.4	1.9	34.1	62.0	54.5
	Total	71.9	1.7	37.4	49.3	49.8
**North-west**	Jigawa	86.7	2.2	38.9	55.2	73.7
	Kaduna	72.0	1.6	33.0	43.7	50.9
	Kano	87.1	2.6	46.5	67.5	82.4
	Katsina	75.8	1.7	28.3	45.7	51.6
	Kebbi	81.5	1.8	30.9	52.6	60.1
	Sokoto	78.5	1.9	32.3	19.3	18.8
	Zamfara	89.7	2.2	40.3	64.8	74.6
	Total	81.1	2.0	36.7	52.2	59.6
**North-central**	Benue	82.3	2.0	60.2	58.5	58.7
	FCT, Abuja	40.3	0.6	19.4	36.6	27.9
	Kogi	38.8	0.7	20.1	26.8	30.1
	Kwara	36.9	0.6	16.4	12.5	16.7
	Nasarawa	47.0	0.8	20.4	37.4	24.9
	Niger	72.0	1.8	42.8	48.4	55.1
	Plateau	37.5	0.8	19.9	22.8	25.2
	Total	54.2	1.2	31.2	37.6	38.5
**Total**		59.2	1.3	31.5	42.5	45.5

Table was extracted from the Nigeria DHS 2023–24 report [19] and based on children and pregnant women who stayed in the household the night before the interview; LGAs: Local Government Areas; HH: Household; ITNs: Insecticide-treated net (ITN) also known as long-lasting insecticidal net (LLIN).

Marked geographical disparities were observed across the zones. The North-west recorded the highest household ownership of at least one ITN (81.1%) and the highest average number of ITNs per household (2.0). Utilization patterns generally mirrored ownership levels, with the North-west reporting the highest utilization among pregnant women (59.6%) and among the highest utilization rates among children under five years (52.2%). In contrast, the South-west recorded the lowest utilization rates among both children under five (22.6%) and pregnant women (20.1%) (Table 7).

At the state level, the highest household ownership of at least one ITN was observed in Zamfara (89.7%), Kano (87.1%), Jigawa (86.7%), Benue (82.3%), Kebbi (81.5%), and Cross River (80.2%). The highest utilization among children under five was recorded in Kano (67.5%), Zamfara (64.8%), and Yobe (62.0%). Plateau and Nasarawa States reported relatively low ownership levels (37.5% and 47.0%, respectively), with utilization rates ranging from 22.8% to 37.4% among children under five and from 24.9% to 25.2% among pregnant women (Table 7). To support continued promotion of long-lasting insecticidal nets (LLINs), the Carter Center has assisted in the distribution of 79,647 LLINs since 2017, specifically targeting cross-border communities in Plateau and Nasarawa States [33].

## Discussion

The NLFEP has achieved substantial progress in reducing the burden of LF and advancing toward the 2030 global elimination target [11]. Prior to this study, elimination had only been documented in Plateau and Nasarawa States [33], while significant progress had been reported in the Federal Capital Territory [34]. This study therefore provides a more comprehensive assessment of Nigeria’s LF elimination programme since its inception in 2000. Our findings show that LF mapping has been completed nationwide between 2000 and 2011, and all 583 IUs had initiated MAM, with more than 90% of them completing the minimum effective MAM rounds required for impact assessment. By 2025, approximately 60% of IUs had stopped MAM across 30 of Nigeria’s 37 states. Furthermore, a growing number of IUs had progressed along the elimination pathway, with approximately 7% passing TAS-2, and about 5% passing TAS-3. Based on observed programme trends and current implementation schedules, we estimate that, if current progress is maintained, MAM could be stopped in 497 (85.2%) of endemic IUs by 2030, with 309 IUs potentially achieving elimination. These estimates are scenario-based programme projections rather than predictions and should be interpreted cautiously, as future progress may be affected by changes in transmission dynamics, programme implementation, and other operational factors.

In parallel with efforts to interrupt transmission, the NLFEP has strengthened health systems for MMDP through improvements in health-facility readiness, capacity building of healthcare workers, and integration of MMDP activities into routine immunization and polio services, seasonal malaria chemoprevention campaigns, MAM campaigns, and disease surveillance and notification systems. For instance, trachoma trichiasis case-finding activities were also leveraged to identify individuals with LF-related morbidity [35]. This integrated approach expanded opportunities for case detection, optimized the use of existing health-system resources, minimized duplication of efforts, and improved service delivery efficiency. Beyond strengthening service availability, emerging evidence indicates that these interventions have generated substantial benefits for affected individuals, with improvements in mobility, reduced pain, enhanced psychological well-being, increased social participation, and greater economic productivity [36,37].

Despite the considerable progress recorded in both transmission interruption and morbidity management, important operational and programmatic challenges remain that could impede attainment of the 2030 elimination targets. Foremost among these is insecurity associated with terrorism, banditry, and communal clashes, which continues to restrict access to some communities and remains a major obstacle to the implementation of MAM, community sensitization activities, and epidemiological assessments [38–41]. The consequences of these disruptions are particularly evident in northern Nigeria, especially in Borno State, where prolonged insecurity associated with terrorism and banditry delayed the initiation of MAM until 2019 and resulted in most endemic LGAs receiving only 1–4 effective treatment rounds [32]. Similar disruptions arising from communal conflicts, insurgency, kidnappings, and other security threats have been reported in several endemic areas across the country. To manage these challenges, the NLFEP has adopted context-specific delivery approaches including leveraging local intelligence to assess security risks, implementing proxy training models for CDDs, conducting “hit-and-run” treatment campaigns under security escort, and providing services through safer neighboring communities accessible to affected populations [42]. While these adaptations have enabled continued programme activities in some settings, its implementation across Nigeria remains largely ad-hoc, with varying levels of risk tolerance and operational flexibility among donors and implementing partners. Consequently, there is a need for a nationally coordinated framework that strengthens collaboration among NTD stakeholders while guiding security risk assessment and adaptive implementation approaches to ensure the safe and effective delivery of NTD interventions in insecurity-affected settings.

Furthermore, funding constraints and limited operational resources continue to impede progress toward LF elimination by delaying medicine distribution, coverage evaluation surveys, and epidemiological assessments [41–43]. These constraints were more pronounced in southern states and have the potential to undermine the quality and effectiveness of MAM implementation. Delays in conducting coverage evaluation surveys are particularly problematic because these assessments are critical for identifying systematically missed populations and addressing treatment non-compliance and operational bottlenecks that limit programme reach [41–43]. Without timely assessments, opportunities to refine implementation strategies and improve treatment coverage may be lost [44,45]. In addition, delays in epidemiological assessments can postpone critical programmatic decisions regarding areas eligible to stop MAM or progress along the elimination pathway. Such delays are not only operationally inefficient but may also result in the continued implementation of MAM in areas where transmission has already been interrupted but cannot be formally verified due to insufficient resources for assessment [33]. Sustained domestic financing, continued partner engagement, and strategic resource mobilization will therefore be essential moving forward.

One potential strategy for sustaining programme implementation in the face of limited resources is the mainstreaming of MAM and other NTD interventions within broader Universal Health Coverage (UHC) and primary healthcare frameworks [46]. There is growing recognition that integrating NTD services into routine health systems can strengthen domestic resource mobilization, reduce reliance on external funding, and enhance the long-term sustainability of programme operations [47,48]. In parallel, collaborative mechanisms among implementing partners—including joint planning, resource sharing, and coordinated allocation of available funding—have emerged as important approaches for mitigating resource constraints and maintaining programme continuity. Such collaborative approaches have been piloted in several Nigerian states for diseases including onchocerciasis, schistosomiasis, and soil-transmitted helminthiasis [48], as well as for broader public health priorities such as maternal and child health [47]. These partnerships have facilitated the timely implementation of coverage surveys and operational assessments [26,44,49,50], improved identification of missed populations, enabled refinement of delivery strategies [44,45], strengthened community engagement, and enhanced treatment compliance. Expanding these collaborative and integrated approaches will be critical for addressing operational gaps, improving programme efficiency, and accelerating progress toward the 2030 LF elimination targets.

Beyond the challenges, the WHO recommends the use of complementary vector-control interventions as part of integrated approaches to accelerate and sustain the elimination of lymphatic filariasis in endemic settings [11]. Although vector-control activities are not currently a core component of NLFEP implementation [51], there is growing evidence that ITNs can contribute to reducing LF transmission, particularly in areas where *Anopheles* mosquitoes serve as the principal vectors [52,53]. Findings from the 2025 NDHS report indicate that ITN ownership, access, and utilization remain suboptimal in many endemic areas of Nigeria [19]. Strengthening access to and utilization of vector-control interventions may therefore provide additional benefits for both LF and malaria control programmes. As Nigeria moves closer to achieving LF elimination targets, greater consideration should be given to integrating complementary vector-management strategies within broader elimination and post-elimination frameworks to help sustain gains and reduce the risk of recrudescence.

Finally, as an increasing number of IUs progress toward TAS-3 and elimination as a public health problem, it is imperative that standardized post-validation surveillance systems are established within the NLFEP [33]. A clearly defined surveillance strategy should include targeted investigations of antigen-positive individuals and clusters, continued monitoring through sentinel and spot-check sites, integration of LF testing into large-scale population-based surveys, and the use of xenomonitoring where feasible [33,54]. Attention should be given to border communities, migrant and mobile populations, and other settings at increased risk of reintroduction or resurgence [38–40,55]. Strengthening post-elimination surveillance will be essential to safeguarding elimination gains and ensuring the long-term interruption of transmission.

### Study limitations

This study has several limitations. First, the analysis relied primarily on routinely collected programme data spanning more than two decades. Consequently, data completeness, reporting practices, and data-management systems may have varied across states and over time, and some programme records may have been incomplete or unavailable. Second, LF diagnostic tools and survey protocols evolved during the study period in response to changes in WHO recommendations, which may limit the direct comparability of some indicators over time. Third, programme activities were implemented at the state level with support from different implementing and development partners. Variations in the availability of resources, partner support, and implementation capacity may therefore have contributed to differences in programme performance across states. Fourth, epidemiological assessments were not conducted simultaneously across all endemic IUs. Consequently, observed differences in programme status across states may partly reflect variations in the timing and duration of programme implementation and assessment. Finally, the projections to 2030 are based on observed programme trends and current implementation schedules and should not be interpreted as definitive predictions. Future progress may be influenced by transmission dynamics, funding availability, insecurity, programme implementation capacity, and other operational factors.

## Conclusion

Nigeria has made substantial progress toward the elimination of lymphatic filariasis through the implementation of MAM and MMDP strategies. Based on observed programme trends and current implementation schedules, and assuming that current progress is maintained, MAM could be stopped in approximately 85% of endemic IUs by 2030, while approximately 53% of IUs could achieve elimination. However, interventions need to be intensified in IUs lagging in MAM implementation and epidemiological assessments. Sustaining progress will require continued political commitment, increased domestic ownership, strategic partnerships, and targeted investments to support timely assessments, adaptive programme implementation, and robust surveillance in areas where transmission has been interrupted. Addressing these challenges while consolidating recent gains will be critical to achieving, validating, and sustaining the elimination of lymphatic filariasis as a public health problem in Nigeria.

## Supporting information

S1 Data Dataset. (CSV)

S2 Data Dataset 2. (XLSX)
