# Supplementary material for: Two decades of lymphatic filariasis elimination efforts in Nigeria: History, progress, challenges, and prospects for achieving the 2030 targets
Source: PLoS Negl Trop Dis. 2026 Nov 10;20(11):e0014860. doi: 10.1371/journal.pntd.0014860 (PMC13766621; doi:10.1371/journal.pntd.0014860)
Supplement: Supplementary file 1 [file pntd.0014860.s005.pdf]

PLOS Neglected Tropical Diseases

Two Decades of Lymphatic Filariasis Elimination Efforts in Nigeria: History, Progress, Challenges, and Prospects for Achieving the 2030 Targets

--Manuscript Draft--

**Manuscript Number:**	PNTD-D-26-00304R1
**Full Title:**	Two Decades of Lymphatic Filariasis Elimination Efforts in Nigeria: History, Progress, Challenges, and Prospects for Achieving the 2030 Targets
**Short Title:**	Two Decades of Lymphatic Filariasis Elimination Efforts in Nigeria
**Article Type:**	Research Article
**Keywords:**	Lymphatic filariasis elimination; Mass drug administration (MAM); Transmission assessment surveys (TAS); Nigeria; Neglected tropical diseases (NTDs)
**Corresponding Author:**	Hammed Oladeji Mogaji, Ph.D<br>Marian University<br>Indianapolis, UNITED STATES OF AMERICA
**Corresponding Author Secondary Information:**	
**Corresponding Author's Institution:**	Marian University
**Corresponding Author's Secondary Institution:**	
**First Author:**	Oladele B. Akogun
**First Author Secondary Information:**	
**Order of Authors:**	Oladele B. Akogun
	Hammed O Mogaji, Ph.D
	Babatunde Adewale
	Dung D. Pam
	Joseph S. Terhemen
	Hanafi Oladele
	Fatai Oyediran
	Chinwe Okoye
	Emeka Uzoma
	Olumide Falodun
	Ununumah Egbelu
	Kehinde G. Busari
	Sunday Isiyaku
	Joy Shuaibu
	Anita Gwom
	FRANCISCA OLAMIJU
	Emmanuel Miri
	Adamu Sallau
	Samuel Omoi
	Innocent Emereuwa
	Rita Oguntoyinbo

Powered by Editorial Manager® and ProduXion Manager® from Aries Systems Corporation

	Mohammed Aliu
	Esther Ekong
	Adamani William
	Ijeoma Achu
	Osagie Omorogie
	Rinpan Ishaya
	Cephas Ityonzughul
**Order of Authors Secondary Information:**	
**Abstract:**	Introduction<br>Lymphatic filariasis (LF) is one of the priority neglected tropical diseases targeted for elimination by 2030 in 58 endemic countries, including Nigeria, through a dual strategy of interrupting transmission and providing morbidity management and disability prevention (MMDP) services. Only two African countries (Togo and Malawi) have eliminated the disease. The extent to which other African countries have progressed towards elimination is uncertain. It will be helpful to understand the progress endemic countries are making towards elimination. Here, a comprehensive review of the history, progress and prospects for achieving 2030 elimination targets in Nigeria, the most endemic country for LF in Africa was provided. This review will be useful in evaluating the extent of investment that is required before elimination can be achieved in Nigeria. Such information will be helpful for assessing other countries with similar epidemiological factors<br><br>Methods<br>Programmatic data was extracted from the National Lymphatic Filariasis Elimination Programme (NLFEP) on endemicity, treatment history, mass administration of medicines (MAM) coverage, epidemiological assessments, and morbidity management across 774 Local Government Areas (LGAs) in Nigeria. Status of endemic LGAs were identified, and those that had completed a minimum of five effective MAM rounds were assessed on their progress of elimination using the recommended Transmission Assessment Survey (TAS) and Morbidity Management and Disability Prevention (MMDP) guidelines. The number of LGAs expected to achieve LF elimination by 2030 was forecasted. All statistical analyses and visualizations were descriptive and performed in R software (v4.5.2).<br>Findings<br>Nigeria completed LF mapping in all 774 LGAs between 2000 and 2011, revealing 583 endemic LGAs. All the endemic LGAs received 3 to 15 rounds of MAM between 2000 and 2015, and 529 (90.7%) completed the required five effective MAM rounds. As of 2024, 445 (76.3%) passed EMS, 346 (59.3%) passed TAS-1 and stopped MAM, while 40 (6.9%) passed TAS-2 and 30 (5.1%) passed TAS-3 (achieve elimination). By 2030, we project that MAM would have stopped in 497 EUs i.e., 85.2% of all endemic IUs, and 309 (62.2%) of those would have passed TAS-3 (achieve elimination). However, under a conservative assumption that 3-5% of planned surveys can fail, these figures decline to 454 (77.9%) EUs stopping MAM and 284 achieving elimination. For MMDP, Lymphoedema was reported in 450 LGAs, totaling 7,824 cases, with 5,828 (74.5%) actively managed. Hydrocele was reported across 503 LGAs, with 22,195 documented cases, 72.4% of which received management.<br>Conclusion<br>Nigeria has made substantial progress toward the elimination of lymphatic filariasis. The overall trajectory suggests that Nigeria remains on course to stop MAM and achieve elimination in 85% and 53% of all endemic IUs, respectively
**Suggested Reviewers:**	
**Opposed Reviewers:**	
**Additional Information:**	
**Question**	**Response**
**Government Employee**	No - No authors are employees of the U.S. government.

Are you or any of the contributing authors an employee of the United States government?<br><br>Manuscripts authored by one or more US Government employees are not copyrighted, but are licensed under a CC0 Public Domain Dedication, which allows unlimited distribution and reuse of the article for any lawful purpose. This is a legal requirement for US Government employees.<br><br>This will be typeset if the manuscript is accepted for publication.	
**Financial Disclosure**<br><br>Enter a financial disclosure statement that describes the sources of funding for the work included in this submission. Review the submission guidelines for detailed requirements. View published research articles from *PLOS Neglected Tropical Diseases* for specific examples.<br><br>This statement is required for submission and **will appear in the published article** if the submission is accepted. Please make sure it is accurate.<br><br>**Funded studies**<br>Enter a statement with the following details:<br>• Initials of the authors who received each award<br>• Grant numbers awarded to each author<br>• The full name of each funder<br>• URL of each funder website<br>• Did the sponsors or funders play any role in the study design, data collection and analysis, decision to publish, or preparation of the manuscript?<br><br>Did you receive funding for this work?	The author(s) received no specific funding for this work.
**Competing Interests**	The authors have declared that no competing interests exist

On behalf of all authors, disclose any competing interests that could be perceived to bias this work.<br><br>This statement will be typeset if the manuscript is accepted for publication.<br><br>Review the instructions link below and PLOS NTDs' competing interests policy to determine what information must be disclosed at submission.	
**Data Availability**<br><br>Before publication, Authors are required to make fully available and without restriction all data underlying their findings. Sharing the data underlying your findings increases the visibility, credibility, and impact of your work, while supporting transparency and future scientific discovery. Please see our PLOS Data Policy page for detailed information on this policy.<br><br>A **Data Availability Statement**, detailing where the data can be accessed, is required at first submission. Insert your Data Availability Statement in the box below.<br><br>Please see the data reporting section of our submission guidelines for instructions on what you need to include in your Data Availability Statement. Examples of acceptable Data Availability Statements are included here for reference:<br>1. The minimal data set [and accompanying code, where generated] is available at [repository name] via [DOI hyperlink]<br>2. No data was generated by this study. The following existing data sources were used: [dataset name] from [dataset location] available via [web address or DOI hyperlink].<br>3. For Study Protocols: No datasets were	The datasets analyzed during this study are also provided as supplementary materials accompanying this article.

generated or analysed during the current study. All relevant data from this study will be made available upon study completion.<br><br>*PLOS allows rare exemptions to address legal and ethical concerns. If you have legal or ethical restrictions, please use the box below to detail these in full sentences for the Journal team to consider.*<br><br>This statement will be typeset if the manuscript is accepted for publication.	

Dr. Hammed O. Mogaji
Corresponding Author
Public Health Program, Department of Applied and Behavioral Sciences
Marian University, Indianapolis, USA
Email: mogajihammed@gmail.com

Submission of Manuscript for Publication Consideration in PLoS Neglected Tropical Diseases.

Dear Editor,

We are pleased to submit our manuscript entitled **"Two Decades of Lymphatic Filariasis Elimination Efforts in Nigeria: History, Progress, Challenges, and Prospects for Achieving the 2030 Targets"** for consideration for publication in your journal.

Lymphatic filariasis (LF) remains one of the most important neglected tropical diseases targeted for global elimination by 2030. Nigeria carries the largest LF burden in Africa, and progress within the country is therefore critical to achieving regional and global elimination goals. Despite over two decades of implementation, a comprehensive synthesis of Nigeria's programmatic experience, progress, and remaining challenges has been lacking. This manuscript addresses this gap by providing a detailed national-level analysis of LF elimination efforts spanning 24 years (2000–2024), drawing on programmatic data from the National Lymphatic Filariasis Elimination Programme (NFLEP).

Using nationally representative implementation and evaluation data across all 774 Local Government Areas, we provide the evolution of mapping, mass administration of medicines, epidemiological assessments, transmission assessment surveys, and morbidity management and disability prevention services. This manuscript further provides projections toward the 2030 elimination targets under both optimistic and conservative scenarios, offering critical insights into operational bottlenecks, funding gaps, and contextual challenges such as insecurity and partner transitions. Importantly, the findings highlight both the substantial gains achieved and the strategic investments required to sustain progress and prevent programmatic reversal.

The manuscript is timely and highly relevant to global health practitioners, policymakers, and implementation partners working in neglected tropical diseases and elimination programs. Nigeria's experience provides transferable lessons for other endemic countries facing similar epidemiological, operational, and financing constraints. By documenting large-scale implementation experience, this work contributes to the growing literature on program sustainability, adaptive implementation, and health system coordination in disease elimination settings.

This manuscript represents original work that has not been published elsewhere and is not under consideration by another journal. All authors have reviewed and approved the manuscript and agree with its submission. The authors declare no conflicts of interest. Ethical and programmatic approvals were obtained through appropriate national program structures where required.

Given the journal's strong focus on infectious diseases, implementation science, and global health impact, we believe this manuscript will be of considerable interest to your readership.

Thank you for your time and consideration. We look forward to your response.

Sincerely,

On behalf of all co-authors

Click here to access/download;Manuscript;Manuscript_clean.docx

Two Decades of Lymphatic Filariasis Elimination Efforts in Nigeria: History, Progress, Challenges, and Prospects for Achieving the 2030 Targets

Oladele B. Akogun[1†], Hammed O. Mogaji[2†*], Babatunde Adewale[3], Dung D. Pam[4], Joseph S. Terhemen[5], Hanafi Oladele[5], Fatai Oyediran[6], Chinwe Okoye[6], Emeka Uzoma[6], Olumide Falodun[6], Ununumah Egbelu[6], Kehinde G. Busari[6], Sunday Isiyaku[7], Joy Shuaibu[5], Anita Gwom[5], Francisca Olamiju[8], Emmanuel Miri[9], Adamu Sallau[8], Samuel Omoi[10], Innocent Emereuwa[11], Rita Oguntoyinbo[12], Mohammed Aliu[13], Esther Ekong[13], Adamani William[10], Ijeoma Achu[8], Osagie Omorogie[12], Ishaya Rinpan[11], Cephas ItyonzughuI[9]

[1]The Health Programme Common Heritage Foundation Abuja, Abuja, Nigeria ; [2]Public Health Program, Department of Applied and Behavioral Sciences, College of Arts and Sciences, Marian University, Indianapolis, Indiana, United States of America; [3]Public Health and Epidemiology Department, Nigerian Institute of Medical Research Yaba, Lagos, Nigeria; [4]Department of Zoology, University of Jos, Nigeria; [5]Sightsavers, Abuja, Nigeria; [6]Neglected Tropical Diseases Programme, Federal Ministry of Health and Social Welfare, Abuja, Nigeria; [7]Reaching the Last Mile for Onchocerciasis and Lymphatic Filariasis in Africa; [8]Mission To Save The Helpless, Ikeja, Lagos State, Nigeria; [9]The Carter Center , Jos, Nigeria; [10]Christoffel Blinden Mission International, Abuja, Nigeria; [11]Health and Development Support Programme, Abuja, Nigeria; [12]Amen Health Care and Empowerment Foundation, Abuja, Nigeria; [13]Helen Keller International, Abuja, Nigeria

*****Corresponding author: mogajihammed@gmail.com

[†]These authors contributed equally to this work and share first authorship.

Abstract

Introduction

Lymphatic filariasis (LF) is one of the priority neglected tropical diseases targeted for elimination by 2030 in 58 endemic countries, including Nigeria, through a dual strategy of interrupting transmission and providing morbidity management and disability prevention (MMDP) services. Only two African countries (Togo and Malawi) have eliminated the disease. The extent to which other African countries have progressed towards elimination is uncertain. It will be helpful to understand the progress endemic countries are making towards elimination. Here, a comprehensive review of the history, progress and prospects for achieving 2030 elimination targets in Nigeria, the most endemic country for LF in Africa was provided. This review will be useful in evaluating the extent of investment that is required before elimination can be achieved in Nigeria. Such information will be helpful for assessing other countries with similar epidemiological factors

Methods

Programmatic data was extracted from the National Lymphatic Filariasis Elimination Programme (NLFEP) on endemicity, treatment history, mass administration of medicines (MAM) coverage, epidemiological assessments, and morbidity management across 774 Local Government Areas (LGAs) in Nigeria. Status of endemic LGAs were identified, and those that had completed a minimum of five effective MAM rounds were assessed on their progress of elimination using the recommended Transmission Assessment Survey (TAS) and Morbidity Management and Disability Prevention (MMDP) guidelines. The number of LGAs expected to achieve LF elimination by 2030

was forecasted. All statistical analyses and visualizations were descriptive and performed in R software (v4.5.2).

Findings

Nigeria completed LF mapping in all 774 LGAs between 2000 and 2011, revealing 583 endemic LGAs. All the endemic LGAs received 3 to 15 rounds of MAM between 2000 and 2015, and 529 (90.7%) completed the required five effective MAM rounds. As of 2024, 445 (76.3%) passed EMS, 346 (59.3%) passed TAS-1 and stopped MAM, while 40 (6.9%) passed TAS-2 and 30 (5.1%) passed TAS-3 (achieve elimination). By 2030, we project that MAM would have stopped in 497 EUs i.e., 85.2% of all endemic IUs, and 309 (62.2%) of those would have passed TAS-3 (achieve elimination). However, under a conservative assumption that 3-5% of planned surveys can fail, these figures decline to 454 (77.9%) EUs stopping MAM and 284 achieving elimination. For MMDP, Lymphoedema was reported in 450 LGAs, totaling 7,824 cases, with 5,828 (74.5%) actively managed. Hydrocele was reported across 503 LGAs, with 22,195 documented cases, 72.4% of which received management.

Conclusion

Nigeria has made substantial progress toward the elimination of lymphatic filariasis. The overall trajectory suggests that Nigeria remains on course to stop MAM and achieve elimination in 85% and 53% of all endemic IUs, respectively

Authors summary

Lymphatic filariasis (LF) is a mosquito-borne disease that can cause severe and lifelong disability, including limb swelling and scrotal enlargement. Nigeria bears the largest LF burden in Africa,

making progress in the country critical to achieving global elimination. We reviewed 24 years (2000–2024) of national programme data to assess Nigeria's progress toward the 2030 elimination target. Our analysis shows that Nigeria has achieved sustained treatment coverage, made substantial progress in interrupting transmission, and expanded access to quality care for people already affected by LF. Under conservative assumptions, projections indicate that most endemic areas could reach elimination if planned activities continue. However, realizing this potential will require sustained political commitment and increased, targeted financial and partner support to ensure timely surveys, strengthen surveillance, and scale up morbidity management and disability prevention services

Keywords: Lymphatic filariasis elimination; Mass drug administration (MAM); Transmission assessment surveys (TAS); Nigeria; Neglected tropical diseases (NTDs)

Introduction

Lymphatic filariasis (LF) is one of the most dreadful neglected tropical diseases (NTDs). The disease is caused by parasitic filarial nematodes; *Wuchereria bancrofti*, *Brugia Malayi*, or *Brugia timori*, and are transmitted by various mosquito genera, including Anopheles, Culex, and Aedes [1,2]. Infection may be acquired during childhood and deteriorate over the years leading to a chronic damage to the lymphatic system [1]. The disease may result in tissue swelling (lymphoedema), skin thickening (elephantiasis), or scrotal enlargement (hydrocoele) which are painful and profoundly disfiguring [1-3]. Lymphatic filariasis morbidity have been implicated in individuals' quality of life and economic productivity due to the interference with the infected individual's physical and psychological health [1-3]. With over 1 billion people at risk of infections across 80 countries and territories, WHO (1997) classified LF as a public health priority [4]. The

establishment of the Global Programme to Eliminate Lymphatic Filariasis (GPELF) in 2000 aimed at steering intervention activities towards realizing the elimination target [4].

The GPELF has a dual mandate, to interrupt transmission in areas where endemicity is greater than or equal to 1% and to manage morbidity and prevent disability among those affected [5,6]. Transmission interruption relies primarily on preventive chemotherapy, which involves large-scale, annual administration of a single dose of albendazole, combined with either diethylcarbamazine (or ivermectin is used where LF is co-endemic with onchocerciasis) [1,2,5,6]. This strategy is widely referred to as mass administration of medicines (MAM) [1]. Disease control programs are expected to implement a minimum of five rounds of effective MAM annually [7], which is defined as reaching the expected geographic and therapeutic coverage. Geographic coverage is the number of endemic areas by MAM served, while therapeutic coverage is the number of at-risk persons in a defined area that swallowed the distributed medicines [7]. Geographic coverage of 100% and a therapeutic coverage of 65% are necessary to achieve an estimated 99% reduction in prevalence for parasite transmission interruption [8,9]. For morbidity management and disability prevention (MMDP), the GPELF encourages identification of cases, and promotion of hygiene services and training on daily washing, exercise and skin care for individuals with lymphedema and surgical interventions for those with hydrocoele to enhance quality of life and mitigate the long- term impact of the disease [10,11,12].

The GPELF program was adopted globally and implemented through successive roadmaps [2], with the latest outlining ambitious targets that all 72 countries with a history of LF should have

stopped MAM by 2030, with 80% of endemic countries i.e., 58 countries should have be certified to have eliminated LF as a public health problem by 2030 [2]. More than 10 billion cumulative treatments have been delivered during MAM campaigns to 943.4 million people around the world since 2000 [13], and the number of people requiring MAM has significantly declined by half (485.4 million now in need of MAM) [13]. As at 2024, of all 72 endemic countries, 1 (Gabon) was yet to commence MAM, four other countries had started MAM but not at scale to all endemic areas, 30 (Nigeria inclusive) had started MAM across all endemic areas, and 37 had met the target and no longer require MAM [13]. Sixteen of the latter group are under post treatment surveillance (PTS) but had not been validated and 21 countries have met the criteria for elimination of LF as a public health problem and are in the post-validation surveillance (PVS) phase [13].

Despite the progress made globally, there are still significant gaps as 35 of 72 (~49%) countries are yet to stop MAM, and 37 (~64%) are yet to eliminate LF transmission [13]. Most of the endemic countries (34 of 72 countries) are in the African region, and only 15 of those have stopped MAM, and just 2 of them (Togo and Malawi) have successfully eliminated LF transmission [13]. Therefore, achieving the global elimination targets will require an additional 19 (~54% of global target) and 25 (~68% of global target) African countries to discontinue MAM by 2030, and elimination LF transmission, respectively. It is thus important that African countries intensify efforts as the mandate of achieving these targets would heavily be shaped by results from the region. Nigeria bears the largest burden of LF in Africa and is the third most endemic country globally [14-17]. Of the estimated 50 million people affected in sub-Saharan Africa, more than 29 million reside in Nigeria [15,17,18]. Therefore, a critical appraisal of national programmatic

activities is essential for understanding progress toward LF elimination and assessing the feasibility of achieving the 2030 elimination targets. Accordingly, this study synthesizes and reviews the implementation of LF elimination efforts in Nigeria, documents progress made toward the 2030 goals and highlights persistent operational and programmatic challenges that require urgent attention to sustain and accelerate elimination efforts.

Methods

Study design

This review and analysis were undertaken as part of a technical advisory role to the Nigerian National Lymphatic Filariasis Elimination Programme (NLFEP) Expert Committee. The study involved a retrospective analysis of routinely collected programmatic data generated by the NLFEP between 2000 and 2025, complemented by secondary analysis of population-based data obtained from the 2025 Nigeria Demographic and Health Survey (NDHS). The programmatic data were used to assess progress along the lymphatic filariasis elimination pathway, while the NDHS data provided contextual information on coverage and usage of Insecticide-treated net (ITN).

Extraction of study indicators

The programmatic data extracted from the NLFEP database included endemicity classification, treatment history, MAM implementation and coverage, MMDP activities, epidemiological assessment findings, and planned assessment activities for all 774 implementation units (IUs)/local government areas (LGAs) in Nigeria (see S1 Dataset and S2 Dataset 2).

For MMDP, both quantitative datasets and qualitative programmatic reports relating to health-system strengthening activities, including capacity development and health-facility readiness assessments, were reviewed and summarized. Additionally, available data on lymphoedema and hydrocoele cases identified and managed between 2021 and 2025 were extracted from national program reports.

Although vector control activities are not currently included within the core implementation framework of the NLFEP, relevant indicators on ITN ownership, access, and utilization were extracted from the most recent Nigeria Demographic and Health Survey (NDHS) 2023–24 report [19]. Variables of interest included the proportion of households owning at least one ITN, the average number of ITNs per household, the proportion of households with at least one ITN for every two household members, and the proportion of children under five years of age and pregnant women who slept under an ITN the night preceding the survey. These indicators were used to provide contextual information on malaria vector-control coverage and its potential relevance to lymphatic filariasis transmission dynamics.

Data management and analysis

For MAM data, all LF-endemic LGAs in each of the six Nigeria's geopolitical zones were identified from program's inception and their treatment progress was assessed by quantifying the number of effective MAM rounds that have been conducted. The LGAs were classified as either having received fewer than five effective rounds or having completed five or more effective rounds, which is the minimum threshold required before conducting EMS. Following that, the

transmission status of each LGA was assessed using results from the four epidemiological assessments (EMS and TAS 1–3), and LGAs were classified as having passed any of these surveys.

Furthermore, the list of all past and planned epidemiological assessments were extracted to estimate historical failure rate and forecast the feasibility of achieving LF elimination targets by the 2030 endline. Projections were conducted at the geopolitical zone level and were based on LGA-specific starting assessment stage and calendar year. In line with WHO guidance, a two-year interval between successive assessment stages was assumed. The LGAs passing EMS were projected to undergo TAS-1 after two years; those passing TAS-1 were projected to undergo TAS-2 after a further two years; and those passing TAS-2 were projected to undergo TAS-3 after an additional two years. The TAS-3 was treated as the terminal elimination milestone, with no further progression beyond this stage. Projections were extended through 2032 to identify LGAs whose progression would spill beyond the 2030 elimination endline. Trajectory plots were constructed to visualize projected progression across assessment stages over time, with diagonal paths representing biennial advancement and point sizes proportional to the number of LGAs.

Finally, the feasibility of meeting elimination targets was assessed under the conditions that all planned assessments were successful (i.e., not limited by funding, or security challenges, and all LGAs passed the survey), or unsuccessful (i.e. about 3-5% of the LGAs fail the assessment, or assessment were impacted by security and funding). Results were summarized using stacked bar charts by geopolitical zone for each failure-rate scenario.

For MMDP and NDHS data, indicators were summarized using descriptive statistics such as frequencies and percentage across the states and geopolitical zones, and where applicable visualizations were done using maps and line graphs. All data management, analyses, projections, and visualizations were conducted using R software (version 4.5.2) and ArcGIS software (version 10.8).

Data presentation

The results are organized by key programmatic phases of the NLFEP. For each phase, relevant contextual and implementation information is first presented to provide background on the intervention, its objectives, implementation requirements, and operational considerations. This is subsequently followed by the presentation of quantitative findings derived from the program data. This approach was adopted to facilitate interpretation of the results within the broader context of programme implementation and progress toward lymphatic filariasis elimination in Nigeria.

Results

Overview of Nigeria's landscape and Lymphatic Filariasis Intervention Strategy

Nigeria is arguably one of the largest and most populous country in Africa, with approximately 923,770 km² land mass and over 230 million people. [20]. Given its large landmass and population, Nigeria is administratively organized into six geopolitical zones comprising 36 states and the Federal Capital Territory [21]. These are further subdivided into 774 LGAs, which serve as the primary IUs for LF control [21]. The strategy employed in Nigeria follows the World Health Organization–recommended comprehensive elimination strategy, which comprises both interruption of transmission and MMDP [1,11], and is implemented through a constituted NLFEP.

The NLFEP was established in 1997 and operated through a multi-level governance structure that promoted synergies across the workforce at national and state levels, supported by a national steering committee responsible for technical oversight. Implementation of MAM and MMDP services leveraged the existing NTDs workforce at both state and implementation-unit levels, operating under national directives and technical guidance provided by NFLEP. Resource mobilization and strategic direction followed a top–down model, while operational implementation—including localization of strategies and adaptation to contextual realities—was decentralized to state and implementation units. Program activities (meetings, trainings, coverage surveys, MAM, MMDP, and epidemiological assessments such as baseline mapping, EMS and TAS 1-3) were largely supported through external funding secured by international and indigenous NGDOs. Although implementation of MAM and MMDP were decentralized to the states, monitoring and evaluation activities, including surveys, EMS and TAS were coordinated centrally by the national program team. This centralized evaluation approach ensures methodological consistency and minimizes potential bias associated with local program ownership. Consequently, evaluation processes functioned as external assessments of program performance at subnational levels. Throughout the program cycle, the NLFEP Expert Committee provided continuous technical advisory support, ensuring alignment with national elimination goals and global LF control strategies.

Baseline Mapping of LF infections in Nigeria

Mapping is the first programmatic step in LF elimination and is used to assess the disease situation in a country and to identify areas in which MAM is required by determining where active transmission was occurring. Mapping of LF in Nigeria was completed between 2000 and 2011

(Table 1). Briefly, it involves a rapid assessment of 50-100 adults (aged 15 years and older) across two high-risk villages selected based on the presence of chronic morbidity, high mosquito density, or other ecological indicators. Blood samples, usually collected by capillary action, were tested for microfilaremia or antigenemia, and decisions to implement MAM were made when prevalence was up ≥1% [22-24]. Although these guidelines were widely used in the early stages of GPELF, concerns have emerged regarding their sensitivity [25], and this has led to the development of a more robust confirmatory mapping method that prioritizes the inclusion of more sampling sites (30 sites against previous two sites), participants (480 children against previous 100 adults) to improve the reliability of mapping and decision-making [25]. The NFLEP has conducted confirmatory mapping in some areas classified as non-endemic [26], with findings in consonance with previous mapping results.

Overall, Nigeria achieved complete mapping coverage of all 774 LGAs, representing the entire national population of over 213 million people. Of these, 583 (75.3%) were endemic for LF (Fig 1). Endemicity varied across the six geopolitical zones in the country (Table 1), with the heaviest burden (>90% of all IUs) in the Northwest and Southeast zones, while the South-South had the lowest prevalence at 52%. In the Southeast zone, 95 LGAs across the five states were mapped between 2005 and 2011, with an overall endemicity of 92.6%. Three states in the zone (Abia, Anambra, and Imo) had 100% endemicity. In the Southwest, 137 LGAs were mapped across six states between 2003 to 2013, and 65.7% were endemic. Endemicity ranged between 30-94%, with the lowest in Lagos and Oyo. In the South-south, mapping was completed between 2003 and 2011 for 123 LGAs, with overall endemicity of 52.0%. Bayelsa recorded the highest endemicity with 87.5% of LGAs affected, while Akwa Ibom and Edo showed relatively low levels, at 35.5% and

38.9%, respectively. In the Northeast, 112 LGAs across six states were mapped between 2008 and 2018, with an average endemicity of 68.8%. Gombe had the highest burden (90.9%), followed by Adamawa (85.7%) and Taraba (81.3%). In the Northwest, 186 LGAs were mapped across seven states between 2008 and 2013, with high endemicity of 93.0%. All the LGAs in Kano and Jigawa were endemic. Katsina, Kebbi, Sokoto, Kaduna and Zamfara also had high endemicity exceeding 75%. In the North Central zone, 121 LGAs were mapped between 2000 and 2013, with 75.2% found to be endemic. All the LGAs in Nasarawa and Plateau were endemic. Benue and Niger had two-third of their LGAs endemic, while Kogi, FCT Abuja and Kwara had over half of their LGAs endemic. (Table 1)

Fig 1: Map of Nigeria showing the Baseline endemicity of Lymphatic Filariasis prior to implementation of MAM. *This map was created using programme primary data (See S1 Dataset) and developed in R software (v4.3.3). Shapefiles for Nigeria, including boundary polygons and water bodies across all administrative levels, were sourced from publicly accessible databases on the Humanitarian Data Exchange and are licensed under CC BY 4.0 (https://data.humdata.org/dataset/cod-ab-nga). No changes were made to the original shapefiles. The authors grant permission for the reuse of this map without restriction, provided appropriate credit is given to the source.*

Table 1: Baseline Endemicity Profile of Lymphatic Filariasis in Nigeria as of 2024

**Zone**	**State**	**Population**	**No of LGA**	**No. of LGAs mapped**	**Not Endemic**	**Endemic**	**Date of Mapping**
**Southeast**	Abia	4312266	17	17 (100.0)	0 (0.0)	17 (100.0)	2008
	Anambra	6363454	21	21 (100.0)	0 (0.0)	21 (100.0)	2007-2010
	Ebonyi	3307238	13	13 (100.0)	4 (30.8)	9 (69.2)	2005-2008
	Enugu	4956364	17	17 (100.0)	3 (17.6)	14 (82.4)	2005-2011
	Imo	5987413	27	27 (100.0)	0 (0.0)	27 (100.0)	2007-2010
	Total	24926735	95	95 (100.0)	7 (7.4)	88 (92.6)	2005-2011
**Southwest**	Ekiti	3626956	16	16 (100.0)	0 (0.0)	16 (100.0)	2003-2011
	Lagos	13715153	20	20 (100.0)	14 (70.0)	6 (30.0)	2003-2011
	Ogun	5672743	20	20 (100.0)	6 (30.0)	14 (70.0)	2003-2011
	Ondo	5235923	18	18 (100.0)	1 (5.6)	17 (94.4)	2003-2009
	Osun	5209315	30	30 (100.0)	3 (10.0)	27 (90.0)	2003-2013
	Oyo	8508262	33	33 (100.0)	23 (69.7)	10 (30.3)	2003-2013
	Total	41968352	137	137 (100.0)	47 (34.3)	90 (65.7)	2003-2013

**South-South**	Akwa Ibom	5965062	31	31 (100.0)	20 (64.5)	11 (35.5)	2009-2010
	Bayelsa	2591862	8	8 (100.0)	1 (12.5)	7 (87.5)	2009-2010
	Cross River	4395906	18	18 (100.0)	8 (44.4)	10 (55.6)	2009-2010
	Delta	6236189	25	25 (100.0)	9 (36.0)	16 (64.0)	2003-2011
	Edo	4897071	18	18 (100.0)	11 (61.1)	7 (38.9)	2008-2011
	Rivers	7890201	23	23 (100.0)	10 (43.5)	13 (56.5)	2009-2011
	Total	31976291	123	123 (100.0)	59 (48.0)	64 (52.0)	2003-2011
**Northeast**	Adamawa	4820641	21	21 (100.0)	3 (14.3)	18 (85.7)	2008-2010
	Bauchi	7115669	20	20 (100.0)	9 (45.0)	11 (55.0)	2008-2013
	Borno	6316532	27	27 (100.0)	13 (48.1)	14 (51.9)	2008-2018
	Gombe	3581705	11	11 (100.0)	1 (9.1)	10 (90.9)	2008-2009
	Taraba	3470026	16	16 (100.0)	3 (18.8)	13 (81.3)	2008-2010
	Yobe	3532576	17	17 (100.0)	6 (35.3)	11 (64.7)	2008-2010
	Total	28837149	112	112 (100.0)	35 (31.3)	77 (68.8)	2008-2018
**Northwest**	Jigawa	6616983	27	27 (100.0)	0 (0.0)	27 (100.0)	2008-2010
	Kaduna	9230991	23	23 (100.0)	5 (21.7)	18 (78.3)	2008-2011
	Kano	14278381	44	44 (100.0)	0 (0.0)	44 (100.0)	2008-2010
	Katsina	8814090	34	34 (100.0)	4 (11.8)	30 (88.2)	2008-2013
	Kebbi	4927956	21	21 (100.0)	1 (4.8)	20 (95.2)	2008-2010
	Sokoto	5625422	23	23 (100.0)	3 (13.0)	20 (87.0)	2008-2011
	Zamfara	4960241	14	14 (100.0)	0 (0.0)	14 (100.0)	2008-2010
	Total	54454064	186	186(100.0)	13 (7.0)	173 (93.0)	2008-2013
**North Central**	Benue	6420080	23	23 (100.0)	7 (30.4)	16 (69.6)	2004-2013
	FCT, Abuja	2138179	6	6 (100.0)	2 (33.3)	4 (66.7)	2008-2010
	Kogi	4988605	21	21 (100.0)	10 (47.6)	11 (52.4)	2008-2010
	Kwara	3607893	16	16 (100.0)	5 (31.3)	11 (68.8)	2003-2010
	Nasarawa	2835193	13	13 (100.0)	0 (0.0)	13 (100.0)	2000
	Niger	6010772	25	25 (100.0)	6 (24.0)	19 (76.0)	2008-2011
	Plateau	4836786	17	17 (100.0)	0 (0.0)	17 (100.0)	2000
	Total	30837508	121	121(100.0)	30 (24.8)	91 (75.2)	2000-2013
	**Total**	**213000099**	**774**	**774 (100.0)**	**191 (24.7)**	**583 (75.3)**	**2000-2011**

LGA: Local Government Areas

Mass Administration of Medicines Campaigns for LF control in Nigeria

Following the delineation of endemic LGAs, the next and most resource-intensive phase is mass administration of medicines (MAM), which involves the periodic delivery of antifilarial medicines (ivermectin and albendazole) to at-risk populations within endemic LGAs or implementation units (IUs) [1,2,11]. Each MAM is implemented at the community level and targets all eligible individuals aged ≥5 years, using community-directed distributors (CDDs) who are unpaid volunteers selected by their communities. A typical MAM "round" represents one complete treatment cycle, conducted once yearly [11]. It begins with the training of CDDs, followed by advocacy visits and community sensitization to ensure awareness and acceptance. These preliminary steps are essential for achieving high treatment coverage and maintaining program fidelity. During drug distribution, CDDs record treatments in village registers, which are then collated and submitted to LGA-level NTD coordinators. National and global guidelines recommend achieving absolute geographic coverage i.e., reaching all households and attaining a minimum epidemiological and programmatic coverage thresholds, generally at least 65% of the total population or 80% of the eligible population to sufficiently reduce microfilaria transmission [11]. Sustaining these levels for a minimum of five MAM rounds is expected to achieve a ~99% reduction in prevalence required to interrupt transmission, and only LGAs that meet these requirements are considered eligible for epidemiological assessments to verify whether transmission has been interrupted. At the national level, all 583 endemic LGAs across the country have been reached with MAM since initiation between 2000 and 2015 [13]. By the time of this publication, the number of rounds administered ranged from 3 to 15, and 529 LGAs (90.7%) have achieved the WHO-recommended ≥5 effective rounds. The Southeast and Northwest zones demonstrate exemplary performance with about 9 effective MAM rounds, while gaps remain in the Northeast and parts of the South-South and Southwest. (Fig 2)

Fig 2: Map of Nigeria showing the progress of MAM for Lymphatic Filariasis Control in Nigeria as of 2024. *This map was created using programme primary data (See S1 Dataset) and developed in R software v4.3.3 software. Shapefiles for Nigeria, including boundary polygons and water bodies across all administrative levels, were sourced from publicly accessible databases available at Humanitarian Data Exchange, and are licensed under CC BY 4.0 (https://data.humdata.org/dataset/cod-ab-nga). No changes were made to the original shapefiles. The authors grant permission for the reuse of this map without restriction, provided appropriate credit is given to the source.*

The progress of MAM varied across the six-geopolitical regions in the country**.** In the Southeast, all 88 endemic LGAs have already achieved at least five effective MAM rounds. This marks the Southeast as one of the strongest performing zones nationally. In the Southwest, the number of effective MAM rounds varied from as few as 3 rounds in Ogun to as many as 15 rounds in Osun. About 87% of LGAs have had ≥5 effective MAM, mostly from Ekiti, Ondo, Osun, and Oyo. However, challenges remain in Ogun and Lagos. In the South-South, all 64 endemic LGAs were reached with MAM between 2010 and 2015. While most states have all LGAs meeting the effective threshold, Rivers State stand out as a concern, with 84.6% of their LGAs still below 5 effective rounds. In the Northeast, MAM was initiated between 2009 and 2019 across all endemic LGAs. (Table 2)

The implementation history reveals considerable variation, with states like Yobe and Taraba implementing between 10 and 13 rounds in most LGAs, Borno only started MAM in 2019, with none of its LGAs meeting the required ≥5 effective rounds of MAM. Hence only 72.7% of LGAs in the zone have achieved ≥5 effective rounds. In the Northwest zone, nearly all LGAs (97.1%) have achieved at least five effective rounds, except in Kano and Katsina. This zone stands out as one of the most successful in terms of scale and effectiveness of MAM implementation. In North

Central, all 91 endemic LGAs across seven states were reached with MAM beginning as early as 2000 in Nasarawa and Niger, and later in others like Benue and Plateau. The number of treatment rounds ranged from 5 to 13. Overall, 95.6% of LGAs have completed ≥5 effective rounds. The Federal Capital Territory (FCT) was somewhat unique, with half of its LGAs still under 5 rounds despite an early start in 2010. Otherwise, most states in this zone have shown consistent progress, placing North Central among the stronger performing zones. (Table 2)

Table 2: Lymphatic Filariasis MAM progress in Nigeria as of 2024

Zone	State	Number of endemic LGAs	LGAs experiencing MAM	Year of Initiation	MAM rounds completed	Effective MAM rounds < 5	Effective MAM rounds ≥5
**South East**	Abia	17	17 (100.0)	2014	7-8	0 (0.0)	17 (100.0)
	Anambra	21	21 (100.0)	2014	6-8	0 (0.0)	21 (100.0)
	Ebonyi	9	9 (100.0)	2014	6-9	0 (0.0)	9 (100.0)
	Enugu	14	14 (100.0)	2014	7-9	0 (0.0)	14 (100.0)
	Imo	27	27 (100.0)	2014	6-8	0 (0.0)	27 (100.0)
	Total	88	88 (100.0)	2014	6-9	0 (0.0)	88 (100.0)
**Southwest**	Ekiti	16	16 (100.0)	2008	5-13	0 (0.0)	16 (100.0)
	Lagos	6	6 (100.0)	2020	11-14	6 (100.0)	0 (0.0)
	Ogun	14	14 (100.0)	2014	3	4 (28.6)	10 (71.4)
	Ondo	17	17 (100.0)	2008	6-9	0 (0.0)	17 (100.0)
	Osun	27	27 (100.0)	2008	10-15	0 (0.0)	27 (100.0)
	Oyo	10	10 (100.0)	2015	5-13	2 (20.0)	8 (80.0)
	Total	90	90 (100.0)	2008-2015<br>2014	3-15	12 (13.3)	78 (86.7)
**South South**	Akwa Ibom	11	11 (100.0)		7-8	0 (0.0)	11 (100.0)
	Bayelsa	7	7 (100.0)	2011	4-7	1 (14.3)	6 (85.7)
	Cross River	10	10 (100.0)	2010	8-12	0 (0.0)	10 (100.0)
	Delta	16	16 (100.0)	2014	7-9	2 (12.5)	14 (87.5)
	Edo	7	7 (100.0)	2014	7-10	0 (0.0)	7 (100.0)
	Rivers	13	13 (100.0)	2015	4-7	11 (84.6)	2 (15.4)
	Total	64	64 (100.0)	2010-2015	4-12	14 (21.9)	50 (78.1)
**Northeast**	Adamawa	18	18 (100.0)	2012	4-9	2 (11.1)	16 (88.9)
	Bauchi	11	11 (100.0)	2012	7-11	0 (0.0)	11 (100.0)
	Borno	14	14 (100.0)	2019	1-4	14 (100.0)	0 (0.0)
	Gombe	10	10 (100.0)	2009	7-9	1 (10.0)	9 (90.0)

	Taraba	13	13 (100.0)	2009	8-13	1 (7.7)	12 (92.3)
	Yobe	11	11 (100.0)	2011	10-11	3 (27.3)	8 (72.7)
	Total	77	77 (100.0)	2009-2012	1-13	21 (27.3)	56 (72.7)
**Northwest**	Jigawa	27	27 (100.0)	2011	8-11	0 (0.0)	27 (100.0)
	Kaduna	18	18 (100.0)	2010	6-12	0 (0.0)	18 (100.0)
	Kano	44	44 (100.0)	2010	7-11	1 (2.3)	43 (97.7)
	Katsina	30	30 (100.0)	2015	7-11	4 (13.3)	26 (86.7)
	Kebbi	20	20 (100.0)	2010	7-8	0 (0.0)	20 (100.0)
	Sokoto	20	20 (100.0)	2011	6-14	0 (0.0)	20 (100.0)
	Zamfara	14	14 (100.0)	2010	6-13	0 (0.0)	14 (100.0)
	Total	173	173 (100.0)	2010-2015	6-14	5 (2.9)	168 (97.1)
**North Central**	Benue	16	16 (100.0)	2012	6-9	2 (12.5)	14 (87.5)
	FCT, Abuja	4	4 (100.0)	2010	9-11	0 (0.0)	4 (100.0)
	Kogi	11	11 (100.0)	2010	5-11	0 (0.0)	11 (100.0)
	Kwara	11	11 (100.0)	2010	5-11	0 (0.0)	11 (100.0)
	Nasaraw	13	13 (100.0)	2000	8-13	0 (0.0)	13 (100.0)
	Niger	19	19 (100.0)	2000	8-13	0 (0.0)	19 (100.0)
	Plateau	17	17 (100.0)	2011	7-13	0 (0.0)	17 (100.0)
	Total	91	91 (100.0)	2000-2012	5-13	2 (2.2)	89 (97.8)
**Total**		583	583 (100.0)	2000-2015	3-15	54 (9.3)	529 (90.7)

LGA: Local Government Areas

Epidemiological assessments to ascertain LF transmission status in Nigeria

Following mapping and the effective implementation of at least five rounds of MAM with ivermectin and albendazole in endemic areas, evaluation units are required to demonstrate that infection levels have been reduced to the point where transmission is unlikely i.e., <2% for antigenemia or <1% for microfilariae (Fig 3). These assessments is coordinated by the NLFEP and involves four sequential assessments, the first of which is one epidemiological monitoring surveys (EMS) to determine whether MAM can be stopped, and three transmission assessment surveys (TAS) to confirm sustained interruption of transmission [11]. These assessments are conducted at the evaluation units (EUs), which are programmatically defined geographic area comprising one or more contiguous LGAs/IUs, typically with similar epidemiological characteristics, treatment

histories, and population sizes within WHO-recommended threshold (~ population not exceeding 2 million) [11]. The EUs are formed to ensure feasibility of assessment and the resulting outcomes are applied to, and inherited by, all IUs within the EU for programmatic decision-making [11].

During the EMS, parasitological and serological assessments are carried out in two purposively selected sites (sentinel and spot-check sites) within an EU, typically among children aged 5 years and above [27]. However, the revised WHO guidelines now stipulate that eligibility should be assessed among individuals aged 19 years and older [28]. An EU qualifies for first TAS (i.e., TAS-1) if seroprevalence is below 1% for microfilaria or below 2% for antigenemia. If these thresholds are not met, the EU is advised to implement two additional rounds of effective MAM before repeating the EMS [28]. In the second step, EUs that pass EMS advance to the TAS-1, which is conducted among children aged 6-7 years and determines eligibility to stop MAM. Under previous guidance, EUs were judged to be successful if seroprevalence was <2% for antigenemia or <1% for microfilariae; however, the revised guidance requires <1% for both. In this report, TAS-1 was conducted before the updated criteria and evaluated according to the earlier thresholds. The EUs that fails TAS-1 are required to undergo two additional rounds of effective MAM before repeating the evaluation, while those that pass are allowed to stop MAM and enter the post-treatment surveillance (PTS) phase [22]. The PTS phase comprises of two subsequent surveys, TAS-2 and TAS-3, conducted at intervals of 2–3 years after stopping MAM. Successful passage through these stages allows the country to progress to the validation phase, where all EUs must have passed TAS-3 to be considered eligible for validation of LF transmission interruption.

Of the 583 endemic LGAs, 529 (90.7%) have completed at least five effective rounds of MAM, representing substantial progress toward eligibility for EMS (Fig. 3). Overall, 445 endemic LGAs (76.3%) have passed EMS and 84 (14.4%) are awaiting EMS. Furthermore, 346 (59.3%) have successfully passed TAS-1 and stopped MAM. Progression beyond TAS-1 remains limited nationally, with only 40 endemic LGAs (6.9%) having passed TAS-2 and 30 LGAs (5.1%) having passed TAS-3 (Table 3).

Marked geographical variation in transmission status was observed across the six geopolitical zones. In the South-East zone, all 88 endemic LGAs completed at least five effective MAM rounds and passed EMS (100%), while 84 (95.5%) passed TAS-1 and stopped MAM, indicating strong programmatic performance in the zone. These LGAs are currently awaiting progression to TAS-2. In the Southwest zone, 78 of 90 endemic LGAs (86.7%) completed at least five effective MAM rounds, 66 LGAs (73.3%) passed EMS, and 51 LGAs (56.7%) passed TAS-1 and stopped MAM. However, substantial gaps remain, particularly in Lagos State, where none of the endemic LGAs had progressed to EMS, and in Ogun State, where 10 LGAs (71.4%) had completed effective MAM but were yet to undergo EMS.

In the South South zone, 50 of 64 endemic LGAs (78.1%) completed at least five effective MAM rounds, 39 (60.9%) passed EMS, and 38 (59.4%) passed TAS-1, while 8 (12.5%) progressed to TAS-2. Performance varied widely within the zone, with Rivers and Bayelsa States exhibiting substantial challenges related to low MAM coverage and limited advancement through assessment surveys. In the North East zone, 56 of 77 endemic LGAs (72.7%) completed at least five effective MAM rounds, 43 (55.8%) passed EMS, and 28 (36.4%) passed TAS-1, with none yet progressing

to TAS-2. Similarly, in the North West zone, although 168 of 173 endemic LGAs (97.1%) completed at least five effective MAM rounds, only 131 LGAs (75.7%) passed EMS and 77 LGAs (44.5%) passed TAS-1, highlighting slower progression toward transmission interruption despite high MAM coverage.

The North Central zone demonstrated the most advanced progress nationally. Of the 91 endemic LGAs, 89 (97.8%) completed at least five effective MAM rounds, 78 LGAs (85.7%) passed EMS, and 64 LGAs (70.3%) passed TAS-1. Notably, 32 LGAs (35.2%) passed TAS-2 and 30 LGAs (33.0%) passed TAS-3, confirming sustained interruption of transmission in a substantial proportion of endemic LGAs in the zone. States such as Nasarawa and Plateau achieved complete progression through TAS-3, reflecting exceptional programmatic performance and serving as potential models for other endemic areas (Table 3).

Fig 3: Map of Nigeria showing the transmission status of Lymphatic Filariasis ~~in Nigeria~~ as of 2024. *This map was created using primary data collected by the authors (S1 Dataset) and developed in R software v4.3.3 software. Shapefiles for Nigeria, including boundary polygons and water bodies across all administrative levels, were sourced from publicly accessible databases available at Humanitarian Data Exchange, and are licensed under CC BY 4.0 (https://data.humdata.org/dataset/cod-ab-nga). No changes were made to the original shapefiles. The authors grant permission for the reuse of this map without restriction, provided appropriate credit is given to the source.*

Table 3: Transmission Status of Lymphatic Filariasis in Nigeria as of 2024

Zone	State	No. of endemic LGAs	No. of EUs with at least 5 effective MAM	No. of LGAs that has passed EMS	No. of LGAs that has passed TAS-1	No. of LGAs that has passed TAS-2	No. of LGAs that has passed TAS-3
**South East**	Abia	17	17 (100.0)	17 (100.0)	17 (100.0)	0 (0.0)	0 (0.0)
	Anambra	21	21 (100.0)	21 (100.0)	21 (100.0)	0 (0.0)	0 (0.0)

	Ebonyi	9	9 (100.0)	9 (100.0)	9 (100.0)	0 (0.0)	0 (0.0)
	Enugu	14	14 (100.0)	14 (100.0)	14 (100.0)	0 (0.0)	0 (0.0)
	Imo	27	27 (100.0)	27 (100.0)	23 (85.2)	0 (0.0)	0 (0.0)
	Total	88	88 (100.0)	88 (100.0)	84 (95.5)	0 (0.0)	0 (0.0)
**Southwest**	Ekiti	16	16 (100.0)	16 (100.0)	16 (100.0)	0 (0.0)	0 (0.0)
	Lagos	6	0 (0.0)	0 (0.0)	0 (0.0)	0 (0.0)	0 (0.0)
	Ogun	14	10 (71.4)	0 (0.0)	0 (0.0)	0 (0.0)	0 (0.0)
	Ondo	17	17 (100.0)	17 (100.0)	17 (100.0)	0 (0.0)	0 (0.0)
	Osun	27	27 (100.0)	27 (100.0)	15 (55.6)	0 (0.0)	0 (0.0)
	Oyo	10	8 (80.0)	6 (60.0)	3 (30.0)	0 (0.0)	0 (0.0)
	Total	90	78 (86.7)	66 (73.3)	51 (56.7)	0 (0.0)	0 (0.0)
**South South**	Akwa Ibom	11	11 (100.0)	7 (63.6)	6 (54.5)	0 (0.0)	0 (0.0)
	Bayelsa	7	6 (85.7)	1 (14.3)	1 (14.3)	0 (0.0)	0 (0.0)
	Cross River	10	10 (100.0)	10 (100.0)	10 (100.0)	8 (80.0)	0 (0.0)
	Delta	16	14 (87.5)	14 (87.5)	14 (87.5)	0 (0.0)	0 (0.0)
	Edo	7	7 (100.0)	7 (100.0)	7 (100.0)	0 (0.0)	0 (0.0)
	Rivers	13	2 (15.4)	0 (0.0)	0 (0.0)	0 (0.0)	0 (0.0)
	Total	64	50 (78.1)	39 (60.9)	38 (59.4)	8 (12.5)	0 (0.0)
**Northeast**	Adamawa	18	16 (88.9)	7 (38.9)	0 (0.0)	0 (0.0)	0 (0.0)
	Bauchi	11	11 (100.0)	9 (81.8)	7 (63.6)	0 (0.0)	0 (0.0)
	Borno	14	0 (0.0)	0 (0.0)	0 (0.0)	0 (0.0)	0 (0.0)
	Gombe	10	9 (90.0)	9 (90.0)	8 (80.0)	0 (0.0)	0 (0.0)
	Taraba	13	12 (92.3)	10 (76.9)	5 (38.5)	0 (0.0)	0 (0.0)
	Yobe	11	8 (72.7)	8 (72.7)	8 (72.7)	0 (0.0)	0 (0.0)
	Total	77	56 (72.7)	43 (55.8)	28 (36.4)	0 (0.0)	0 (0.0)
**Northwest**	Jigawa	27	27 (100.0)	24 (88.9)	21 (77.8)	0 (0.0)	0 (0.0)
	Kaduna	18	18 (100.0)	15 (83.3)	9 (50.0)	0 (0.0)	0 (0.0)
	Kano	44	43 (97.7)	40 (90.9)	17 (38.6)	0 (0.0)	0 (0.0)
	Katsina	30	26 (86.7)	14 (46.7)	0 (0.0)	0 (0.0)	0 (0.0)
	Kebbi	20	20 (100.0)	20 (100.0)	14 (70.0)	0 (0.0)	0 (0.0)
	Sokoto	20	20 (100.0)	18 (90.0)	16 (80.0)	0 (0.0)	0 (0.0)
	Zamfara	14	14 (100.0)	0 (0.0)	0 (0.0)	0 (0.0)	0 (0.0)
	Total	173	168 (97.1)	131 (75.7)	77 (44.5)	0 (0.0)	0 (0.0)
**North Central**	Benue	16	14 (87.5)	3 (18.8)	3 (18.8)	0 (0.0)	0 (0.0)
	FCT, Abuja	4	4 (100.0)	4 (100.0)	2 (50.0)	2 (50.0)	0 (0.0)
	Kogi	11	11 (100.0)	11 (100.0)	9 (81.8)	0 (0.0)	0 (0.0)
	Kwara	11	11 (100.0)	11 (100.0)	10 (90.9)	0 (0.0)	0 (0.0)
	Nasarawa	13	13 (100.0)	13 (100.0)	13 (100.0)	13 (100.0)	13 (100.0)
	Niger	19	19 (100.0)	19 (100.0)	10 (52.6)	0 (0.0)	0 (0.0)
	Plateau	17	17 (100.0)	17 (100.0)	17 (100.0)	17 (100.0)	17 (100.0)
	Total	91	89 (97.8)	78 (85.7)	64 (70.3)	32 (35.2)	30 (33.0)

**Total**	583	529 (90.7)	443 (76.3)	346 (59.3)	40 (6.9)	30 (5.1)

LGAs: Local Government Areas; EMS: Epidemiological Monitoring Survey ; MAM: Mass Administration of Medicines; TAS: Transmission Assessment Surveys; Percentages are calculated using the number of endemic evaluation units (EUs) as the denominator Eligibility-based activities such as EMS and TAS are conducted only in EUs that meet programmatic criteria (e.g., ≥5 effective MAM rounds) but are presented here to summarize overall progress among endemic EUs;

Profile of States with limited progress in the LF Elimination program in each zone

The least-performing states within each geopolitical zone were identified and profiled. Overall, progress toward LF elimination remained limited in six states: Katsina, Zamfara, Borno, Rivers, Ogun, and Lagos. (Table 4). Katsina had relatively strong programmatic performance, with over 85% of endemic LGAs completing effective MAM and approximately 47% progressing to and passing EMS. Zamfara achieved full completion of effective MAM across endemic LGAs; however, none had progressed to EMS (Table 4). None of the LGAs in Borno and Lagos have had 5 effective MAM rounds. Ogun and Rivers State showed moderate MAM completion but had not advanced to EMS (Table 4). Programme records indicated that progress in the northern states was affected by persistent insecurity, while implementation challenges in parts of the southern states were associated with funding constraints, limited partner support, and reduced donor engagement (Table 4).

Table 4: Profile of States with limited progress in the LF Elimination program

State	Number of endemic LGAs	% of LGA with effective MAM completed	% of LGAs that have passed EMS	Challenges
Katsina	26	86.7	47	Insecurity
Zamfara	14	100	0	Insecurity
Borno	14	0	0	Insecurity
Rivers	13	15.4	0	Insecurity
Ogun	14	71.4	0	Funding, lack of partners and donors
Lagos	6	0	0	Funding

LGAs: Local Government Areas; MAM: Mass Administration of Medicines; EMS: Epidemiological Monitoring Survey

Planned Transmission Assessment Surveys in Nigeria (2024–2027)

At the national level, a total of 499 LGAs are expected to undergo different assessment between 2024 and 2027 as part of the elimination strategy. In 2024, 135 LGAs (27.2%) were expected to undergo either EMS or TAS, increasing to 173 LGAs (34.8%) in 2025, 123 LGAs (24.6%) in 2026 and 68 LGAs (13.6%) in 2027. (Table 5).

Table 5: Planned Assessment in Nigeria (2024–2027)

		Number of LGAs				
**Zone**	**Assessment Stage**	**Total**	**2024**	**2025**	**2026**	**2027**
**South East**	EMS	0(0)	0(0)	0(0)	0(0)	0(0)
	TAS-1	4 (4.5)	0 (0.0)	0 (0.0)	4 (100.0)	0 (0.0)
	TAS-2	84 (95.5)	15 (17.9)	45 (53.6)	13 (15.5)	11 (13.1)
	TAS 3	0(0)	0(0)	0(0)	0(0)	0(0)
	Total	88 (100.0)	15 (17.0)	45 (51.1)	17 (19.3)	11 (12.5)
**Southwest**	EMS	12 (15.4)	12 (100.0)	0 (0.0)	0 (0.0)	0 (0.0)
	TAS-1	17 (21.8)	2 (11.8)	15 (88.2)	0 (0.0)	0 (0.0)
	TAS-2	49 (62.8)	0 (0.0)	10 (20.4)	22 (44.9)	17 (34.7)
	TAS 3	0(0)	0(0)	0(0)	0(0)	0(0)
	Total	78 (100.0)	14 (18.0)	25 (32.1)	22 (28.2)	17 (21.8)
**South South**	EMS	11 (22.0)	7 (63.6)	4 (36.4)	0 (0.0)	0 (0.0)
	TAS-1	1 (2.0)	1 (100.0)	0 (0.0)	0 (0.0)	0 (0.0)
	TAS-2	30 (60.0)	1 (3.3)	1 (3.3)	15 (50.0)	13 (43.3)
	TAS 3	8 (16.0)	0 (0.0)	5 (62.5)	3 (37.5)	0 (0.0)
	Total	50(100.0)	9 (18.0)	10 (20.0)	18 (36.0)	13 (26.0)
**Northeast**	EMS	14 (25.0)	1 (7.1)	13 (92.9)	0 (0.0)	0 (0.0)
	TAS-1	15 (26.8)	11 (73.3)	3 (20.0)	0 (0.0)	1 (6.7)
	TAS-2	27 (48.2)	0 (0.0)	14 (51.9)	7 (25.9)	6 (22.2)
	TAS 3	0 (0)	0 (0)	0 (0)	0 (0)	0 (0)
	Total	56 (100.0)	12 (21.4)	30 (53.6)	7 (12.5)	7 (12.5)

**Northwest**	EMS	39 (23.2)	28 (75.7)	11 (24.3)	0 (0.0)	0 (0.0)
	TAS-1	52 (30.9)	36 (69.2)	16 (30.8)	0 (0.0)	0 (0.0)
	TAS-2	77 (45.8)	12 (15.6)	10 (13.0)	45 (58.4)	10 (13.0)
	TAS 3	0 (0)	0 (0)	0 (0)	0 (0)	0 (0)
	Total	168	76 (45.3)	37 (22.0)	45 (26.8)	10 (6.0)
**North Central**	EMS	12 (20.3)	3 (10.0)	9 (90.0)	0 (0.0)	0 (0.0)
	TAS-1	11 (18.6)	6 (55.5)	5 (45.5)	0 (0.0)	0 (0.0)
	TAS-2	34 (57.6)	0 (0.0)	12 (35.3)	12 (35.3)	10 (29.4)
	TAS 3	2 (3.4)	0 (0.0)	0 (0.0)	2 (100.0)	0 (0.0)
	Total	59 (100)	9 (15.8)	26 (45.6)	12 (21.1)	10 (17.5)
**Total**		499 (100)	135 (27.2)	173 (34.8)	123 (24.6)	68 (13.6)

LGAs: Local Government Areas; EMS: Epidemiological Monitoring Survey; MAM: Mass Administration of Medicines; TAS: Transmission Assessment Surveys

In the Southeast Zone, 88 LGAs are planned for epidemiological assessments, with 84 (95.5%) scheduled for TAS-2 and 4 (4.5%) for TAS-1. The TAS-2 assessments are distributed across 15 EUs in 2024, 45 in 2025, 13 in 2026, and 11 in 2027. All four TAS-1 assessments are scheduled for 2026. (Table 5). Similarly, in the Southwest zone, 78 LGAs are scheduled for assessments between 2024 and 2027 including 12 (15.4%) of the LGAs planning for EMS, 17 (21.8%) for TAS-1, and 49 (62.8%) for TAS-2. All EMS are scheduled for 2024, most TAS-1 assessments are planned for 2025, and TAS-2 assessments are distributed between 2025 and 2027 (Table 5).In the South-South Zone, 50 LGAs are scheduled for evaluation surveys between 2024 and 2027, including 11 (22.0%) LGAs planned for EMS, 1 (2.0%) for TAS-1, 30 (60.0%) for TAS-2, and 8 (16.0%) for TAS-3. EMS activities are scheduled for 2024 and 2025, TAS-1 for 2024, TAS-2 across all four years, and TAS-3 for 2025 and 2026 (Table 5).

In the Northeast Zone, 56 LGAs are scheduled for evaluation surveys between 2024 and 2027, including 14 (25.0%) for EMS, 15 (26.8%) for TAS-1, and 27 (48.2%) for TAS-2. Most EMS

assessments (92.9%) are planned for 2025, while TAS-1 assessments (93.3%) are scheduled for 2024 and 2025. TAS-2 assessments are distributed between 2025 and 2027 (Table 5).

In the Northwest Zone, 168 LGAs are scheduled for evaluation surveys between 2024 and 2027, including 39 (23.2%) for EMS, 52 (30.9%) for TAS-1, and 77 (45.8%) for TAS-2. Most EMS activities (75.7%) are planned for 2024, while TAS-1 assessments are scheduled for 2024 (69.2%) and 2025 (30.8%). TAS-2 assessments are distributed across 2024–2027 (Table 5). Similarly, in the North-Central Zone, 57 LGAs are scheduled for evaluation surveys between 2024 and 2027, including 12 (21.1%) for EMS, 11 (19.3%) for TAS-1, 32 (56.1%) for TAS-2, and 2 (3.6%) for TAS-3. EMS activities are primarily planned for 2025, TAS-1 assessments for 2024 (55.5%) and 2025, TAS-2 assessments for 2025–2027, and TAS-3 assessments for 2026 (Table 5).

Historical failure rate for EMS across the country

Across the six geopolitical zones, only 18 (3.4%) of the 529 assessed LGAs have ever failed EMS. Evidence of programme failure under these scenarios was limited geographically to only 11 of the 37 states. In the South East zone, failures were observed in Anambra, Enugu, and Imo States (one LGAs each), and Ebonyi State (three LGAs). In the Southwest, Ekiti and Osun States each had two affected LGAs. In the South South zone, Cross River State had one affected LGA. In the North West, Kebbi and Sokoto States each recorded two affected LGAs, while in the North Central and North East zones, Niger and Taraba States, respectively, each had two affected LGAs (Fig 4).

Fig 4. *Historical failure rate of EMS across the country*

Forecast for achieving 2030 Elimination Targets

Fig 5. illustrates the projected progression of LGAs across assessment stages. Across all geopolitical zones, most LGAs are projected to reach TAS-1 and subsequently progress to TAS-3 by or before the 2030 elimination endline, indicating that both cessation of MAM and achievement of elimination thresholds are programmatically feasible within the target timeline. Only a small subset of LGAs in the Northern and south-south zone extends beyond 2030, which are attributable to later starting points rather than programmatic failure.

Fig 5. *Projected progression of EUs across assessment stages across ~~the country~~*

By 2030, we project that MAM would have stopped in at least 497 EUs i.e., 85.2% of all endemic IUs, and 309 (62.2%) of those would have passed TAS-3 (achieve elimination). However, under a conservative assumption that 3-5% of planned surveys can fail, these figures decline to 454 (77.9%) EUs stopping MAM and 284 achieving elimination (Fig. 6). Considering the historical failure rate of 3.4% at the EMS stage, we applied hypothetical failure scenarios of 3% and 5% to represent potential failure or delayed progression across evaluation units. As illustrated in Figure 6, even under these more conservative assumptions, the projected number of evaluation units achieving cessation of MAM (TAS-1) and elimination (TAS-3) remains largely stable across all geopolitical zones, with only modest reductions (difference of 1-4 LGAs) observed as failure rates increase, indicating that programme targets remain attainable despite plausible operational setbacks.

Fig 6: Forecast for achieving 2030 Targets (Stopping MAM and Passing TAS-3); *Baseline projections for 'Stop MDA' and 'Elimination' were derived from the national planned assessment schedule, assuming all planned surveys are completed successfully with no failure.*

Morbidity Management and Disability Prevention (MMDP) Program

The GPELF recommends that all individuals affected by the chronic manifestations of LF have access to a comprehensive package of care. In parallel with MAM, the NLFEP implements MMDP services for individuals already affected by LF-related morbidity, particularly lymphoedema and hydrocele [1,11]. MMDP activities include identifying and documenting cases, training health workers on essential care packages for lymphoedema management, establishing routine reporting systems, and ensuring access to basic supplies for hygiene and acute attack management [5, 10-12]. For hydrocele, the programme is expected to estimate surgical need, facilitate access to quality-assured hydrocelectomy services, and report on surgical outcomes [5, 10-12]. These interventions not only alleviate symptoms but also prevent disease progression and reduce the frequency of debilitating episodes. To ensure sustainability, the package is expected to be integrated into primary healthcare (PHC) services, alongside measures that address stigma, promote mental health, and foster social inclusion for those affected [5].

Health Facility Readiness Assessment for MMDP services

As part of best practices before implementing MMDP activities, the WHO recommends conducting health facility assessments to evaluate the readiness of facilities to provide services in a safe and high-quality setting [29]. The MMDP service in Nigeria commenced with a situational analysis to understand the burden of LF related morbidities and the readiness of health facilities to provide minimum or basic essential care to identified patients in the country. These assessments were conducted conducted using the Hydrocele Facility Assessment (HFAT) and Lymphoedema Facility Assessment (LFAT) tools [30]. The inspection protocol was deployed on the Comcare

application and administered by trained health workers. The tool has ten key components of care including background information of the facilities: surgical procedures, staff training, emergency patient transfer plans, infrastructure, essential medicines availability, disposable and non-disposable equipment, current hydrocele surgery practices, infection prevention and laboratory capacity to analyze samples during pre- and post-surgical investigations [30].

For HFAT, 154 (30.4%) of 508 facilities was assessed across 24 states, and 58 (37.7%) had a test score of 95% and above and were deemed qualified to conduct surgery (S2 Dataset 2, Fig 7). For LFAT, 716 facility was assessed across 212 LGAs, and 284 (39.7%) had a test score of 75% and above and were deemed ready to provide essential care for lymphoedema patients (S2 Dataset 2, Fig 8).

Fig 7: Map of Nigeria showing the distribution of health facilities assessed for readiness for providing surgical care for hydrocoele patients. *This map was created using primary data collected by the authors (S2 Dataset 2) and developed in ArcGIS v10.8 software. Shapefiles for Nigeria, including boundary polygons across all administrative levels, were sourced from publicly accessible databases available at Humanitarian Data Exchange, and are licensed under CC BY 4.0 (https://data.humdata.org/dataset/cod-ab-nga). No changes were made to the original shapefiles. The authors grant permission for the reuse of this map without restriction, provided appropriate credit is given to the source.*

Fig 8: Map of Nigeria showing the distribution of health facilities assessed for readiness for providing care for lymphoedema patients. *This map was created using primary data collected by the authors (S2 Dataset 2) and developed in ArcGIS v10.8 software. Shapefiles for Nigeria, including boundary polygons across all administrative levels, were sourced from publicly accessible databases available at Humanitarian Data Exchange, and are licensed under CC BY 4.0 (https://data.humdata.org/dataset/cod-ab-nga). No changes were made to the original shapefiles. The authors grant permission for the reuse of this map without restriction, provided appropriate credit is given to the source.*

Capacity building for MMDP Care

The NLFEP conducted training at different levels to empower the health workforce with the skill set needed to provide quality care and management to all identified cases that came into the health system. A WHO team of trainers and consultants conducted training-of-trainers sessions using the Filariacele Anatomical Surgical Task Trainer (FASST) module, supplemented by demonstrations on mannequins and live surgical procedures [31]. Thereafter, a structured cascading plan was rolled out in clusters and selected surgeons from all six geopolitical zones were trained on the FASST procedure to improve surgical outcomes. Additionally, training of trainers for lymphoedema management was also done to ensure harmonization in the provision of care by health workers from all the primary care centers.

Tracking Cases for Morbidity Management and Disability Prevention Services

Tracking of cases is useful for estimating the burden of morbidity, guiding surgical planning, monitoring access to care, and documenting progress toward MMDP targets. The hydrocele tracker, a standardized programmatic data collection and reporting tool, is used by the programme to systematically record, monitor, and report the identification, referral, surgical management, and follow-up of hydrocele cases [32]. The MMDP arm of the NLFEP leveraged MAM campaigns in endemic LGAs to facilitate the identification of suspected morbidity cases, specifically hydrocele and lymphoedema. CDDs in collaboration with frontline health workers, compiled waiting lists of suspected cases during MAM activities. These community-level data served as the foundation for morbidity case estimation, enabling the program to assess the disease burden across implementation areas. Training of trainers on the hydrocele tracker and cascading of the training

equipped programme staff with the requisite skills to track patients across the continuum of pre-operative, intra-operative, and post-surgical care.

Table 6 presents the number of lymphoedema, and hydrocele cases reported and managed across accredited health facilities in Nigeria between 2021 and 2025, disaggregated by geopolitical zones. The proportion LGAs reporting cases varied across zones, with the North Central zone reporting from 114 of 121 LGAs (94.3%), the Northeast from 65 of 112 LGAs (58.1%), the Northwest from 126 of 186 LGAs (67.8%), the Southeast from 74 of 95 LGAs (77.9%), the South South from 80 of 123 LGAs (65.1%), and the Southwest from 110 of 137 LGAs (80.3%).

For hydrocele, a total of 503 LGAs reported cases, totaling 22,195 cases, with 72.4% of these cases being managed across the country (Table 6). By zones, the Northeast and Northwest reported the highest number of cases, with 7,159 and 7,610 cases respectively, and 97.2% and 75% of these being managed. In contrast, the Southeast and Southwest zones reported fewer cases, 1,221 and 807 respectively, with lower proportions managed (26.4% and 25.1%). The North Central zone reported 2,523 hydrocele cases, of which 1,603 (63.6%) were managed, while the South South reported 2,875 cases with 1,261 (43.9%) managed (Table 5).

For lymphoedema, 450 LGAs across the country reported cases, accounting for 7,824 cases, of which 5,828 (74.5%) were actively managed. The North Central zone had the highest number of LGAs reporting lymphoedema cases (104, 86%) with 2,490 cases reported and 2,047 (82.3%) managed. The Northeast had 65 LGAs reporting 2,135 cases, of which 2,004 (93.9%) were managed, while the Northwest reported 78 LGAs with 1,383 cases, 1,002 (72.5%) of which were

managed. The Southeast and South South zones had relatively lower numbers of lymphoedema cases, with 712 and 367 cases reported respectively, and only 17.9% and 15.3% managed. The Southwest reported 737 cases from 96 LGAs, with 592 (80.4%) being managed (Table 6). Overall, the reporting trend for both hydrocele and lymphoedema was low at inception of the program, however sustained stakeholder's engagement and collaboration led to increased reporting in the subsequent years. The morbidity estimates generated from this process were instrumental in informing strategic planning, resource allocation, and prioritization of MMDP interventions at the state level (Fig 9).

Table 6: Number of hydrocoele and lymphoedema cases reported and managed across accredited health facilities between 2021 and 2025.

	**Zones**						
	North Central	North East	North West	South East	South South	South West	Total
**Indicators**							
No. of States in zone	7	6	7	5	6	6	37
No. of States reporting cases (%)	7 (100)	6 (100)	7 (100)	5 (100)	6 (100)	6 (100)	37 (100)
No. of LGAs in zone	121	112	186	95	123	137	774
No. of LGAs reporting cases (%)	114 (94.3)	65 (58.1)	126 (67.8)	74 (77.9)	80 (65.1)	110 (80.3)	569 (73.6)
**Lymphoedema**							
No. of LGAs reported (%)	104 (86.0)	65 (58.1)	78 (42.0)	55 (57.9)	52 (42.3)	96 (70.1)	450 (58.2)
No. of cases reported	2490	2135	1383	712	367	737	7824
No. of cases managed (%)	2047 (82.3)	2004 (93.9)	1002 (72.5)	127 (17.9)	56 (15.3)	592 (80.4)	5828 (74.5)
**Hydrocele**							
No. of LGAs reported (%)	98 (81.0)	76 (67.9)	110 (59.2)	73 (76.9)	70 (57.0)	76 (55.5)	503 (65.0)
No. of cases reported	2523	7159	7610	1221	2875	807	22195
No. of cases managed (%)	1603 (63.6)	6957 (97.2)	5705 (75.0)	322 (26.4)	1261 (43.9)	202 (25.1)	16050 (72.4)

__Fig 9:__ Line graph showing the trend of cases reported and managed for lymphoedema and hydrocoele in Nigeria between 2021 and 2025.

Post elimination surveillance strategies

Following WHO guidelines [11], continued PTS remains important until all IUs in Nigeria achieve interruption of transmission. The NLFEP has recommended continued surveillance in Nasarawa and Plateau States, especially in hard-to-reach areas and endemic border LGAs, where the risk of re-introduction may be higher. However, PTS activities have largely been limited to operational research studies involving both children and adults in selected LGAs, with broader implementation constrained by financial limitations [33].

Integrated vector control initiatives

Vector control interventions, such as the distribution of ITNs, are recommended for LF control but are not currently implemented as part of NFLEP activities. However, ITN distribution is routinely conducted through the national malaria elimination programme [19, 26]. As of 2024, 59.2% of households owned at least one ITN, with an average of 1.3 ITNs per household. However, only 31.5% of households had adequate ITN access, defined as at least one ITN for every two household members. Nationally, 42.5% of children under five years and 45.5% of pregnant women reported sleeping under an ITN the night preceding the survey (Table 7).

Marked geographical disparities were observed across the zones. The Northwest recorded the highest household ownership of at least one ITN (81.1%) and the highest average number of ITNs per household (2.0). Utilization patterns generally mirrored ownership levels, with the Northwest reporting the highest utilization among pregnant women (59.6%) and among the highest utilization

rates among children under five years (52.2%). In contrast, the Southwest recorded the lowest utilization rates among both children under five (22.6%) and pregnant women (20.1%) (Table 7).

At the state level, the highest household ownership of at least one ITN was observed in Zamfara (89.7%), Kano (87.1%), Jigawa (86.7%), Benue (82.3%), Kebbi (81.5%), and Cross River (80.2%). The highest utilization among children under five was recorded in Kano (67.5%), Zamfara (64.8%), and Yobe (62.0%). Plateau and Nasarawa States reported relatively low ownership levels (37.5% and 47.0%, respectively), with utilization rates ranging from 22.8% to 37.4% among children under five and from 24.9% to 25.2% among pregnant women (Table 7). To support continued promotion of long-lasting insecticidal nets (LLINs), the Carter Center has assisted in the distribution of 79,647 LLINs since 2017, specifically targeting cross-border communities in Plateau and Nasarawa States [33].

Table 7: Ownership and utilization rate of treated bed-nets across the country as of 2024.

Zone	State	% of HH with at least one ITNs	Average number of ITNs per HH	% of HH with at least one ITN for every two persons	% of children under age 5 who slept under an ITNs	% of pregnant women who slept under an ITNs
**South East**	Abia	25.4	0.4	13.3	20.0	1.1
	Anambra	56.8	1.3	35.2	37.0	38.3
	Ebonyi	74.4	1.7	32.9	31.6	36.5
	Enugu	17.0	0.2	6.5	13.3	7.1
	Imo	32.8	0.6	17.5	11.3	8.2
	Total	42.6	0.9	22.0	24.2	23.4
**Southwest**	Ekiti	25.4	0.4	14.8	16.1	19.3
	Lagos	31.9	0.5	18.1	19.2	18.8
	Ogun	33.7	0.5	18.3	10.7	1.9

	Ondo	58.4	1.1	41.8	31.5	40.1
	Osun	59.0	0.9	37.4	39.8	25.1
	Oyo	39.1	0.6	19.4	27.5	15.5
	Total	40.1	0.6	23.6	22.6	20.1
**South South**	Akwa Ibom	70.9	1.9	59.9	25.4	29.2
	Bayelsa	38.9	0.6	15.0	34.6	20.6
	Cross River	80.2	1.8	64.6	51.4	30.4
	Delta	58.0	1.3	40.5	39.5	31.0
	Edo	21.0	0.4	9.0	15.4	11.2
	Rivers	29.1	0.5	14.2	23.6	18.1
	Total	48.6	1.1	33.6	30.6	23.6
**Northeast**	Adamawa	78.6	2.0	48.9	47.9	42.6
	Bauchi	76.4	2.1	44.8	50.5	53.5
	Borno	57.3	0.9	20.6	39.6	36.1
	Gombe	79.2	1.9	33.7	52.2	59.1
	Taraba	70.9	1.7	40.6	42.6	56.2
	Yobe	73.4	1.9	34.1	62.0	54.5
	Total	71.9	1.7	37.4	49.3	49.8
**Northwest**	Jigawa	86.7	2.2	38.9	55.2	73.7
	Kaduna	72.0	1.6	33.0	43.7	50.9
	Kano	87.1	2.6	46.5	67.5	82.4
	Katsina	75.8	1.7	28.3	45.7	51.6
	Kebbi	81.5	1.8	30.9	52.6	60.1
	Sokoto	78.5	1.9	32.3	19.3	18.8
	Zamfara	89.7	2.2	40.3	64.8	74.6
	Total	81.1	2.0	36.7	52.2	59.6
**North Central**	Benue	82.3	2.0	60.2	58.5	58.7
	FCT, Abuja	40.3	0.6	19.4	36.6	27.9
	Kogi	38.8	0.7	20.1	26.8	30.1
	Kwara	36.9	0.6	16.4	12.5	16.7
	Nasarawa	47.0	0.8	20.4	37.4	24.9
	Niger	72.0	1.8	42.8	48.4	55.1
	Plateau	37.5	0.8	19.9	22.8	25.2
	Total	54.2	1.2	31.2	37.6	38.5
**Total**		59.2	1.3	31.5	42.5	45.5

Table was extracted Nigeria DHS 2023–24 **report [19] and based on** *children and pregnant women who stayed in the household the night before the interview; LGAs: Local Government Areas; HH: Household; ITNs: Insecticide-treated net (ITN) also known as long-lasting insecticidal net (LLIN).*

Discussion

The NLFEP has achieved substantial progress in reducing the burden of LF and advancing toward the 2030 global elimination target [11]. Prior to this study, elimination had only been documented in Plateau and Nasarawa States [33], while significant progress had been reported in the Federal Capital Territory [34]. This study therefore provides a more comprehensive assessment of Nigeria's LF elimination programme since its inception in 2000. Our findings shows that LF mapping has been completed nationwide between 2000 and 2011, and all endemic 583 LGAs had initiated MAM, with more than 90% of them completing the minimum effective MAM rounds required for impact assessment. By 2025, approximately 60% of endemic LGAs had stopped MAM across 30 of Nigeria's 37 states. Furthermore, a growing number of LGAs had progressed along the elimination pathway, with approximately 7% passing TAS-2, and about 5% passing TAS-3. Based on these trends, we project that by 2030, MAM will have been stopped in 497 (85.2%) of all endemic LGAs), and 309 LGAs will have achieved elimination.

In parallel with efforts to interrupt transmission, the NLFEP has strengthened health systems for MMDP through improvements in health-facility readiness, capacity building of healthcare workers, and integration of MMDP activities into routine immunization and polio services, seasonal malaria chemoprevention campaigns, MAM campaigns, and disease surveillance and notification systems. For instance, trachoma trichiasis case-finding activities were also leveraged

to identify individuals with LF-related morbidity [35]. This integrated approach expanded opportunities for case detection, optimized the use of existing health-system resources, minimized duplication of efforts, and improved service delivery efficiency. Beyond strengthening service availability, emerging evidence indicates that these interventions have generated substantial benefits for affected individuals, with improvements in mobility, reduced pain, enhanced psychological well-being, increased social participation, and greater economic productivity [36,37].

Despite the considerable progress recorded in both transmission interruption and morbidity management, important operational and programmatic challenges remain that could impede attainment of the 2030 elimination targets. Foremost among these is insecurity, which remains a major obstacle to the implementation of MAM, community sensitization activities, and epidemiological assessments [38-41]. The consequences of these disruptions are most apparent in northern Nigeria, particularly in Borno State, where prolonged insecurity delayed the initiation of MAM until 2019 and limited most endemic LGAs to only 1–4 effective treatment rounds [32]. Similar disruptions arising from communal conflicts, insurgency, kidnappings, and other security threats have been reported in several endemic areas across the country. To manage these challenges, the NLFEP has adopted context-specific delivery approaches including leveraging local intelligence to assess security risks, implementing proxy training models for CDDs, conducting "hit-and-run" treatment campaigns under security escort, and providing services through safer neighboring communities accessible to affected populations [42]. While these adaptations have enabled continued programme activities in some settings, its implementation

across Nigeria remains largely ad hoc, with varying levels of risk tolerance and operational flexibility among donors and implementing partners. Consequently, there is a need for a nationally coordinated framework that strengthens collaboration among NTD stakeholders while guiding security risk assessment and adaptive implementation approaches to ensure the safe and effective delivery of NTD interventions in insecurity-affected settings.

Furthermore, funding constraints and limited operational resources continue to impede progress toward LF elimination by delaying medicine distribution, coverage evaluation surveys, and epidemiological assessments [41-43]. These constraints were more pronounced in southern states and have the potential to undermine the quality and effectiveness of MAM implementation. Delays in conducting coverage evaluation surveys are particularly problematic because these assessments are critical for identifying systematically missed populations, treatment non-compliance, and operational bottlenecks that limit programme reach [41-43]. Without timely assessments, opportunities to refine implementation strategies and improve treatment coverage may be lost [44,45]. In addition, delays in epidemiological assessments can postpone critical programmatic decisions regarding areas eligible to stop MAM or progress along the elimination pathway. Such delays are not only operationally inefficient but may also result in the continued implementation of MAM in areas where transmission has already been interrupted but cannot be formally verified due to insufficient resources for assessment [33]. Sustained domestic financing, continued partner engagement, and strategic resource mobilization will therefore be essential moving forward.

One potential strategy for sustaining programme implementation in the face of limited resources is the mainstreaming of MAM and other NTD interventions within broader Universal Health Coverage (UHC) and primary healthcare frameworks [46]. There is growing recognition that integrating NTD services into routine health systems can strengthen domestic resource mobilization, reduce reliance on external funding, and enhance the long-term sustainability of programme operations [47, 48]. In parallel, collaborative mechanisms among implementing partners—including joint planning, resource sharing, and coordinated allocation of available funding—have emerged as important approaches for mitigating resource constraints and maintaining programme continuity. Such collaborative approaches have been piloted in several Nigerian states for diseases including onchocerciasis, schistosomiasis, and soil-transmitted helminthiasis [48], as well as for broader public health priorities such as maternal and child health [47]. These partnerships have facilitated the timely implementation of coverage surveys and operational assessments [26,44,49,50], improved identification of missed populations, enabled refinement of delivery strategies [44,45], strengthened community engagement, and enhanced treatment compliance. Expanding these collaborative and integrated approaches will be critical for addressing operational gaps, improving programme efficiency, and accelerating progress toward the 2030 LF elimination targets.

Beyond the challenges, the WHO recommends the use of complementary vector-control interventions as part of integrated approaches to accelerate and sustain the elimination of lymphatic filariasis in endemic settings [11]. Although vector-control activities are not currently a core component of NLFEP implementation [51], there is growing evidence that insecticide-treated nets (ITNs) can contribute to reducing LF transmission, particularly in areas where *Anopheles*

mosquitoes serve as the principal vectors [52,53]. Findings from the 2025 NDHS report indicate that ITN ownership, access, and utilization remain suboptimal in many endemic areas of Nigeria [19]. Strengthening access to and utilization of vector-control interventions may therefore provide additional benefits for both LF and malaria control programmes. As Nigeria moves closer to achieving LF elimination targets, greater consideration should be given to integrating complementary vector-management strategies within broader elimination and post-elimination frameworks to help sustain gains and reduce the risk of recrudescence.

Finally, as an increasing number of implementation units progress toward TAS-3 and elimination as a public health problem, it is imperative that standardized post-validation surveillance systems are established within the NLFEP [33]. A clearly defined surveillance strategy should include targeted investigations of antigen-positive individuals and clusters, continued monitoring through sentinel and spot-check sites, integration of LF testing into large-scale population-based surveys, and the use of xenomonitoring where feasible [33,54]. Attention should be given to border communities, migrant and mobile populations, and other settings at increased risk of reintroduction or resurgence [38,39,40,55]. Strengthening post-elimination surveillance will be essential to safeguarding elimination gains and ensuring the long-term interruption of transmission.

Conclusion

Nigeria has made substantial progress toward the elimination of lymphatic filariasis, completing nationwide mapping, implementing MAM across all endemic implementation units, and advancing a growing number of areas through TAS. The programme has also strengthened MMDP services through investments in health-system capacity, service integration, and expanded access

to care for individuals affected by LF-related morbidity. Collectively, these achievements position Nigeria favorably towards the WHO 2030 elimination targets. However, important challenges remain, particularly insecurity, funding constraints, and the need for strengthened post-elimination surveillance systems. Sustaining progress will require continued political commitment, increased domestic ownership, strategic partner collaboration, and targeted investments to support timely assessments, adaptive programme implementation, and robust surveillance in areas that have interrupted transmission. Addressing these challenges while consolidating recent gains will be critical to achieving, validating, and sustaining the elimination of lymphatic filariasis as a public health problem in Nigeria.

Acknowledgements

We extend our sincere appreciation to all program staff at the national, state, and implementation-unit levels for their sustained commitment and contributions to the implementation of these programs over the years. We also acknowledge the contributions of academic partners across multiple institutions whose technical input supported various stages of the control program. We further appreciate the continued support of international and national non-governmental development organizations whose contributions facilitated program implementation and evaluation, including Amen Health Care and Empowerment Foundation (AMEN), Christoffel Blinden Mission International (CBM), Health and Development Support Programme (HANDS), Mission to Save the Helpless (MITOSATH), Sightsavers, The Carter Center (TCC), Helen Keller International (HKI), RTI/ENVISION, and UNICEF.

Financial Support

The activities and interventions reported in this study were supported through funding and programmatic support from several international and national non-governmental development organizations (NGDOs), including AMEN, CBM, HANDS, MITOSATH, Sightsavers, The Carter Center, HKI, RTI/ENVISION, and UNICEF. The funding organizations had no role, as institutions, in the conceptualization, study design, data analysis, decision to publish, or preparation of the manuscript. Individual contributors affiliated with these organizations who met authorship criteria participated in the study in their personal and scientific capacity as co-authors.

Authors' contributions

OBA, HOM, BA, DP, JST, OH, OF, FO (Oyediran) conceptualized the study, OBA, HOM, BA, DP, JST, OF, FO (Oyediran) prepared the protocol, RO, OO, SO, AW, IE, IR, FO (Olamiju), IA, MA, EE, JS, AG, JST, EM, AS, CI, UE, CO, EU, OF, UE, KBG, HA participated in supervised field surveys and data collection; HOM performed all statistical analyses: HOM prepared the first draft of the manuscript; OBA, BA, DP, JST, OH, FO (Oyediran), SI, FO (Olamiju) revised the manuscript. All authors contributed to the development of the final manuscript and approved its submission.

Availability of data and materials

The datasets analyzed during this study are also provided as supplementary materials accompanying this article.

Conflicts of interest.

The authors declare that they have no conflicts of interest.

References

1. World Health Organization Lymphatic filariasis. Available at https://www.who.int/news-room/fact-sheets/detail/lymphatic-filariasis. [accessed 21 June 2026]

2. World Health Organization Ending the neglect to attain the sustainable devel- opment goals: A road map for neglected tropical diseases 2021–2030 . Geneva, Switzerland: World Health Organization; 2020 [accessed 21 June 2026]

3. Shenoy RK. Clinical and Pathological Aspects of Filarial Lymphedema. Korean J Parasitol. 2008;46(3):119–25.

4. Resolution of the World Health Assembly. WHA50.29. Agenda item 20 Elimination of lymphatic filariasis as a public health problem ; 1997. World Health Assembly, Geneva, Switzerland Available at https://apps.who.int/ iris/ bitstream/ handle/ 10665/ 179773/ WHA50_R29_eng.pdf. [accessed 21 June 2026]

5. Ottesen EA. Editorial: The global programme to eliminate lymphatic filariasis. Tropical Med Int Health 2000; 5 :591–4. doi: 10.1046/j.1365-3156.20 0 0. 00620.x

6. Ichimori K, King JD, Engels D, Yajima A, Mikhailov A, Lammie P, et al. Global programme to eliminate lymphatic filariasis: the processes underlying programme success. PLoS Negl Trop Dis 2014; 8 :e3328. doi: 10.1371/journal.pntd. 0 0 03328

7. ENVISION. Calculating coverage for NTDs: Types of coverage. Available at https://www.ntdtoolbox.org/sites/default/files/content/paragraphs/resource/files/2019-08/calculating_coverage_job_aid_final.pdf. [accessed 21 June 2026]

8. Michael E, Malecela-Lazaro MN, Kabali C, Snow LC, Kazura JW. Mathematical models and lymphatic filariasis control: endpoints and opti- mal interventions. Trends Parasitol 2006; 22 :226–33. doi: 10.1016/j.pt.2006.03. 005 .

9. Agrawal VK, Sashindran VK. Lymphatic filariasis in India: problems, challenges and new initiatives. Med J Armed Forces India 2006; 62 :359–62. doi: 10.1016/ S0377-1237(06)80109-7 .

10. Mackenzie CD, Lazarus WM, Mwakitalu ME, Mwingira U, Malecela MN. Lymphatic filariasis: patients and the global elimination programme. Ann Trop Med Parasitol 2009; 103 :S41–51. doi: 10.1179/000349809x12502035776630 .

11. World Health Organization (WHO). Validation of elimination of lymphatic filariasis as a public health problem. Available at https://www.who.int/publications/i/item/9789241511957. **ISBN:** 978 92 4 151195 7; WHO/HTM/NTD/PCT/2017.1. World Health Organization - Licence: CC BY-NC-SA 3.0 IGO. [accessed 21 June 2026]

12. World Health Organization (WHO). Managing morbidity and preventing disability in the Global Programme to Eliminate Lymphatic Filariasis -- WHO position statement. Available at https://www.who.int/publications/i/item/WHO-HTM-NTD-2011.8. WHO/HTM/NTD/PCT2011.8. World Health Organization - Licence: CC BY-NC-SA 3.0 IGO. [accessed 21 June 2026]

13. World Health Organization Weekly Epidemiological record. Global programme to eliminate lymphatic filariasis: progress report. Geneva: World Health Organization; 2024. https://iris.who.int/server/api/core/bitstreams/c208d073-e744-4a03-8af2-dbe537a5fbb1/content [accessed 21 June 2026]

14. Cano J, Rebollo MP, Golding N, Pullan RL, Crellen T, Soler A, Kelly-Hope LA, Lindsay SW, Hay SI, Bockarie MJ, Brooker SJ. The global distribution and transmission limits of lymphatic filariasis: past and present. Parasit Vectors. 2014 Oct 11;7:466. doi: 10.1186/s13071-014-0466-x.

15. Hotez PJ, Asojo OA, Adesina AM. Nigeria: "Ground Zero" for the high prevalence neglected tropical diseases. PLoS Negl Trop Dis. 2012;6(7):e1600. doi: 10.1371/journal.pntd.0001600. Epub 2012 Jul 31.

16. World Health Organization Regional Office for Africa Expanded Special Project for the Elimination of Neglected Tropical Diseases (ESPEN) portal. Geneva: World Health Organization Regional Office for Africa; 2024

17. Waje T, Iliyasu C, Yaki LM, Auta IK. A review of epidemiology of lymphatic filariasis in Nigeria. Pan Afr Med J. 2024 Mar 27;47:142. doi: 10.11604/pamj.2024.47.142.39746.

18. Adamu H, Hussaini A, Isaac C, Rahimat H, Collins I, Cedric O, et al. The Burden of Bancroftian Filariasis in Nigeria : A Review. Ethiop J Health Sci. 2020;30(2):301

19. Federal Ministry of Health and Social Welfare of Nigeria (FMoHSW), National Population Commission (NPC) [Nigeria], and ICF. 2024. Nigeria Demographic and Health Survey 2023–24: Key Indicators Report. Abuja, Nigeria, and Rockville, Maryland, USA: NPC and ICF. Available at https://dhsprogram.com/pubs/pdf/PR157/PR157.pdf. [accessed 21 June 2026]

20. World Dra Information. Nigeria. Available at https://www.worlddata.info/africa/nigeria/index.php. [accessed 21 June 2026]

21. Nigeria Master Plan for NTDs (2023-2027). Available at https://espen.afro.who.int/sites/default/files/content/document/Nigeria%20NTD%20Master%20Plan%202023%20-%202027.pdf. [accessed 21 June 2026]

22. World Health Organization Operational guidelines for rapid mapping of bancroftian filariasis in Africa . Geneva: World Health Organization; 2000.

23. World Health Organization Report of the WHO strategic and technical advisory group for neglected tropical diseases. Geneva: World Health Organization; 2015.

24. World Health Organization Preventive chemotherapy in human helminthiasis. Geneva: World Health Organization; 2006

25. Gass KM, Sime H, Mwingira UJ, Nshala A, Chikawe M, Pelletreau S, et al. The rationale and cost-effectiveness of a confirmatory mapping tool for lym- phatic filariasis: examples from Ethiopia and Tanzania. PLoS Negl Trop Dis 2017; 11 :e0 0 05944. doi: 10.1371/journal.pntd.0 0 05944

26. Akogun OB, Adewale B, Mogaji HO, Dawodu R, Bankole M, Adewlae A, Abdus-Salam IA, Ononogbo I, Olamiju FO, Tehemen J, Isiyaku S. Confirmatory mapping for lymphatic filariasis a decade after baseline endemicity assessment in highly urbanized settings of Lagos, Nigeria. Int J Infect Dis. 2025 Oct;159:108014. doi: 10.1016/j.ijid.2025.108014.

27. World Health Organization. Training in monitoring and epidemiological assessment of mass drug administration for eliminating lymphatic filariasis. Facilitators' guide. WHO/HTM/NTD/PCT/2013.8 https://www.who.int/publications/i/item/9789241505444. [accessed 21 June 2026]

28. World Health Organization. WHO Manual on Integrated Transmission Assessment Surveys (iTAS) for Onchocerciasis and Lymphatic Filariasis. Available at https://espen.afro.who.int/tools-resources/documents/who-manual-integrated-transmission-assessment-surveys-itas-onchocerciasis. [accessed 21 June 2026]

29. World Health Organization. Morbidity management and disability prevention (MMDP). Available at https://www.who.int/teams/control-of-neglected-tropical-diseases/interventions/strategies/Innovative-and-intensified-disease-management/morbidity-management-and-disability-prevention. [accessed 21 June 2026]

30. Sightsavers. Hydrocele facility assessment tool (HFAT) and lymphoedema assessment tool (LFAT).Available at https://ntd-sightsavers.hub.arcgis.com/content/4500c6a0143449faa8540f032dfff7e3/about**.** [accessed 21 June 2026]

31. Helen Keller International. Filaricele Surgery Training Package. Available at https://helenkellerintl.org/what-we-do/knowledge-resources/neglected-tropical-diseases/mmdp-project-resources/filaricele-surgery-training-package/. [accessed 21 June 2026]

32. Sightsavers. Hydrocele Tracker. Available at https://ntd-sightsavers.hub.arcgis.com/documents/152b5dc6dbe74b61a3e1cdbde5e01dfc/about. [accessed 21 June 2026]

33. Eigege A, Noland GS, Adelamo SE, Nwodu K, Sallau A, Umaru J, Mancha BS, Davies E, Danboyi J, Kadimbo JA, Saka YA, Anagbogu I, Miri ES, Richards FO. Post-Treatment Surveillance for Lymphatic Filariasis in Plateau and Nasarawa States, Nigeria: Results of Transmission Assessment Surveys. Am J Trop Med Hyg. 2020 Jun;102(6):1404-1410. doi: 10.4269/ajtmh.20-0020

34. Amanyi-Enegela JA, Kumbur J, Okunade F, Ashikeni D, Ishaya R, Sankar G, et al. Evaluating the effectiveness of mass drug administration on lymphatic filariasis transmission and assessment of post - mass drug administration surveillance in Nigeria's Federal Capital Territory. 2025;1–10.

35. Coalition for Operational Research in Neglected Tropical Diseases (COR-NTD). COVID-19 and NTD innovation. Available at https://www.cor-ntd.org/news-stories/blog/covid-19-and-ntd-innovation. [accessed 21 June 2026]

36. Eze CC, Ekeke N, Alphonsus C, et al. Effectiveness of self-care interventions for integrated morbidity management of skin neglected tropical diseases in Anambra State, Nigeria. BMC Public Health. 2021;21(1):1748. doi:10.1186/s12889-021-11729-1

37. Olamiju, F.O., Mogaji, H.O., Olamiju, O.J., Achu, I., Mbuko, E., Ovia, S., Tanko, E., Mahmood, D., Nasiru, H.B., Mohammed, R. and Abubakar, H., 2023. Impact of Hydrocoele Surgeries on Quality of Life for Persons Living in Lymphatic Filariasis Endemic Communities: https://doi. org/10.60787/f8gd-xx05. *Nigerian Journal of Parasitology*, (1). https://doi.org/10.60787/f8gd-xx05

38. Sangare M, Coulibaly YI, Ravichandran P, et al. Exploring the impact of mobile and migrant populations on mass drug administration coverage and effectiveness in Africa: A scoping review protocol. *PLoS One*. 2025;20(5):e0324949. doi:10.1371/journal.pone.0324949

39. Sangare M, Diabate AF, Coulibaly YI, et al. Understanding the barriers and facilitators related to never treatment during mass drug administration among mobile and migrant populations in Mali: a qualitative exploratory study. *BMJ Glob Health*. 2024;9(10):e015671. Published 2024 Oct 9. doi:10.1136/bmjgh-2024-015671

40. Sangare M, Coulibaly YI, Diabate AF, et al. Factors associated with low coverage in mass drug administration for schistosomiasis in mobile populations in mali: a cross-sectional study. *BMC Infect Dis*. 2025;25(1):1240. Published 2025 Oct 7. doi:10.1186/s12879-025-11626-7

41. Hietanen H, Pfavayi LT, Mutapi F. Unlocking the blueprint to eliminating neglected tropical diseases: A review of efforts in 50 countries that have eliminated at least 1 NTD. PLoS Negl Trop Dis. 2025 Sep 4;19(9):e0013424. doi: 10.1371/journal.pntd.0013424.

42. Harding-Esch EM, Bird F, Bashir A, Kelly-Hope LA. Challenges, barriers and opportunities for neglected tropical disease programs of conflict-affected African countries. Int Health. 2026;18(3):340-351. doi:10.1093/inthealth/ihaf149

43. Banda GT, Deribe K, Davey G. How can we better integrate the prevention, treatment, control and elimination of neglected tropical diseases with other health interventions? A systematic review. BMJ Glob Health. 2021 Oct;6(10):e006968. doi: 10.1136/bmjgh-2021-006968

44. Ekpo UF, Olamiju FO, Mogaji HO, Ovia SN, Oladipupo OO, Kehinde AY, Oyediran FO, Aderogba M, Makau-Barasa LK. Sensitivity of Three Impact Assessment Methodologies in Adjusting Preventive Chemotherapy Treatment Decisions for Schistosomiasis Elimination in Ondo State, Nigeria. Am J Trop Med Hyg. 2025 Feb 18;112(5):987-999. doi: 10.4269/ajtmh.24-0352. PMID: 39965211; PMCID: PMC12062661.

45. Olamiju F, Nebe OJ, Mogaji H, et al. Schistosomiasis outbreak during COVID-19 pandemic in Takum, Northeast Nigeria: Analysis of infection status and associated risk factors. *PLoS One*. 2022;17(1):e0262524. Published 2022 Jan 21. doi:10.1371/journal.pone.0262524

46. Rozendaal N, Bassabi-Alladji MA, Bishaw T, Omondi W, Tolotra AF, Batchiri SA, Nshimiyimana L, Gnossike P, Jones C, Mubangizi A, Rebollo M. Strengthening health systems through NTD integration: Key insights from a multi-country webinar. PLoS Negl Trop Dis. 2026 Feb 17;20(2):e0013628. doi: 10.1371/journal.pntd.0013628.

47. Guardian news. FG to integrate Azithromycin intervention into national child survival policy. Available at https://guardian.ng/features/health/fg-to-integrate-azithromycin-intervention-into-national-child-survival-policy/. [accessed 21 June 2026]

48. SCIDaR. Saving Lives: The Revolutionary SARMAAN Initiative in Nigeria. Available at https://scidar.org/saving-lives-the-revolutionary-sarmaan-initiative-in-nigeria/. [accessed 21 June 2026]

49. Mogaji HO, Olamiju FO, Oyinlola F, et al. Prevalence, intensity and risk factors of soil-transmitted helminthiasis after five effective rounds of preventive chemotherapy across three implementation units in Ondo State, Nigeria. *PLoS Negl Trop Dis*. 2025;19(1):e0012533. doi:10.1371/journal.pntd.0012533

50. Ekpo UF, Solomon J, Mogaji HO, et al. Updating the Endemicity Map of Soil-Transmitted Helminthiasis in Ten Local Government Areas of Ondo State, Southwestern Nigeria. *Trop Med Infect Dis*. 2026;11(1):24. doi:10.3390/tropicalmed11010024

51. Davis EL, Prada J, Reimer LJ, Hollingsworth TD. Modelling the Impact of Vector Control on Lymphatic Filariasis Programs: Current Approaches and Limitations. Clin Infect Dis. 2021 Jun 14;72(Suppl 3):S152-S157. doi: 10.1093/cid/ciab191.

52. Ashton RA, Kyabayinze DJ, Opio T, et al. The impact of mass drug administration and long-lasting insecticidal net distribution on Wuchereria bancrofti infection in humans and mosquitoes: an observational study in northern Uganda. Parasit Vectors. 2011;4:134. doi:10.1186/1756-3305-4-134

53. Rebollo MP, Sambou SM, Thomas B, et al. Elimination of lymphatic filariasis in the Gambia. PLoS Negl Trop Dis. 2015;9(3):e0003642. Published 2015 Mar 18. doi:10.1371/journal.pntd.0003642

54. Opara, K., Ikpeida, N., Udoidung, N., Adeleke, M., Chikezie, F., Yaro, C., Ekpo, N., Iwalewa, Z., Udoewah, S., & Silas, I. (2026). Molecular Xenomonitoring of Lymphatic Filariasis Transmission in Okobo LGA, Akwa Ibom State, Nigeria. International Journal of Life Sciences and Biotechnology, 9(1), 48-57. https://doi.org/10.38001/ijlsb.1845089

55. Adams MW, Sutherland EG, Eckert EL, Saalim K, Reithinger R. Leaving no one behind: targeting mobile and migrant populations with health interventions for disease elimination-a descriptive systematic review. BMC Med. 2022 May 9;20(1):172. doi: 10.1186/s12916-022-02365-6.

Supplementary File

S1: Dataset

S2: Dataset 2

Click here to access/download;Figure;Fig 1.tif

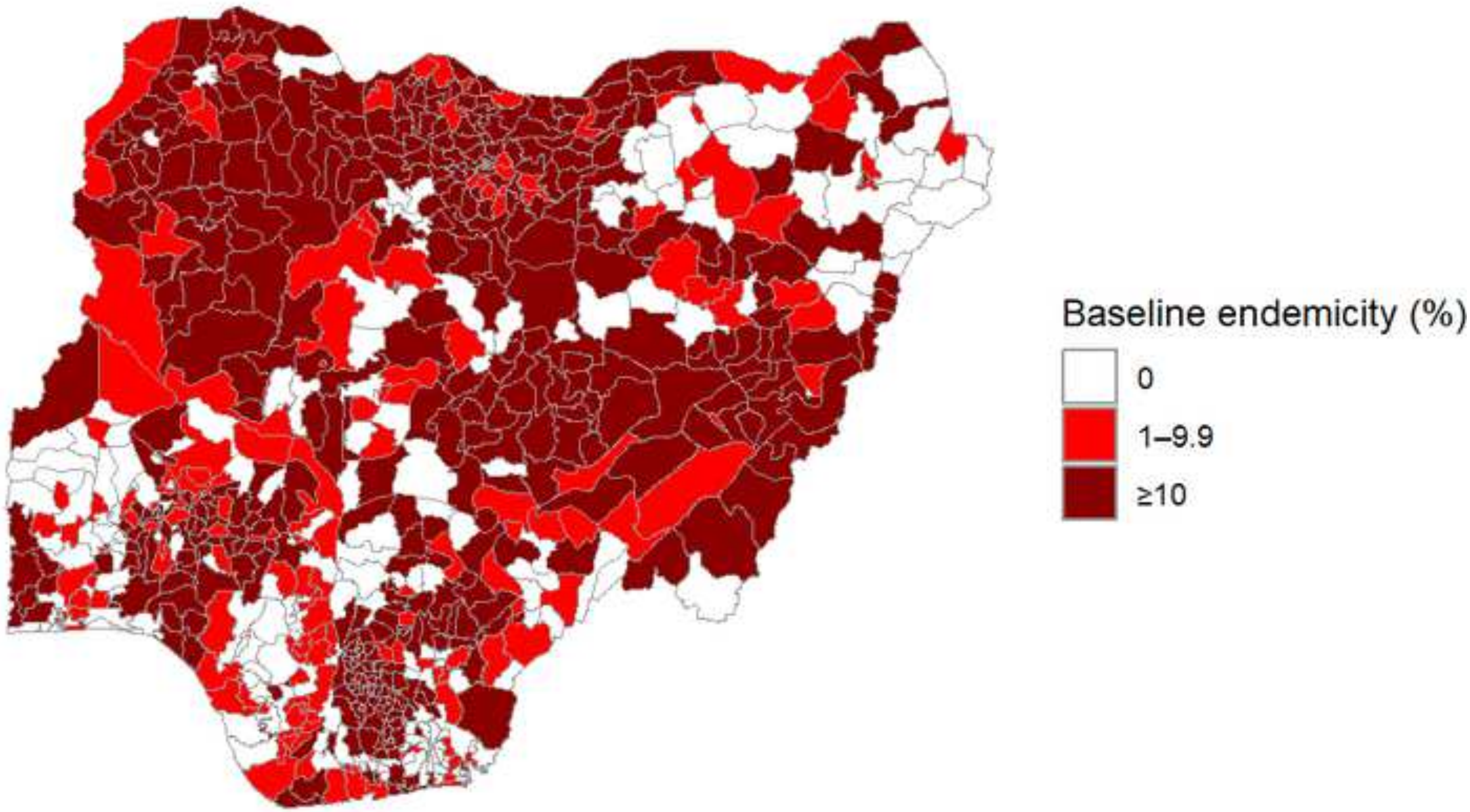

Click here to access/download;Figure;Fig 2.tif

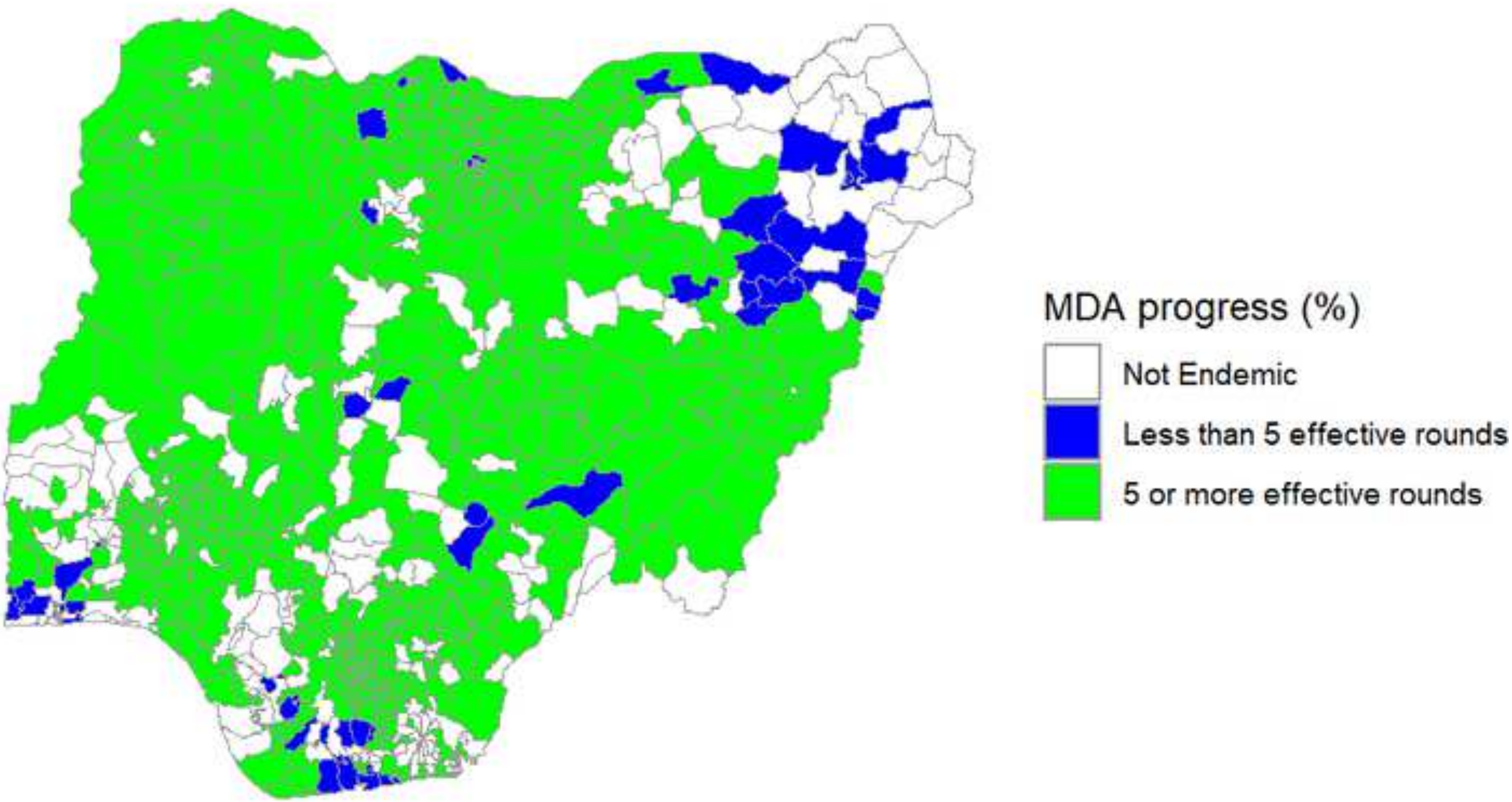

Click here to access/download;Figure;Fig 3.tif

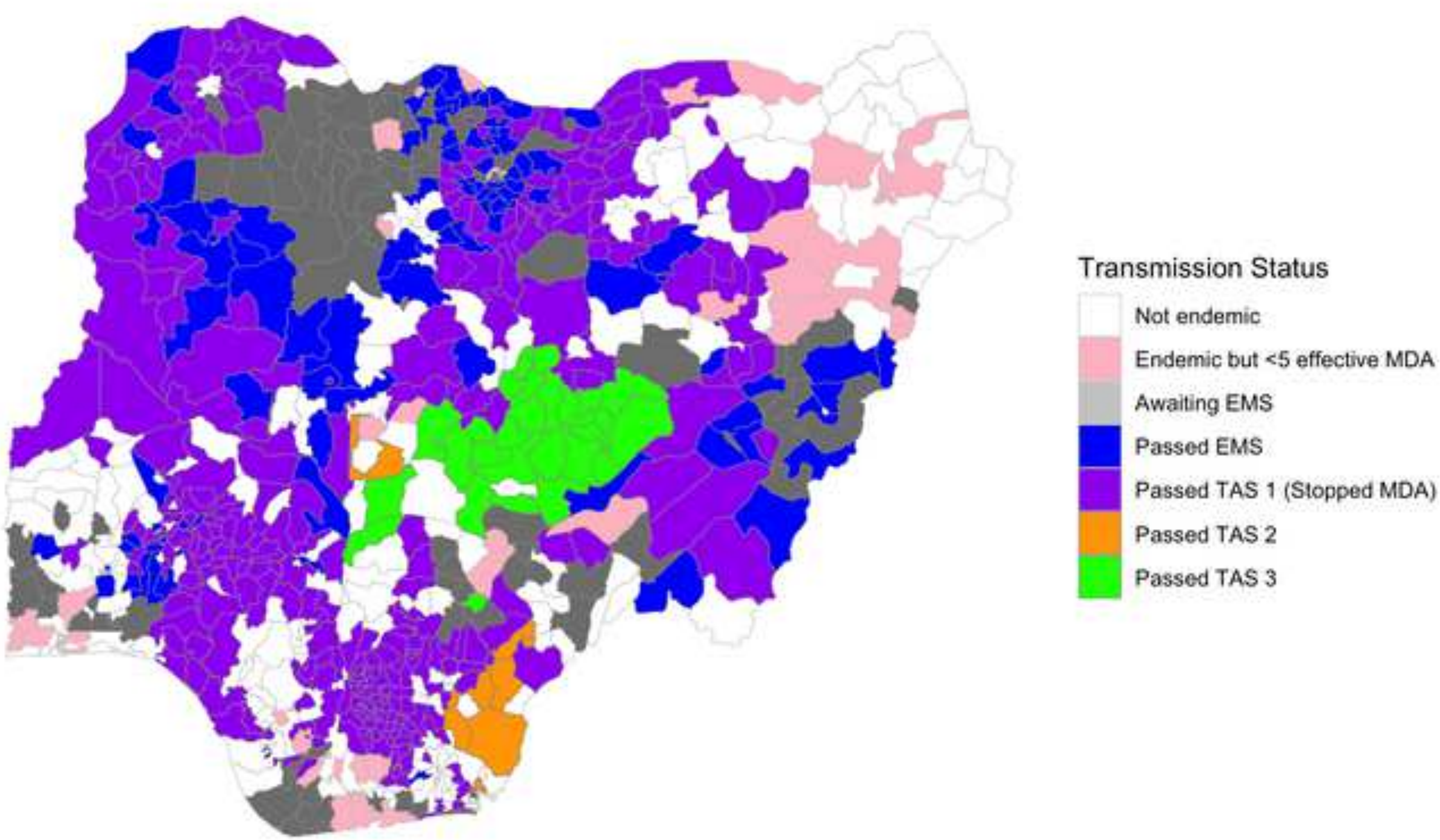

Click here to access/download;Figure;fig 4.tif

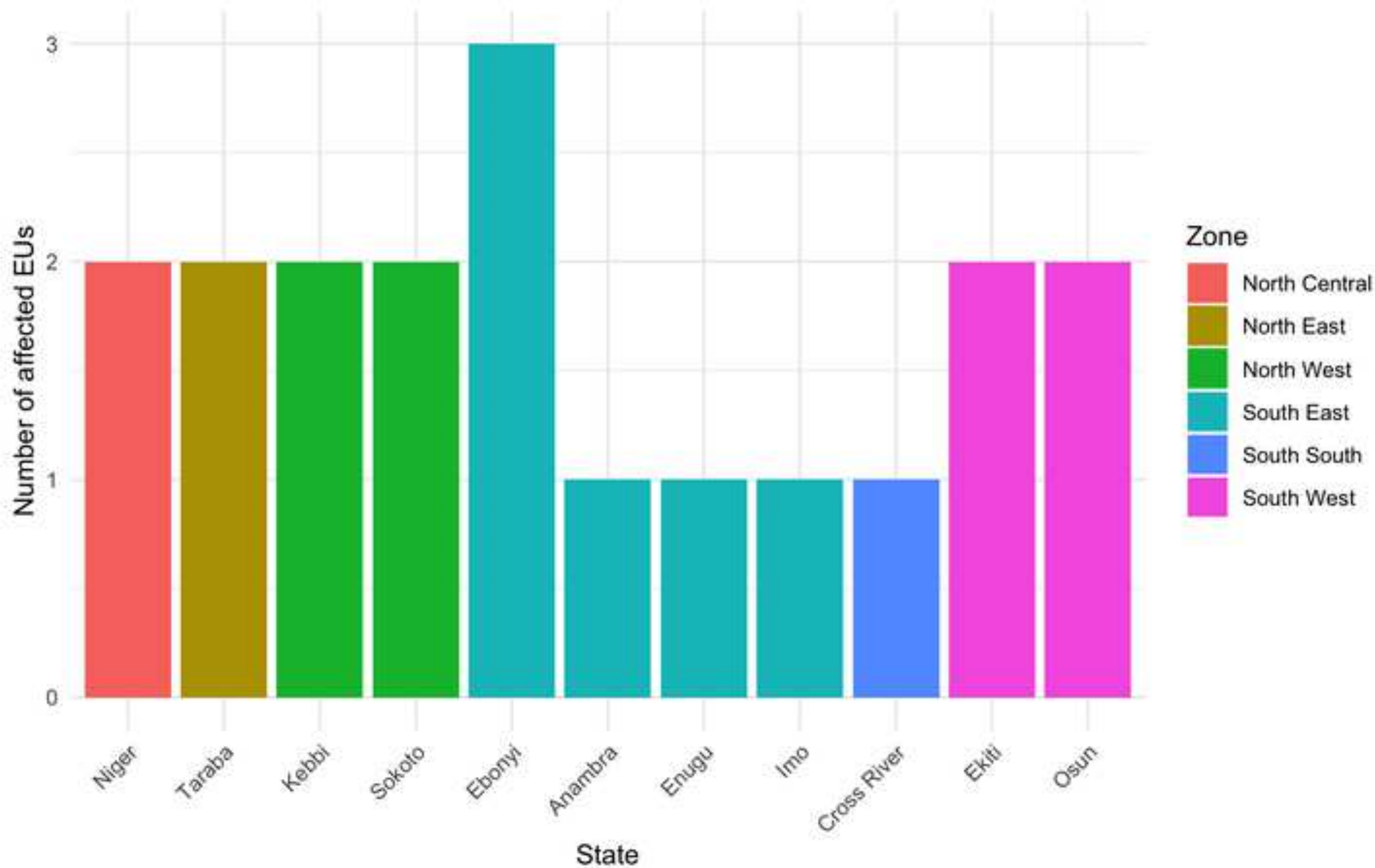

Click here to access/download;Figure;Fig 5.tif

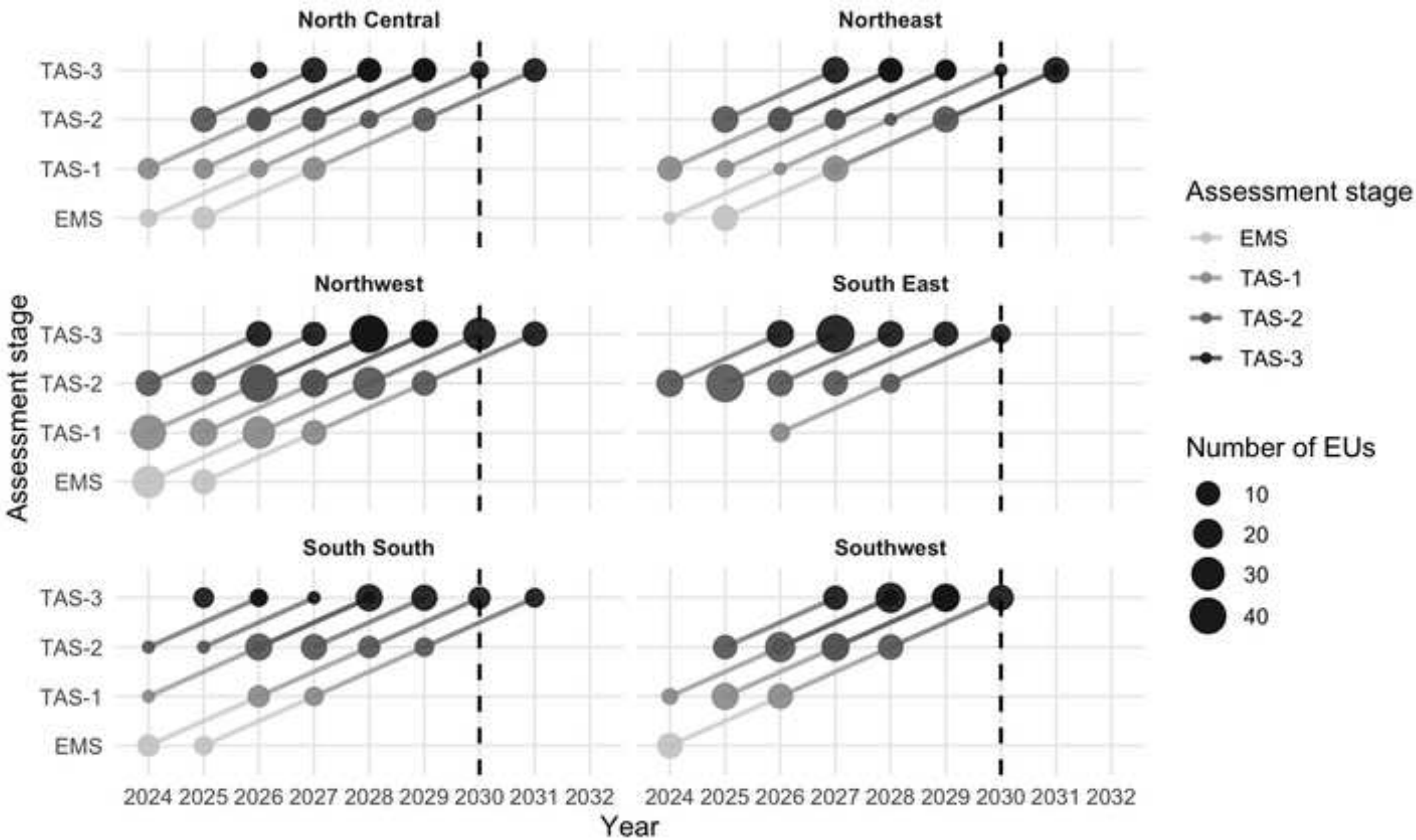

Click here to access/download;Figure;Fig 6.tif

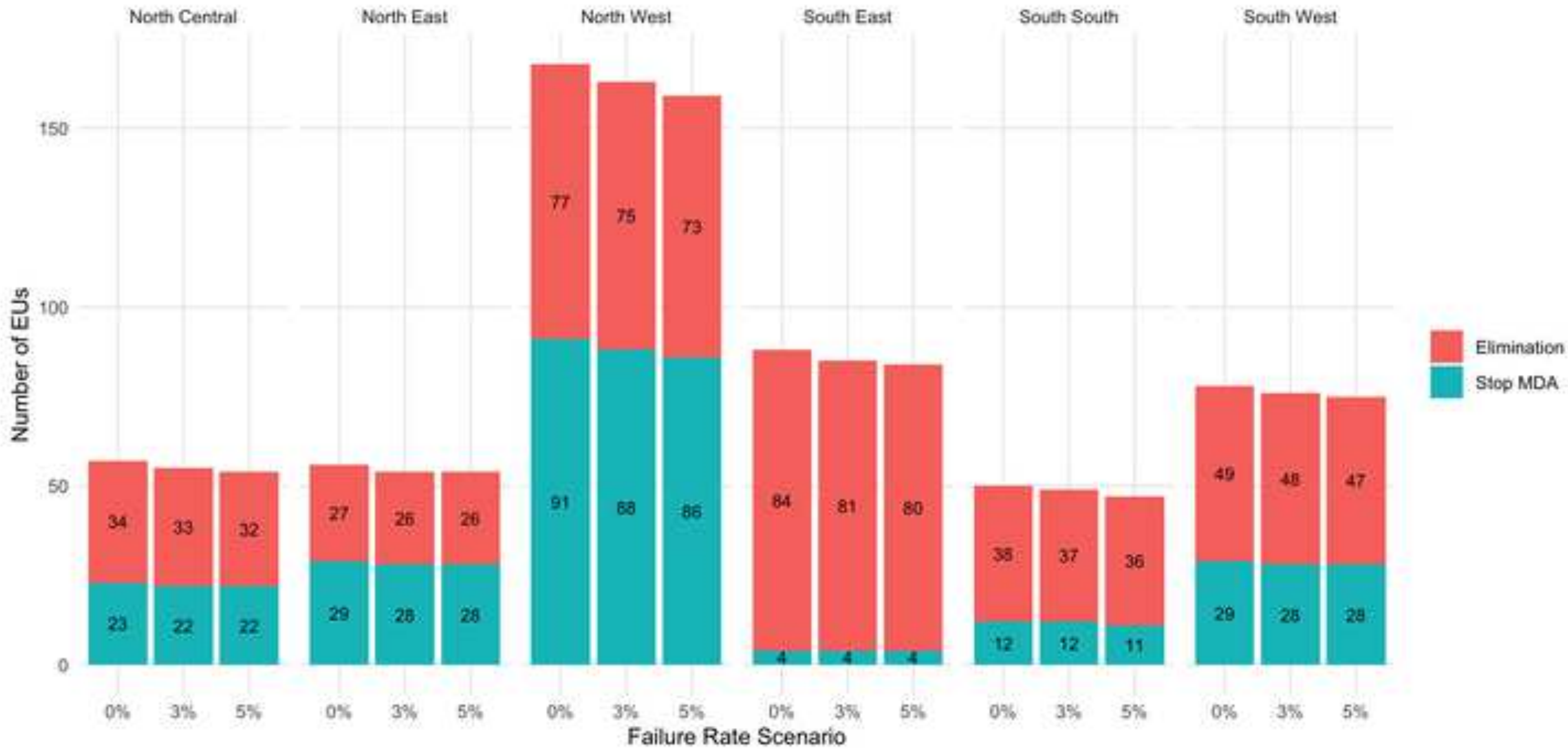

Click here to access/download;Figure;Fig 7.tif

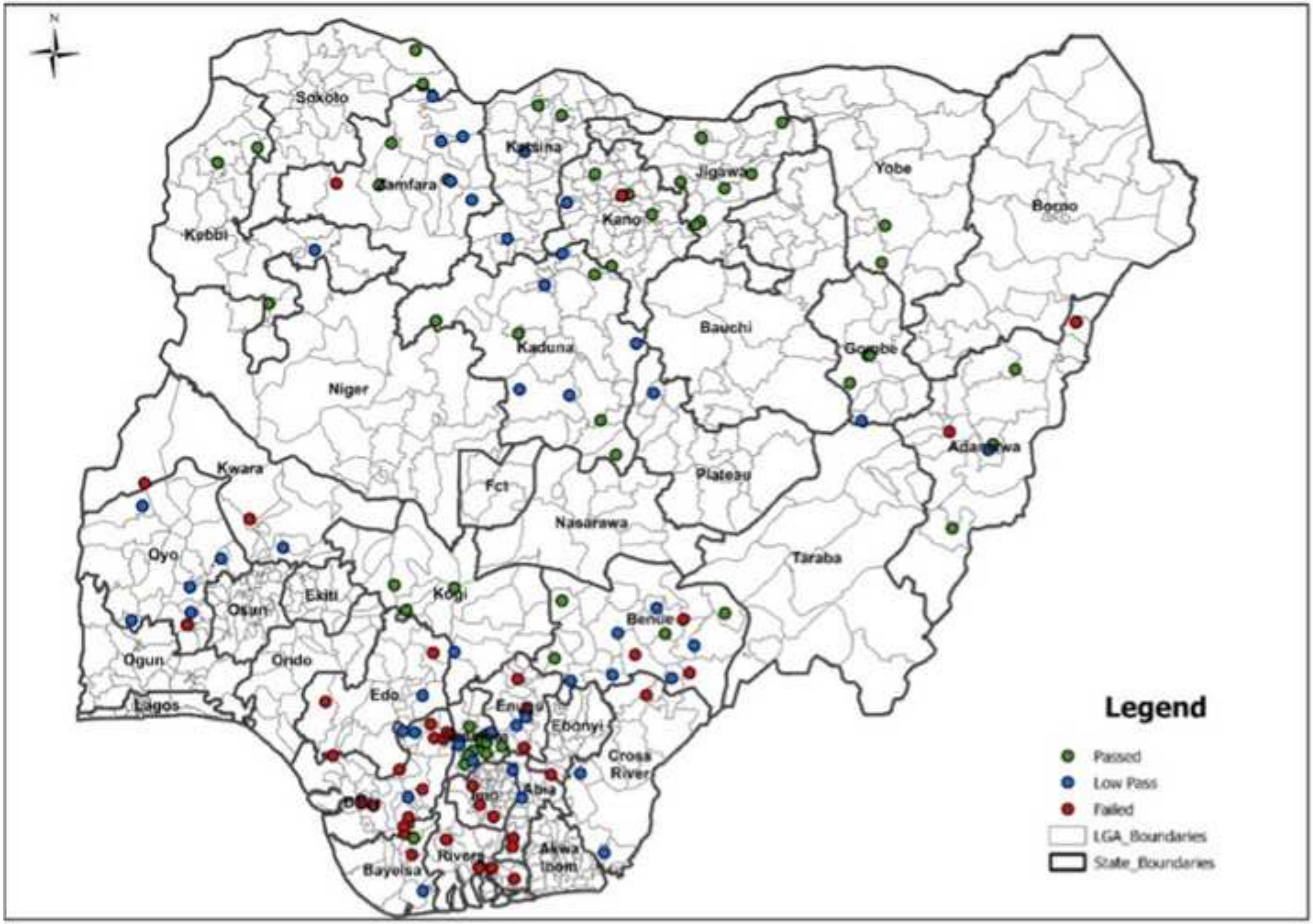

Click here to access/download;Figure;Fig 8.tif

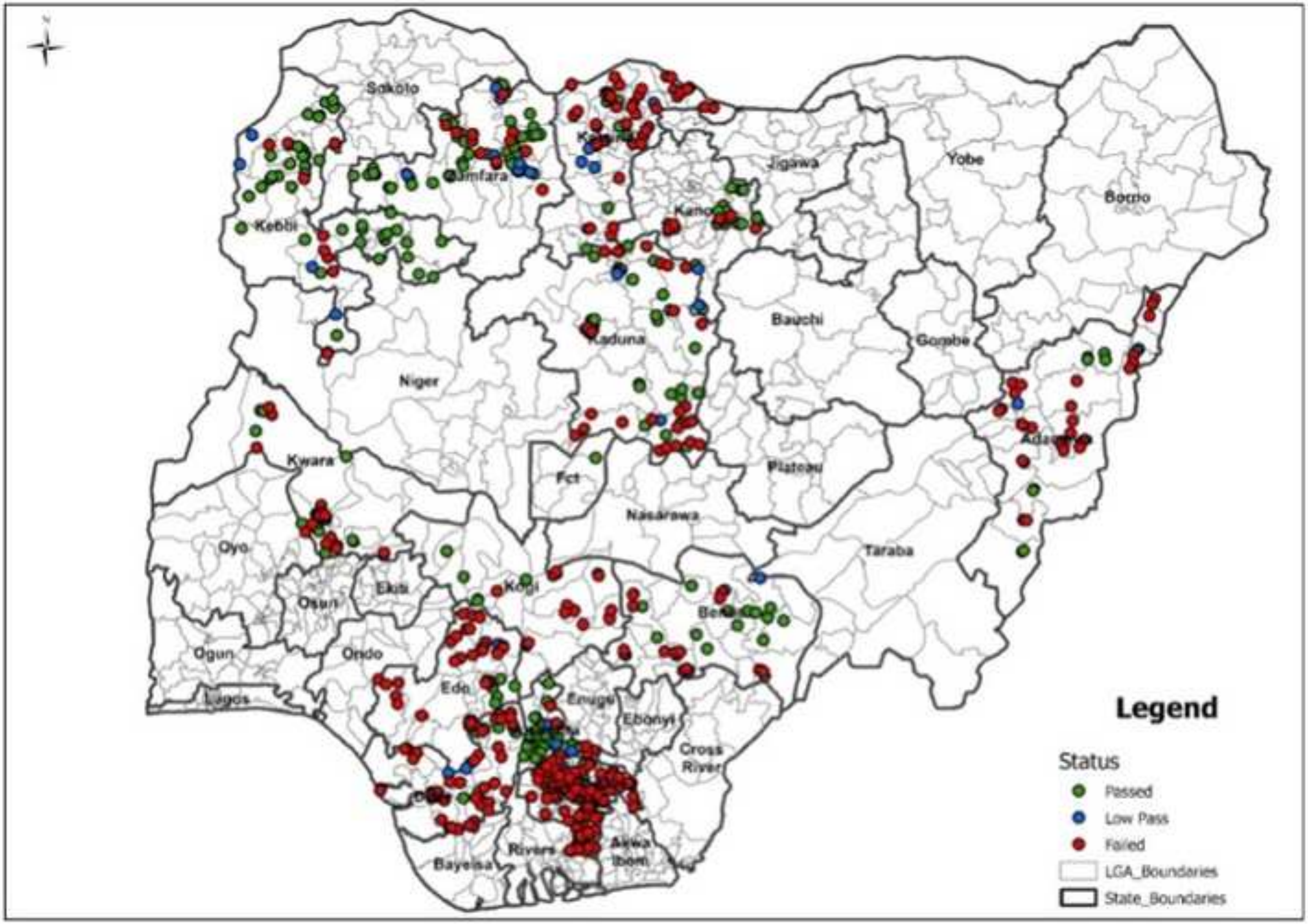

Click here to access/download;Figure;Fig 9.tif

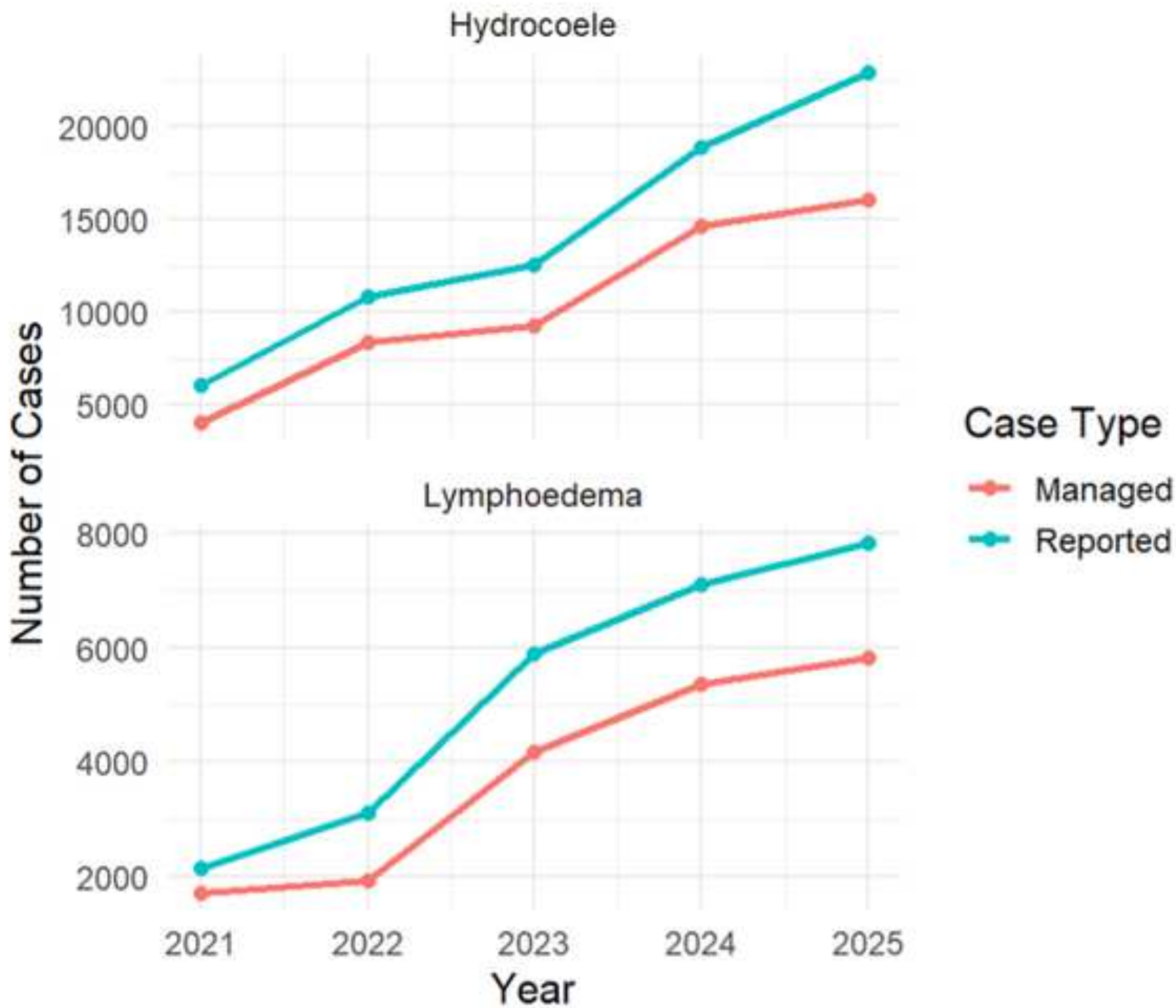

Click here to access/download
Supporting Information
S1 Dataset.csv

Click here to access/download
Supporting Information
S2 Dataset 2.xlsx

Response to reviewers

Journal comments

1) Your manuscript is missing the following sections: Discussion. Please ensure all required sections are present and in the correct order. Make sure section heading levels are clearly indicated in the manuscript text, and limit sub-sections to 3 heading levels. An outline of the required sections can be consulted in our submission guidelines here:
https://journals.plos.org/plosntds/s/submission-guidelines#loc-parts-of-a-submission

> R: We appreciate and agree with the editor that a Discussion section was missing from the original manuscript. In response, we have now included a Discussion section that interprets the findings, highlights their implications for the LF elimination programme, and discusses the study's strengths and limitations.

2) We have noticed that you have uploaded Supporting Information files, but you have not included a list of legends. Please add a full list of legends for your Supporting Information files after the references list.

> R: This observation is very helpful, and we appreciate it. We have provided a full list of legends for our supplementary files immediately after the references section.

3) Some material included in your submission may be copyrighted. According to PLOS's copyright policy, authors who use figures or other material (e.g., graphics, clipart, maps) from another author or copyright holder must demonstrate or obtain permission to publish this material under the Creative Commons Attribution 4.0 International (CC BY 4.0) License used by PLOS journals. Please closely review the details of PLOS's copyright requirements here: PLOS Licenses and Copyright. If you need to request permissions from a copyright holder, you may use PLOS's Copyright Content Permission form.

Please respond directly to this email and provide any known details concerning your material's license terms and permissions required for reuse, even if you have not yet obtained copyright permissions or are unsure of your material's copyright compatibility. Once you have responded and addressed all other outstanding technical requirements, you may resubmit your manuscript within Editorial Manager.

Potential Copyright Issues:

Figures 1, 2, 3, 7, and 8. Please (a) provide a direct link to the base layer of the map (i.e., the country or region border shape) and ensure this is also included in the figure legend; and (b) provide a link to the terms of use / license information for the base layer image or shapefile. We cannot publish proprietary or copyrighted maps (e.g. Google Maps, Mapquest) and the terms of use for your map base layer must be compatible with our CC BY 4.0 license.
Note: if you created the map in a software program like R or ArcGIS, please locate and indicate the source of the basemap shapefile onto which data has been plotted.

> R: We have revised our legends, accordingly, stating that not all the maps are copyrighted, and they were all created using open-source software shapefiles and from primary data generated by the authors. We have cited the data used, and provided it as a supplementary file, we also listed the link to the shapefiles used and also, we provided the license under which this images can be used and reshared

Reviewers' comments:

Reviewer #1:

1) Although the authors have explained the points well, would like to suggest them to revisit table 6 and explain how will the health system manage those challenges. While developing hypothesis that the EUs will pass TAS1, it would be wise to come up with their logic and assumptions.

R: We thank the reviewer for this insightful comment. We have revised the manuscript accordingly. First, we expanded the Discussion section and relocated relevant text from Table 6 to provide a clearer explanation of how the programme and health system are currently managing the identified challenges (see lines 1516-1567). Specifically, we describe ongoing strategies such as adaptive implementation approaches in insecure areas, integration of NTD activities within broader primary healthcare and Universal Health Coverage (UHC) platforms, strengthened partner coordination, resource sharing, and the use of coverage evaluation surveys to identify and address implementation bottlenecks, missed populations, and treatment non-compliance.

Second, we revised the methodology and clarified the rationale and assumptions underlying our projection that most Evaluation Units (EUs) will successfully pass TAS-1 by 2030 (see lines 322-339). This projection is based on the substantial progress already achieved by the programme and the relatively low Evaluation Mapping Survey (EMS) failure rate of 3.4% observed nationally. If this failure rate remains unchanged or even increases modestly (e.g., to 5%), our analysis suggests that most EUs would still be meet TAS-1 requirements before 2030, provided that current implementation efforts are sustained. The few EUs projected to remain at risk are those affected by persistent insecurity, delayed initiation of MAM, or repeated interruptions in programme implementation.

2) Second, areas which are not in the insecurity areas, can the team share information about the never-treated population or people who are missed during the MAM. What would be the strategies through which they will ensure higher drug compliance.

R: We thank the reviewer for this valuable suggestion. We currently do not have data documenting the never treated populations. These data are not routinely collected as part of national programs and only collected during operational research. However, we have revised the manuscript accordingly (see lines 1536-1567). to show how funding and operational constraints can affect the quality of MAM implementation and delay the conduct of coverage evaluation surveys and epidemiological assessments which are the source of these kinds of data. We highlighted that coverage evaluation surveys are routinely used to identify implementation bottlenecks, including missed populations, systematic non-compliance, and other factors limiting programme coverage.

We further clarified that several strategies are already being implemented to address these challenges (see lines 1551-1567).. These include increased integration of NTD activities within broader primary healthcare and Universal Health Coverage (UHC) platforms to support domestic resource mobilization and improve programme sustainability. In addition, ongoing collaboration among implementing partners, including joint planning, resource sharing, coordinated funding allocation, and technical support to underserved states, has facilitated the timely conduct of implementation and coverage surveys, enabling programmes to identify gaps in coverage, adjust implementation plans, strengthen community engagement, and improve treatment compliance. These approaches are increasingly being used to enhance programme performance and support progress toward elimination.

3) Also, there is limited information about the vector density and vector control initiatives. Is there any co-relation between the use and non-use of ITN (bed nets) in the population.

R: We thank the reviewer for this observation. These data are not collected as part of NFLEP, because vector control is currently not among the objectives of the programme. However, these data are available from the malaria elimination program. Hence, we have included relevant data from the Demographic health survey on bednet ownership and utilization across the country for the year 2025. We have expanded the methods, results and discussion section to accommodate this revision. Please

see lines : 293-301 (methods) 1284-1314 (results) ;1569-1580 (discussion). Specifically, we have avoided attributing the progress/setbacks faced by the NLFEP to bednet ownership or utilization, rather we emphasized the spread of these interventions, and the complementary role it could have on NLFEP if coverage and usage is optimized.

4) Regarding funding gap, there are no potential solutions proposed for the future. Rest all is great.

R: We thank the reviewer for this important observation. We have revised the manuscript to further discuss potential approaches for addressing future funding gaps (see lines 1534-1550). Specifically, we highlighted the increasing efforts to mainstream MAM and other NTD interventions within broader primary healthcare and Universal Health Coverage (UHC) platforms as a mechanism for improving domestic resource mobilization and reducing reliance on external funding. We also discussed ongoing collaborative arrangements among implementing partners, including joint planning, resource sharing, coordinated allocation of available resources, and technical support to states with limited funding or without established partners. These strategies are already being utilized within the programme and represent practical pathways for strengthening the sustainability of lymphatic filariasis elimination efforts in Nigeria.

5) Overall, good. It would have been helpful if there would have been some more information about the Ag/Mf positive individuals i.e., did they take part in the MAM in the past or not. Any clustering of positive villages/zones could have been highlighted.

R: We thank the reviewer for this insightful comment. We agree that such analyses would provide valuable insights into the drivers of persistent transmission. Unfortunately, the data available for the present review did not include individual-level information on prior participation in MAM, demographic characteristics, migration history, or other risk factors among Ag/Mf-positive individuals. Consequently, we were unable to conduct further analyses to determine whether positive individuals had previously received MAM or to assess clustering patterns based on individual demographic or migratory profiles.

Findings from recent confirmatory mapping study conducted in Nigeria (cited in our study as ref 26) have suggested that many Ag-positive children had no prior history of participation in MAM and were residing in communities characterized by suboptimal ITN coverage. However, because these data were not collected as part of the present review, we have refrained from making direct inferences regarding the Ag/Mf-positive individuals identified in this study.

6) The paper has great information about the program from its inception. The data is also very well captured. For the future projections, they need to identify solutions to the existing problems.

R: We thank the reviewer for this observation. This concern has been addressed in the revised Discussion section (see lines 1507-1517; 154-1550), where we discuss potential solutions to the major challenges identified and their implications for achieving the projected elimination milestones.

7) Secondly, for TAS3 pass geographies, they need to specify the post elimination surveillance strategies especially for the migrant population.

R: We thank the reviewer for this important observation. We have provided a new subsection in the results section on post-elimination surveillance strategies detailing the current state of PES in the country. Please see lines 1262-1268. We also revised the discussion (see lines 1565-1573) to highlight gaps and proffer recommendations.

Reviewer #2:

1 .Abstracts:

a. Background: The use of pronouns is not good in scientific reports, the sentence beginning" Here we" should be rephrased to discard the pronoun "we". This is applicable to all sentences with pronouns.

R: This has been revised. Thank you.

b.Methods

Discard the word "we", you can rephrase to "programmatic data was extracted from the National Lymphatic Filariasis Elimination…………….

R: This has been revised. Thank you.

c. Conclusion not supported by figures or facts. Revise

R: We thank the reviewer for this observation. We have revised the abstract conclusion to more closely reflect the findings presented in the study and to avoid introducing recommendations that were not directly supported by the results. The revised conclusion now emphasizes the documented programmatic achievements, remaining challenges, and the projected trajectory toward achieving the WHO 2030 elimination targets.

2.Introduction

a. Line 30, include WHO LF elimination conditions …. Rephrase sentence.

R: This has been revised. Thank you.

b. Citations inadequate, each sentence should be cited to minimize or avoid plagiarism. E.g sentences beginning lines 102 1nd 103.

R: This has been revised. Thank you.

c. Lines 105 and 112 don't begin a sentence with an acronym eg LF and GPELF write in full

R: This has been revised. Thank you.

d. No citation for sentence ending line 117,121,132

R: This has been revised. Thank you.

e. Sentence ending line 138, 142,154,159,161,163,164,166,174,175, 177 has no citation

R: This has been revised. Thank you.

f. line 144 and 146 have same citation consecutively [13], same citations should not follow each other.

R: This has been revised. Thank you.

g. Line 172, EUs should be written in full

R: This has been revised. Thank you.

h. Some sentences are abnormally lengthy eg. Sentence beginning line 183 and 3nding line 186. Rephrase, split and include correct citations.

R: This has been revised as advised. Thank you.

3. MethoDs

a. Rephrase sentence beginning line 192, 196,200,212, 214 to eliminate "We" which is not appropriate for a scientific report

R: This has been revised as advised. Thank you.

b. LGAs at beginning of sentence should be written in full. Line 198, 207

R: This has been revised as advised. Thank you.

c. Some sentences are abnormally lengthy eg line 214 to 218. Split long sentences.

R: This has been revised as advised. Thank you.

D. What is the study design?

R: We thank the reviewer for this comment. We have revised this section carefully and provided sub-section heads. Please see lines 266-273.

Results

a. NFLEP in line 228 to written in full.

R: We have revised this by adding "The".

b. Line 226 Nigeria's Lymphatic Filariasis Intervention Strategy. The contents do not seam to reflect results but methodology and discussion. What are your findings under the subtitle?

R: We have revised this section to provide more clarity. As stated above, this manuscript presents a retrospective review and synthesis of national LF programme records. Consequently, the Results section includes both descriptive narrative findings related to programme implementation and quantitative analyses of programme performance. The material presented under this subtitle reports findings from the programmatic review and provides essential context for interpreting subsequent epidemiological and operational outcomes. It does not describe the methods used to conduct the study, nor does it represent an interpretation of the findings. We have clarified this reporting approach in the "Data presentation" subsection under the methods section by explicitly stating that results are presented using a narrative synthesis approach alongside quantitative findings. Please see lines 364-370

c. The sentence beginning line 228 is a discussion while line 232 is methodology, rephrase

R: As described above, this manuscript adopts a narrative synthesis approach to present findings from the retrospective programmatic review. The referenced text forms part of the descriptive findings from programme records and implementation history and is intended to

provide context for understanding subsequent quantitative findings. It does not describe the methodology used to conduct the study, nor does it represent interpretation of the findings. We have nevertheless revised the methodology to improve clarity.

d. Line 236 to line 280 are not results but discussion, swap

R: As described above, the Results section incorporates both descriptive narrative findings and quantitative analyses. The material between lines 236 and 280 are narratives that provides the operational context necessary for understanding programme performance and elimination progress and are not intended as interpretation of the findings. However, we agree that a dedicated Discussion section was missing from the original manuscript and have now included an extensive one to provide interpretation of the findings and their implications.

e. Your results begin line 282.

R: As stated above, this manuscript presents a retrospective review and synthesis of national LF programme records. Consequently, the Results section includes both descriptive narrative findings related to programme implementation and quantitative analyses of programme performance. The material preceding line 282 reports findings from the programmatic review and provides essential context for interpreting subsequent epidemiological and operational outcomes. We have clarified this reporting approach in the Methods section by explicitly stating that results are presented using a narrative synthesis approach alongside quantitative findings.

f. All the Tables are not standard scientific tables, revise.

R: This has been revised. We have retained only the bottom and top line borders. Thank you.

4.Discussion

Although, results and discussions are mixed, their separation will improve the quality of this piece.

R: We also agree with the reviewer that a Discussion section was missing from the original manuscript. In response, we have now included a Discussion section that interprets the findings, highlights their implications for the LF elimination programme, and discusses the study's strengths and limitations.

Recommendation

This is an excellent piece. Revise the observations above and separate your results from discussion to reveal your findings clearly unless the journal allows this mix.

R: This has been revised as advised. Thank you.

Click here to access/download
Revised Article with Changes Highlighted
Manuscript_track.docx
