# Supplementary material for: Two decades of lymphatic filariasis elimination efforts in Nigeria: History, progress, challenges, and prospects for achieving the 2030 targets
Source: PLoS Negl Trop Dis. 2026 Nov 10;20(11):e0014860. doi: 10.1371/journal.pntd.0014860 (PMC13766621; doi:10.1371/journal.pntd.0014860)
Supplement: Supplementary file 2 [file pntd.0014860.s003.docx]

1.Abstracts:

a. Background: The use of pronouns is not good in scientific reports, the sentence beginning” Here we” should be rephrased to discard the pronoun “we”. This is applicable to all sentences with pronouns.

b.Methods

Discard the word “we”, you can rephrase to “programmatic data was extracted from the National Lymphatic Filariasis Elimination…………….

C Conclusion not supported by figures or facts. Revise

2.Introduction

a. Line 30, include WHO LF elimination conditions …. Rephrase sentence.

b. Citations inadequate, each sentence should be cited to minimize or avoid plagiarism. E.g sentences beginning lines 102 1nd 103.

c. Lines 105 and 112 don’t begin a sentence with an acronym eg LF and GPELF write in full

d. No citation for sentence ending line 117,121,132

e. Sentence ending line 138, 142,154,159,161,163,164,166,174,175, 177 has no citation

f. line 144 and 146 have same citation consecutively [13], same citations should not follow each other.

g. Line 172, EUs should be written in full

h. Some sentences are abnormally lengthy eg. Sentence beginning line 183 and 3nding line 186. Rephrase, split and include correct citations.

3. Methos

a. Rephrase sentence beginning line 192, 196,200,212, 214 to eliminate “We” which is not appropriate for a scientific report

b. LGAs at beginning of sentence should be written in full. Line 198, 207

c. Some sentences are abnormally lengthy eg line 214 to 218. Split long sentences.

D. What is the study design?

Results

a. NFLEP in line 228 to written in full.
b. Line 226 Nigeria’s Lymphatic Filariasis Intervention Strategy. The contents do not seam to reflect results but methodology and discussion. What are your findings under the subtitle?
c. The sentence beginning line 228 is a discussion while line 232 is methodology, rephrase
d. Line 236 to line 280 are not results but discussion, swap
e. Your results begin line 282.
f. All the Tables are not standard scientific tables, revise.

4.Discussion

Although, results and discussions are mixed, their separation will improve the quality of this piece.

Recommendation

This is an excellent piece. Revise the observations above and separate your results from discussion to reveal your findings clearly unless the journal allows this mix.
