# Supplementary material for: Two decades of lymphatic filariasis elimination efforts in Nigeria: History, progress, challenges, and prospects for achieving the 2030 targets
Source: PLoS Negl Trop Dis. 2026 Nov 10;20(11):e0014860. doi: 10.1371/journal.pntd.0014860 (PMC13766621; doi:10.1371/journal.pntd.0014860)
Supplement: Supplementary file 3 [file pntd.0014860.s004.docx]

**Response to reviewers**

**Journal comments**

1) Your manuscript is missing the following sections: Discussion.  Please ensure all required sections are present and in the correct order. Make sure section heading levels are clearly indicated in the manuscript text, and limit sub-sections to 3 heading levels. An outline of the required sections can be consulted in our submission guidelines here:
<https://journals.plos.org/plosntds/s/submission-guidelines#loc-parts-of-a-submission>

R: We appreciate and agree with the editor that a Discussion section was missing from the original manuscript. In response, we have now included a Discussion section that interprets the findings, highlights their implications for the LF elimination programme, and discusses the study's strengths and limitations.

2) We have noticed that you have uploaded Supporting Information files, but you have not included a list of legends. Please add a full list of legends for your Supporting Information files after the references list.

R: This observation is very helpful, and we appreciate it. We have provided a full list of legends for our supplementary files immediately after the references section.

3) Some material included in your submission may be copyrighted. According to PLOS’s copyright policy, authors who use figures or other material (e.g., graphics, clipart, maps) from another author or copyright holder must demonstrate or obtain permission to publish this material under the Creative Commons Attribution 4.0 International (CC BY 4.0) License used by PLOS journals. Please closely review the details of PLOS’s copyright requirements here: PLOS Licenses and Copyright. If you need to request permissions from a copyright holder, you may use PLOS's Copyright Content Permission form.

Please respond directly to this email and provide any known details concerning your material's license terms and permissions required for reuse, even if you have not yet obtained copyright permissions or are unsure of your material's copyright compatibility. Once you have responded and addressed all other outstanding technical requirements, you may resubmit your manuscript within Editorial Manager.

Potential Copyright Issues:

Figures 1, 2, 3, 7, and 8. Please (a) provide a direct link to the base layer of the map (i.e., the country or region border shape) and ensure this is also included in the figure legend; and (b) provide a link to the terms of use / license information for the base layer image or shapefile. We cannot publish proprietary or copyrighted maps (e.g. Google Maps, Mapquest) and the terms of use for your map base layer must be compatible with our CC BY 4.0 license.
Note: if you created the map in a software program like R or ArcGIS, please locate and indicate the source of the basemap shapefile onto which data has been plotted.

R: We have revised our legends, accordingly, stating that not all the maps are copyrighted, and they were all created using open-source software shapefiles and from primary data generated by the authors. We have cited the data used, and provided it as a supplementary file, we also listed the link to the shapefiles used and also, we provided the license under which this images can be used and reshared

**Reviewers' comments:**

**Reviewer #1:**

1) Although the authors have explained the points well, would like to suggest them to revisit table 6 and explain how will the health system manage those challenges. While developing hypothesis that the EUs will pass TAS1, it would be wise to come up with their logic and assumptions.

R: We thank the reviewer for this insightful comment. We have revised the manuscript accordingly. First, we expanded the Discussion section and relocated relevant text from Table 6 to provide a clearer explanation of how the programme and health system are currently managing the identified challenges (see lines 1516-1567). Specifically, we describe ongoing strategies such as adaptive implementation approaches in insecure areas, integration of NTD activities within broader primary healthcare and Universal Health Coverage (UHC) platforms, strengthened partner coordination, resource sharing, and the use of coverage evaluation surveys to identify and address implementation bottlenecks, missed populations, and treatment non-compliance.

Second, we revised the methodology and clarified the rationale and assumptions underlying our projection that most Evaluation Units (EUs) will successfully pass TAS-1 by 2030 (see lines 322-339). This projection is based on the substantial progress already achieved by the programme and the relatively low Evaluation Mapping Survey (EMS) failure rate of 3.4% observed nationally. If this failure rate remains unchanged or even increases modestly (e.g., to 5%), our analysis suggests that most EUs would still be meet TAS-1 requirements before 2030, provided that current implementation efforts are sustained. The few EUs projected to remain at risk are those affected by persistent insecurity, delayed initiation of MAM, or repeated interruptions in programme implementation.

2) Second, areas which are not in the insecurity areas, can the team share information about the never-treated population or people who are missed during the MAM. What would be the strategies through which they will ensure higher drug compliance.

R: We thank the reviewer for this valuable suggestion. We currently do not have data documenting the never treated populations. These data are not routinely collected as part of national programs and only collected during operational research. However, we have revised the manuscript accordingly (see lines 1536-1567). to show how funding and operational constraints can affect the quality of MAM implementation and delay the conduct of coverage evaluation surveys and epidemiological assessments which are the source of these kinds of data. We highlighted that coverage evaluation surveys are routinely used to identify implementation bottlenecks, including missed populations, systematic non-compliance, and other factors limiting programme coverage.

We further clarified that several strategies are already being implemented to address these challenges (see lines 1551-1567).. These include increased integration of NTD activities within broader primary healthcare and Universal Health Coverage (UHC) platforms to support domestic resource mobilization and improve programme sustainability. In addition, ongoing collaboration among implementing partners, including joint planning, resource sharing, coordinated funding allocation, and technical support to underserved states, has facilitated the timely conduct of implementation and coverage surveys, enabling programmes to identify gaps in coverage, adjust implementation plans, strengthen community engagement, and improve treatment compliance. These approaches are increasingly being used to enhance programme performance and support progress toward elimination.

3) Also, there is limited information about the vector density and vector control initiatives. Is there any co-relation between the use and non-use of ITN (bed nets) in the population.

R: We thank the reviewer for this observation. These data are not collected as part of NFLEP, because vector control is currently not among the objectives of the programme. However, these data are available from the malaria elimination program. Hence, we have included relevant data from the Demographic health survey on bednet ownership and utilization across the country for the year 2025. We have expanded the methods, results and discussion section to accommodate this revision. Please see lines : 293-301 (methods) 1284-1314 (results) ;1569-1580 (discussion). Specifically, we have avoided attributing the progress/setbacks faced by the NLFEP to bednet ownership or utilization, rather we emphasized the spread of these interventions, and the complementary role it could have on NLFEP if coverage and usage is optimized.

4) Regarding funding gap, there are no potential solutions proposed for the future. Rest all is great.

R: We thank the reviewer for this important observation. We have revised the manuscript to further discuss potential approaches for addressing future funding gaps (see lines 1534-1550). Specifically, we highlighted the increasing efforts to mainstream MAM and other NTD interventions within broader primary healthcare and Universal Health Coverage (UHC) platforms as a mechanism for improving domestic resource mobilization and reducing reliance on external funding. We also discussed ongoing collaborative arrangements among implementing partners, including joint planning, resource sharing, coordinated allocation of available resources, and technical support to states with limited funding or without established partners. These strategies are already being utilized within the programme and represent practical pathways for strengthening the sustainability of lymphatic filariasis elimination efforts in Nigeria.

5) Overall, good. It would have been helpful if there would have been some more information about the Ag/Mf positive individuals i.e., did they take part in the MAM in the past or not. Any clustering of positive villages/zones could have been highlighted.

R: We thank the reviewer for this insightful comment. We agree that such analyses would provide valuable insights into the drivers of persistent transmission. Unfortunately, the data available for the present review did not include individual-level information on prior participation in MAM, demographic characteristics, migration history, or other risk factors among Ag/Mf-positive individuals. Consequently, we were unable to conduct further analyses to determine whether positive individuals had previously received MAM or to assess clustering patterns based on individual demographic or migratory profiles.

Findings from recent confirmatory mapping study conducted in Nigeria (cited in our study as ref 26) have suggested that many Ag-positive children had no prior history of participation in MAM and were residing in communities characterized by suboptimal ITN coverage. However, because these data were not collected as part of the present review, we have refrained from making direct inferences regarding the Ag/Mf-positive individuals identified in this study.

**6)** The paper has great information about the program from its inception. The data is also very well captured. For the future projections, they need to identify solutions to the existing problems.

R: We thank the reviewer for this observation. This concern has been addressed in the revised Discussion section (see lines 1507-1517; 154-1550), where we discuss potential solutions to the major challenges identified and their implications for achieving the projected elimination milestones.

7) Secondly, for TAS3 pass geographies, they need to specify the post elimination surveillance strategies especially for the migrant population.

R: We thank the reviewer for this important observation. We have provided a new subsection in the results section on post-elimination surveillance strategies detailing the current state of PES in the country. Please see lines 1262-1268. We also revised the discussion (see lines 1565-1573) to highlight gaps and proffer recommendations.

**Reviewer #2:**

1 .Abstracts:

a. Background: The use of pronouns is not good in scientific reports, the sentence beginning” Here we” should be rephrased to discard the pronoun “we”. This is applicable to all sentences with pronouns.

R: This has been revised. Thank you.

b.Methods

Discard the word “we”, you can rephrase to “programmatic data was extracted from the National Lymphatic Filariasis Elimination…………….

R: This has been revised. Thank you.

c. Conclusion not supported by figures or facts. Revise

R: We thank the reviewer for this observation. We have revised the abstract conclusion to more closely reflect the findings presented in the study and to avoid introducing recommendations that were not directly supported by the results. The revised conclusion now emphasizes the documented programmatic achievements, remaining challenges, and the projected trajectory toward achieving the WHO 2030 elimination targets.

2.Introduction

a. Line 30, include WHO LF elimination conditions …. Rephrase sentence.

R: This has been revised. Thank you.

b. Citations inadequate, each sentence should be cited to minimize or avoid plagiarism. E.g sentences beginning lines 102 1nd 103.

R: This has been revised. Thank you.

c. Lines 105 and 112 don’t begin a sentence with an acronym eg LF and GPELF write in full

R: This has been revised. Thank you.

d. No citation for sentence ending line 117,121,132

R: This has been revised. Thank you.

e. Sentence ending line 138, 142,154,159,161,163,164,166,174,175, 177 has no citation

R: This has been revised. Thank you.

f. line 144 and 146 have same citation consecutively [13], same citations should not follow each other.

R: This has been revised. Thank you.

g. Line 172, EUs should be written in full

R: This has been revised. Thank you.

h. Some sentences are abnormally lengthy eg. Sentence beginning line 183 and 3nding line 186. Rephrase, split and include correct citations.

R: This has been revised as advised. Thank you.

3. MethoDs

a. Rephrase sentence beginning line 192, 196,200,212, 214 to eliminate “We” which is not appropriate for a scientific report

R: This has been revised as advised. Thank you.

b. LGAs at beginning of sentence should be written in full. Line 198, 207

R: This has been revised as advised. Thank you.

c. Some sentences are abnormally lengthy eg line 214 to 218. Split long sentences.

R: This has been revised as advised. Thank you.

D. What is the study design?

R: We thank the reviewer for this comment. We have revised this section carefully and provided sub-section heads. Please see lines 266-273.

Results

a. NFLEP in line 228 to written in full.

R: We have revised this by adding “The”.

b. Line 226 Nigeria’s Lymphatic Filariasis Intervention Strategy. The contents do not seam to reflect results but methodology and discussion. What are your findings under the subtitle?

R: We have revised this section to provide more clarity. As stated above, this manuscript presents a retrospective review and synthesis of national LF programme records. Consequently, the Results section includes both descriptive narrative findings related to programme implementation and quantitative analyses of programme performance. The material presented under this subtitle reports findings from the programmatic review and provides essential context for interpreting subsequent epidemiological and operational outcomes. It does not describe the methods used to conduct the study, nor does it represent an interpretation of the findings. We have clarified this reporting approach in the “Data presentation” subsection under the methods section by explicitly stating that results are presented using a narrative synthesis approach alongside quantitative findings. Please see lines 364-370

c. The sentence beginning line 228 is a discussion while line 232 is methodology, rephrase

R: As described above, this manuscript adopts a narrative synthesis approach to present findings from the retrospective programmatic review. The referenced text forms part of the descriptive findings from programme records and implementation history and is intended to provide context for understanding subsequent quantitative findings. It does not describe the methodology used to conduct the study, nor does it represent interpretation of the findings. We have nevertheless revised the methodology to improve clarity.

d. Line 236 to line 280 are not results but discussion, swap

R: As described above, the Results section incorporates both descriptive narrative findings and quantitative analyses. The material between lines 236 and 280 are narratives that provides the operational context necessary for understanding programme performance and elimination progress and are not intended as interpretation of the findings. However, we agree that a dedicated Discussion section was missing from the original manuscript and have now included an extensive one to provide interpretation of the findings and their implications.

e. Your results begin line 282.

R: As stated above, this manuscript presents a retrospective review and synthesis of national LF programme records. Consequently, the Results section includes both descriptive narrative findings related to programme implementation and quantitative analyses of programme performance. The material preceding line 282 reports findings from the programmatic review and provides essential context for interpreting subsequent epidemiological and operational outcomes. We have clarified this reporting approach in the Methods section by explicitly stating that results are presented using a narrative synthesis approach alongside quantitative findings.

f. All the Tables are not standard scientific tables, revise.

R: This has been revised. We have retained only the bottom and top line borders. Thank you.

4.Discussion

Although, results and discussions are mixed, their separation will improve the quality of this piece.

R: We also agree with the reviewer that a Discussion section was missing from the original manuscript. In response, we have now included a Discussion section that interprets the findings, highlights their implications for the LF elimination programme, and discusses the study's strengths and limitations.

Recommendation

This is an excellent piece. Revise the observations above and separate your results from discussion to reveal your findings clearly unless the journal allows this mix.

R: This has been revised as advised. Thank you.
