# Supplementary material for: Two decades of lymphatic filariasis elimination efforts in Nigeria: History, progress, challenges, and prospects for achieving the 2030 targets
Source: PLoS Negl Trop Dis. 2026 Nov 10;20(11):e0014860. doi: 10.1371/journal.pntd.0014860 (PMC13766621; doi:10.1371/journal.pntd.0014860)
Supplement: Supplementary file 4 [file pntd.0014860.s006.docx]

Response to Reviewers comments

Reviewer #2: 

C1: Study design appropriate but not clearly state, type of review excluded, systematic, scoping, meta-analysis?

R1: We thank the reviewer for this observation. We have now revised the line to clarity the study design. This study was a **retrospective secondary analysis of national programmatic data**, supplemented by secondary analysis of population-based survey data

C2: Population not clealy defined

**Response:** Thank you for this observation. We agree that the populations represented in the programme data were not sufficiently described in the original Methods. Because this study analyzed routinely collected data generated across different components of the LF elimination programme, the population varied according to the specific programme activity. We have therefore added a “Study population and sample sizes” subsection to clarify the populations represented in baseline mapping, MAM, MMDP, epidemiological monitoring assessments, and TAS, including the applicable age groups.

C3: No evidence of sample size

**Response:** Thank you for this comment. A separate sample-size calculation was not performed for the present study because this was a retrospective secondary analysis of routinely collected national programme data, and all eligible programme records available during the study period were included. The individual epidemiological assessments were conducted as part of routine programme implementation, with their sample sizes determined at the time of implementation in accordance with the applicable WHO-recommended survey procedures. We have clarified this in the newly added “Study population and sample sizes” subsection of the Methods.

C6: No the tables are not appropriately labelled in the subtitles, strange numbers in brackets not defined

**Response:** Thank you for this observation. We have now revised all the tables and made sure they are properly labelled. Also, we have defined all the numbers in brackets.

C6: Conclusions do not reflect the findings, title, and objective of the study revision.

**Response:** Thank you for this observation. We have substantially revised the Conclusion to better reflect the study objectives and key findings, including the progress achieved through MAM and MMDP, the projected programme status by 2030 based on observed trends and current implementation schedules, and the remaining implementation and evaluation gaps. We have also highlighted the programme priorities required to consolidate these gains, including intensified interventions in lagging IUs, timely epidemiological assessments, strengthened surveillance, increased domestic ownership, strategic partnerships, and sustained political commitment. The revised Conclusion is now more closely aligned with the findings and overall focus of the study.

C7: No evidence of limitations analysis

**Response:** Thank you for this observation. We agree that the limitations of the study were not adequately addressed in the original manuscript. We have now added a dedicated limitations section discussing the potential limitations associated with the use of routinely collected programme data spanning more than two decades, changes in diagnostic tools and survey protocols over time, variations in programme implementation and partner support across states, and differences in the timing of epidemiological assessments across endemic IUs. We have also acknowledged the uncertainty associated with the 2030 projections and clarified that these estimates are based on observed programme trends and current implementation schedules and should not be interpreted as definitive predictions.

**Comments in the attached pdf**

C1: page 9 line 55- define EU at first mention

R1: We thank the reviewer for this observation. We have now defined it at first mention

C2: page 9 line 55- define IU at first mention

R2: We thank the reviewer for this observation. We have now defined it at first mention

C3: Page 19, line 284- Zones are not clearly defined on a map, so they are not clear to the reader. Use this map to show the zones.

R3: We thank the reviewer for this observation. We have now revised all the figures and included the zones. This is a very helpful comment.

C4: page 9,line 282- here and elsewhere, names must be consistent. Text, tables, and figures use mixed one- or two-word names.

R4: We thank the reviewer for this observation. We have revised this throughout the text and adopted the two-word name with a -, e.g., South-east

C5: Page 19, line 431: strike out in Nigeria

R5: We thank the reviewer for this observation. We have revised the text

C6: Page 32, line 516: needs a more accurate and descriptive caption. These are not rates.

R6: We thank the reviewer for this observation. We agree that referring to the values presented in Figure 4 as “rates” was inaccurate, as the bar graph presents counts of LGAs that experienced at least one EMS failure rather than rates. We have revised the caption to read: **“Number of Evaluation Units (EUs) with at least one failed Epidemiological Monitoring Survey (EMS), by state from 2000–2025.”**

C7: Page 33, line 58: replace the country with Nigeria

R7: We thank the reviewer for this observation. We have revised the text

C8: Page 42, line 693: include “from the” in the missing text

R8: We thank the reviewer for this observation. We have revised the text

C9: Page 42, line 706- strikeout endemic

R9: We thank the reviewer for this observation. We have revised the text

C10: page 42, line 71: trends and schedules typically result in inaccurate predictions of success, in part because transmission dynamics change as levels approach eradication.

**Response 10:** We agree that the original wording conveyed greater certainty than warranted. The estimates were intended as programme-based projections derived from observed trends and current implementation schedules, rather than definitive predictions of programme outcomes. We have revised the text to use conditional language and explicitly state that these projections assume that current progress is maintained and remain subject to changes in transmission dynamics and programme implementation.

C11; page 42: line 72: Stated as certain - is this not a prediction? Needs to be clarified.

**Response 11:** same as our response above

C12: page 43, line 729: clarify - what type(s) of insecurity?

R12: we have now clarified this. “Foremost among these is insecurity associated with terrorism, banditry, and communal clashes, which continues to restrict access to some communities and remains a major obstacle to the implementation of MAM, community sensitization activities, and epidemiological assessments”

C13: page 43, line 732: as above, clarify the nature of the insecurity

R13: we have now clarified this. “The consequences of these disruptions are particularly evident in northern Nigeria, especially in Borno State, where prolonged insecurity associated with terrorism and banditry delayed the initiation of MAM until 2019 and resulted in most endemic LGAs receiving only 1–4 effective treatment rounds”
